# Discovery of selective LATS inhibitors *via* scaffold hopping: enhancing drug-likeness and kinase selectivity for potential applications in regenerative medicine†

**DOI:** 10.1039/d4md00492b

**Published:** 2024-09-14

**Authors:** Guldana Issabayeva, On-Yu Kang, Seong Yun Choi, Ji Young Hyun, Seong Jun Park, Hei-Cheul Jeung, Hwan Jung Lim

**Affiliations:** a Data Convergence Drug Research Center, Korea Research Institute of Chemical Technology 141 Gajeong-ro Daejeon 34114 Republic of Korea sjunpark@krict.re.kr indium@krict.re.kr; b Department of Medicinal Chemistry and Pharmacology, University of Science & Technology 217 Gajeong-ro Daejeon 34113 Republic of Korea; c Department of Medical Oncology, Yonsei University College of Medicine 211 Eonju-ro, Gangnam-gu Seoul 06273 Republic of Korea jeunghc1123@yuhs.ac

## Abstract

Due to its essential roles in cell proliferation and apoptosis, the precise regulation of the Hippo pathway through LATS presents a viable biological target for developing new drugs for cancer and regenerative diseases. However, currently available probes for selective and highly drug-like inhibition of LATS require further improvement in terms of both activity, selectivity and drug-like properties. Through scaffold hopping aided by docking studies and AI-assisted prediction of metabolic stabilities, we successfully identified an advanced LATS inhibitor demonstrating potent kinase activity, exceptional selectivity against other kinases, and superior oral pharmacokinetic profiles.

## Abstract 2

Using scaffold hopping aided by docking studies and AI-assisted metabolic stability predictions, we identified a novel LATS inhibitor with potent kinase activity, high selectivity, and superior oral pharmacokinetic profiles.

## Graphical abstract

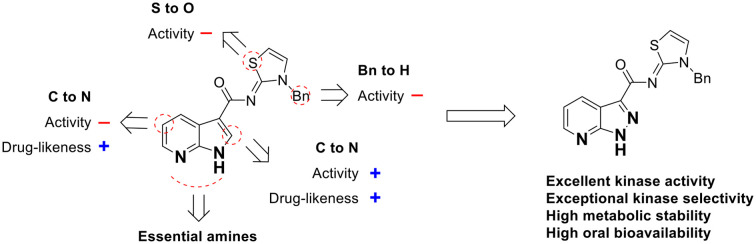

## 1. Introduction

The Hippo pathway,^1,2^ initially identified in *Drosophila*, plays a critical role in governing organ size and tissue homeostasis by modulating cell proliferation and apoptosis.^3–11^ Core components of the Hippo pathway include the large tumor suppressor kinases (LATS1 and LATS2), which phosphorylate and inhibit the transcriptional co-activators YAP (Yes-associated protein) and TAZ (transcriptional coactivator with PDZ-binding motif).^12^ Dysregulation of the Hippo pathway has been implicated in various human diseases, including cancer and regenerative disorders.^13^

Recent studies have shed light on the potential therapeutic utilities of targeting the Hippo pathway for promoting tissue regeneration.^14–20^ Among the key effectors of this pathway, LATS kinases have received significant attention as attractive pharmacological targets.^21^ Inhibition of LATS activity has been shown to unleash the regenerative potential of stem and progenitor cells, facilitating tissue repair and regeneration in preclinical models of diverse pathological conditions.^22–26^

Harnessing LATS inhibition as a therapeutic strategy for regenerative diseases presents both challenges and opportunities. Key challenges include the need for improved kinase selectivity and the development of drugs with good pharmacokinetic profiles. Finally, we believe that the development of exceptionally selective LATS inhibitors with good pharmacokinetic properties can pave the way to apply them from bench to bedside.

**Fig. 1:**
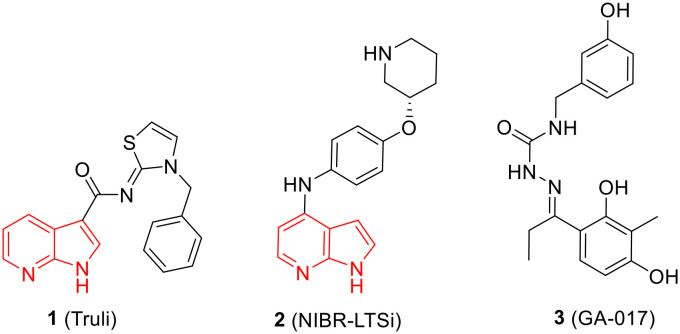
Structures of the reported LATS inhibitors.

Until now, several LATS inhibitors have been discovered.^23–27^ Mostly, the reported compounds have pyrrolo[2,3-*b*]pyridine (7-azaindole) structure^23,24,26^ (Fig. 1). Existing LATS inhibitors often suffer from limitations such as a lack of kinase selectivity, poor metabolic stability, and low water solubility, hampering the further development of these compounds for target diseases. Especially, low pharmacokinetic profiles due to poor metabolic stability^24^ are one of the major issues to overcome in developing these compounds for clinical purposes. To address these challenges, the development of novel LATS kinase inhibitors with enhanced drug-like properties and improved selectivity has become imperative.

In this study, we report the design, synthesis, and characterization of a novel class of LATS kinase inhibitors. Employing rational drug design strategies centered on scaffold hopping,^28,29^ our aim was to improve drug-likeness while maintaining high potency and selectivity of these inhibitors, thereby addressing the limitations of previous compounds.^23,24^ Our findings open avenues for exploring LATS pharmacological modulators as targeted therapeutic approaches for treating cancer and other diseases characterized by dysregulated cell proliferation and survival.

## 2. Results & discussion

**Fig. 2:**
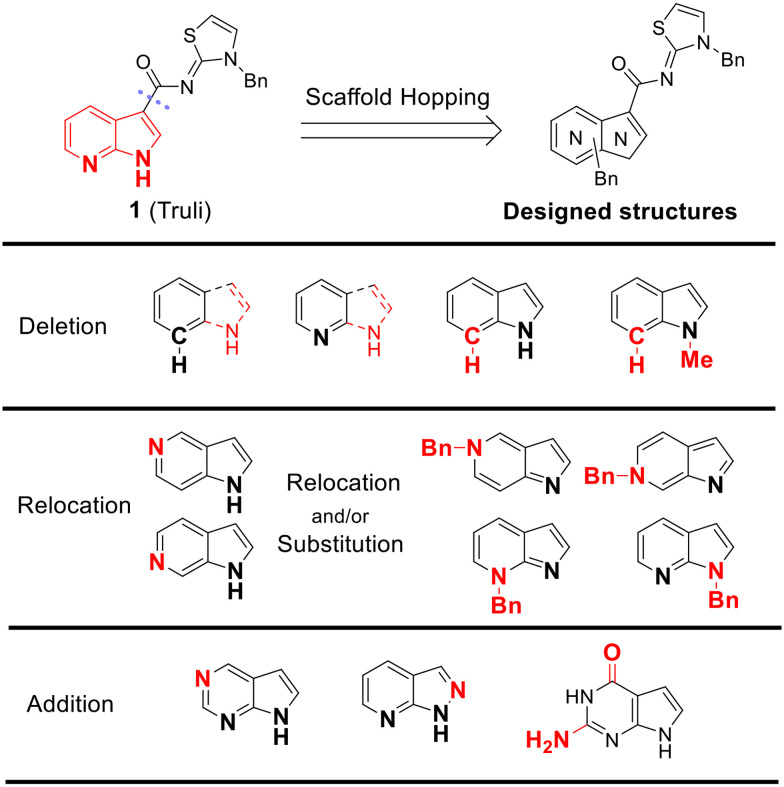
Types of the designed structures.

To identify an optimal core structure with potent inhibitory activity, excellent kinase selectivity, and drug-likeness, we systematically designed potential structures distinct from the 1*H*-pyrrolo[2,3-*b*]pyridine utilized in prior studies.^23,24^ Initially, we dissected the reported inhibitor 1 into its bicyclic core and the carboxamide group at the 3-position (Fig. 2). Subsequently, 1*H*-pyrrolo[2,3-*b*]pyridine was substituted by various 5,6-bicyclic heterocycles^30,31^ to elucidate crucial structural features for effective LATS inhibition. Modifications such as ring deletions, amine relocations or substitutions, and heteroatom additions were implemented to craft alternative scaffolds.

We conducted subsequent molecular docking studies to compare the binding pose and predict affinities of the designed compounds.^32,33^

Additionally, we used PredMS, an AI-based tool developed by KRICT (Korea Research Institute of Chemical Technology), to assess the predicted metabolic stability of the compounds. PredMS is a web-based model designed to support early drug discovery for small organic molecules (https://predms.netlify.app/).^34^ According to the output, a compound is considered ‘stable’ if more than 50% of it remains after 30 minutes in human liver microsomes. A value closer to one indicates higher stability.

**Fig. 3:**
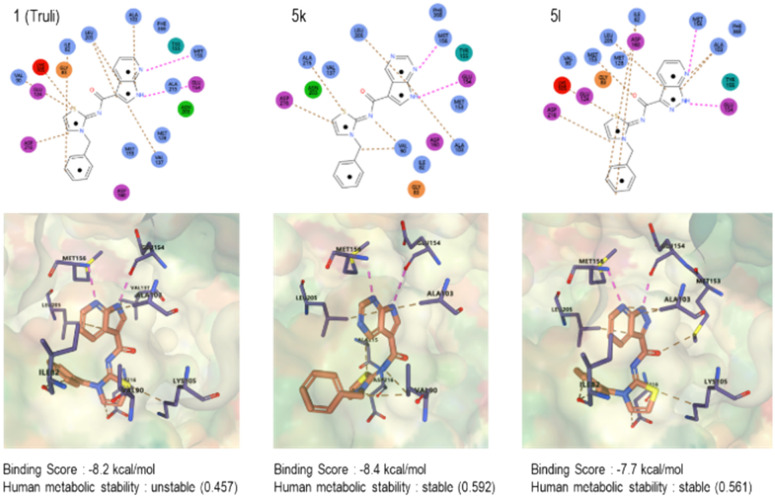
Docking simulation and predicted human metabolic stabilities of 1, 5k and 5l.

Due to the unavailability of a reported crystal structure of a LATS-inhibitor complex, the most similar structure, the ROCK1-7-azaindole crystal structure,^35^ was utilized for docking simulations to predict the binding pose of the designed compounds to further compare them with Truli's reported binding interactions (Fig. 3). 1, Truli, as reported,^23^ exhibited a favorable binding score (−8.2 kcal mol^−1^) and moderate human metabolic stability (0.457). The presence and positioning of both amines in the pyrrolopyridine ring appeared crucial for achieving a good binding pose. In this molecular docking simulation model, the pyrrolopyridine ring forms a bidentate hydrogen bond with the backbone hinge residues Met156 and Glu154. These hydrogen bond interactions are similarly preserved in the pyrrolopyrimidine of compound 5k and the pyrazolopyridine of compound 5l, indicating that the addition of an amine to the pyrrolopyridine ring retains a favorable binding pose. The thiazolamine moiety is predicted to interact with Asp216 in 1 which is also the case for 5k and 5l. Moreover, the benzyl group interacts with Ile82 in both 1 and 5l, although these van der Waals interactions were not observed in 5k.

**Scheme 1:**
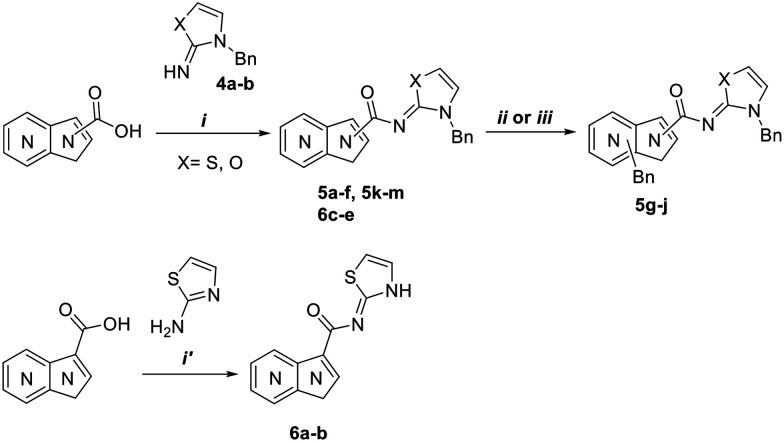
Reagents and conditions: (i) HATU, DIPEA, DMF, RT; (i′) HBTU, pyridine, 80–100 °C; (ii) NaH, benzyl bromide, DMF, RT; (iii) benzyl bromide, DMF, 60 °C.

The designed compounds 5a–m and further derivatized compounds 6a–e were synthesized from commercially available carboxylic acids with 2-aminothiazole along with *N*-substituted thiazol-2(3*H*)-imine 4a and oxazol-2(3*H*)-imine 4b using the reported procedures^36,37^ (Scheme 1). The desired compounds were thoroughly analyzed by ^1^H NMR and LC–MS for structural confirmation. The purity of the synthesized compounds was assessed using HPLC.

The *in vitro* kinase inhibitory activities of the new compounds were carefully assessed (Table 1). Monocyclic structures, such as phenyl- and 3-pyridyl-substituted compounds 5a–b, completely lost their inhibitory activities upon deletion (entries 2–3). Similarly, bicyclic structures lacking an amine in the 6-membered ring, such as indole structures 5c–d (entries 4–5), showed negligible inhibition, regardless of the presence of an *N*-substituent in the 5-membered ring.

**Table 1:** Synthesized structures and the percent enzymatic activities

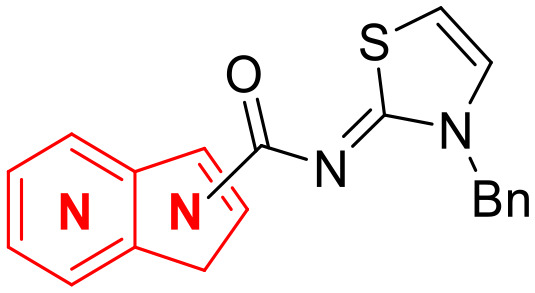				
Entry	Compound	Scaffold	% enzymatic activity (at 1 μM of a compound)	
			LATS1(h)	LATS2(h)
1	1 (Truli)	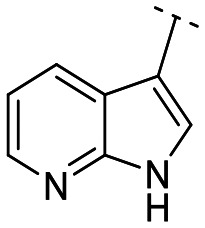	3 ± 1	1 ± 1
2	5a	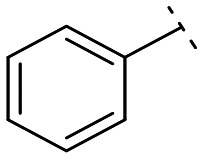	106 ± 0	101 ± 4
3	5b	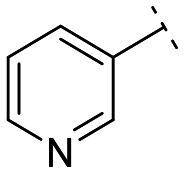	134 ± 2	109 ± 1
4	5c	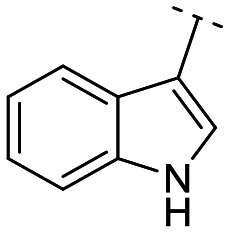	145 ± 15	92 ± 7
5	5d	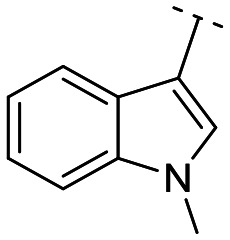	103 ± 2	90 ± 0
6	5e	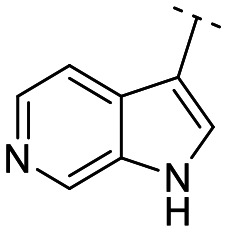	114 ± 10	109 ± 7
7	5f	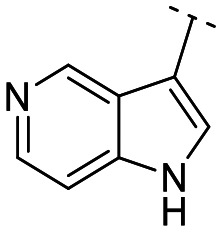	111 ± 1	102 ± 7
8	5g	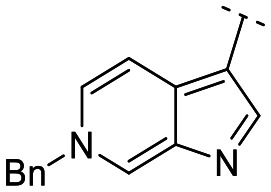	106 ± 2	86 ± 4
9	5h	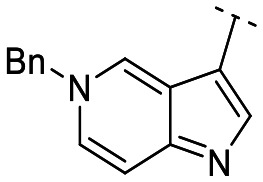	104 ± 0	104 ± 0
10	5i	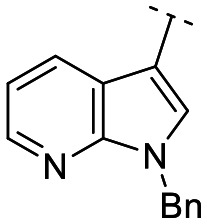	94 ± 4	55 ± 1
11	5j	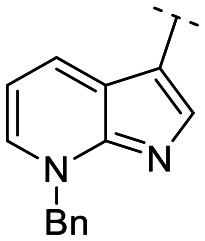	57 ± 10	23 ± 3
12	5k	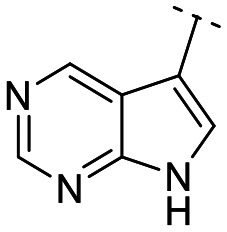	21 ± 2	21 ± 2
13	5l	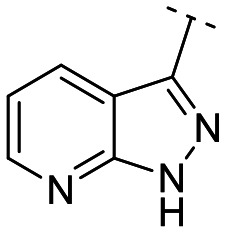	8 ± 0	1 ± 1
14	5m	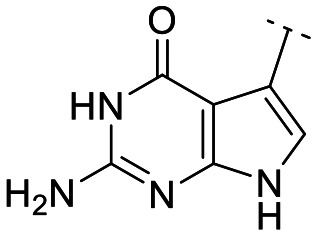	99 ± 4	111 ± 2

Subsequently, the importance of the position and substitution of amines in the 6-membered ring was investigated. Unlike the original structure of 1*H*-pyrrolo[2,3-*b*]pyridine (1), which bears an *N* at the 7-position in the 6-membered ring, both relocation structures with *N* at the 6-position (1*H*-pyrrolo[2,3-*c*]pyridine, 5e) and at the 5-position (1*H*-pyrrolo[3,2-*c*]pyridine, 5f) significantly diminished their inhibitory activities, irrespective of the presence of an *N*-substituent (entries 6–9). However, substitutions without relocating the original *N*-position showed decreased activities, but not universally (entries 10–11).

In summary, the positions of the two amines in 5,6-bicyclic heterocycles were crucial for demonstrating potent inhibitory activities, as confirmed by docking studies using similar X-ray structures. The data is available in ESI.†

We further explored the addition or attachment of an additional heteroatom. Synthesized compounds with an additional amine incorporated into either the 5- or 6-membered ring (5k–l) exhibited good to excellent activities (entries 12–13). However, the introduction of both amine and oxygen on 6-membered ring (5m) did not result in effective inhibition (entry 14). These results suggest that adding an amine to a ring structure could be valuable for discovering improved scaffolds.^30,31^ Therefore, we selected two scaffolds, 7*H*-pyrrolo[2,3-*d*]pyrimidine and 1*H*-pyrazolo[3,4-*b*]pyridine, for further validation of structure–activity relationships and drug-likeness.

Following scaffold optimization, the carboxamide part was also studied (Table 2). Compounds missing a benzyl group on the thiazole (6a and 6b) did not exhibit good activity (entries 1–2). Compounds with oxygen (6c and 6d) instead of sulfur (5k and 5l) were slightly less active (entries 3–4 in Table 2*versus* entries 12–13 in Table 1). Attachment of the carboxamide group at the 2-position (6e) resulted in a significant loss of activity (entry 5).

**Table 2:** Further optimized structures and the percent enzymatic activities

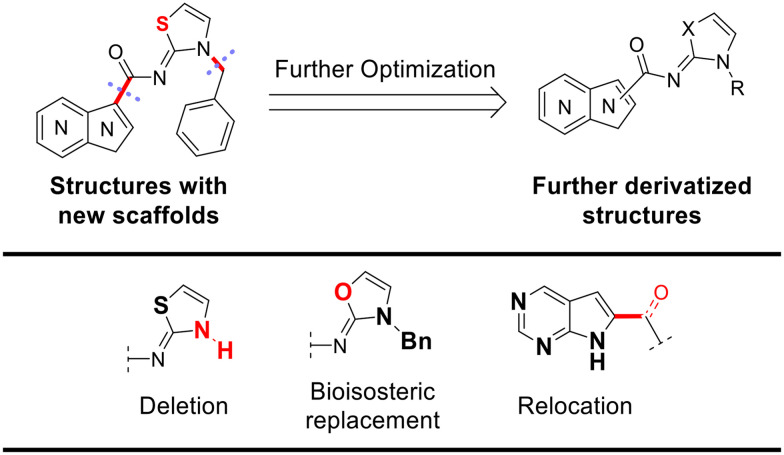						
Entry	Compound	Scaffold	R	X	% enzymatic activity (at 1 μM of a compound)	
					LATS1(h)	LATS2(h)
1	6a	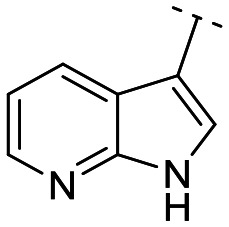	H	S	82 ± 4	78 ± 4
2	6b	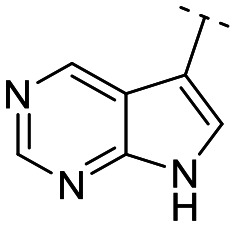	H	S	100 ± 4	103 ± 4
3	6c		Bn	O	52 ± 2	61 ± 2
4	6d	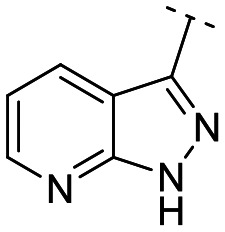	Bn	O	30 ± 3	28 ± 3
5	6e	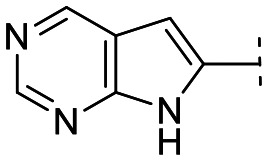	Bn	S	96 ± 0	107 ± 2

We selected the two most active compounds to determine their IC_50_ values against LATS1 and LATS2. Pyrrolopyrimidine 5k exhibited nearly ten-fold lower activity, while pyrazolopyridine structure 5l demonstrated comparable results to the reference compound 1 (Table 3). Isoform selectivity was not significant in any of the cases.

**Table 3:** IC_50_ values of selected compounds

Compound	Kinase	IC_50_ (nM)
1 (Truli)	LATS1(h)	22
	LATS2(h)	6
5k	LATS1(h)	265
	LATS2(h)	395
5l	LATS1(h)	43
	LATS2(h)	24

**Fig. 4:**
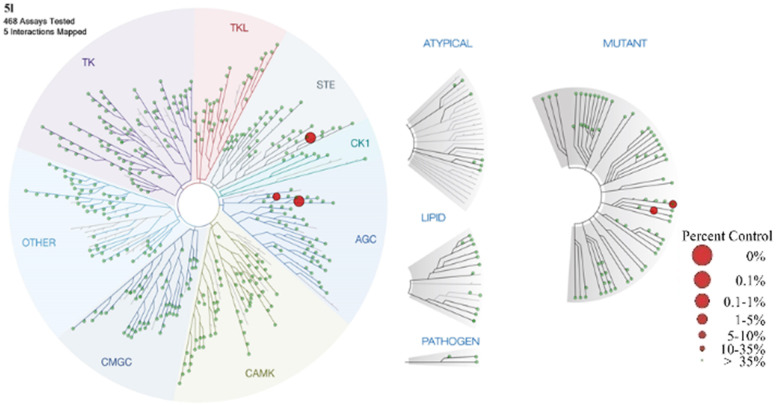
Kinome tree with 5l kinase selectivity data (data are generated using Discoverex Treespot). A large red circle indicates higher-affinity binding of numerous kinases.

The results of kinase profiling for the most potent compound, 5l, demonstrated exceptional selectivity towards other protein kinases in comparison to the reported LATS inhibitors. Apart from the intended targets, only three kinases (DMPK, YSK4 and FLT3(D835V)) showed activity out of 468 kinases tested at 100 nM of 5l (Fig. 4) resulting in an excellent kinase selectivity, S(35) score of 0.007. The selectivity value of 5l is comparable to the selectivity score of the recently reported LATS inhibitor, 2, NIBR-LTSi.^26^ The selectivity score for Truli has not been reported, but it has been noted that Truli exhibited better binding affinity for more than 30 protein kinases out of the 314 tested, compared to its affinity for LATS1 and LATS2.^23^ For another reported LATS inhibitor, 3, GA-017, it was noted that it exhibited high potency against 16 out of 321 tested protein kinases.^25^

The drug-likeness of the two selected compounds was assessed. Compared to reference compound 1, both compounds 5k and 5l showed improved thermodynamic water solubility. According to the AI-predicted metabolic stability values obtained with using PredMS (please refer to Fig. 3), compounds 5k (HLM: stable (0.592)) and 5l (HLM: stable (0.561)) exhibited higher stability in human liver microsomes than 1, Truli (HLM: unstable (0.457)) did. In case of experimentally obtained metabolic stability values (Table 4), both compounds (5k and 5l) exhibited more than ten times higher stability in human liver microsomes than 1, Truli, consistent with the AI-predicted metabolic stabilities. It was evident that adding an amine to the ring positively influenced the compounds' drug-likeness,^30,31,38^ potentially making them more suitable for *in vivo* studies.

**Table 4:** Metabolic stability and solubility of 1, 5k, and 5l

Compound	1 (Truli)	5k	5l
MLM phase I stability (% remaining after 30 min)	0.18 ± 0.03	14 ± 0.6	0.69 ± 0.17
HLM phase I stability (% remaining after 30 min)	2.7 ± 0.5	43 ± 1.1	35 ± 1.3
Solubility (mg mL^−1^)	0.14 ± 0.026	0.68 ± 0.089	0.29 ± 0.015

**Fig. 5:**
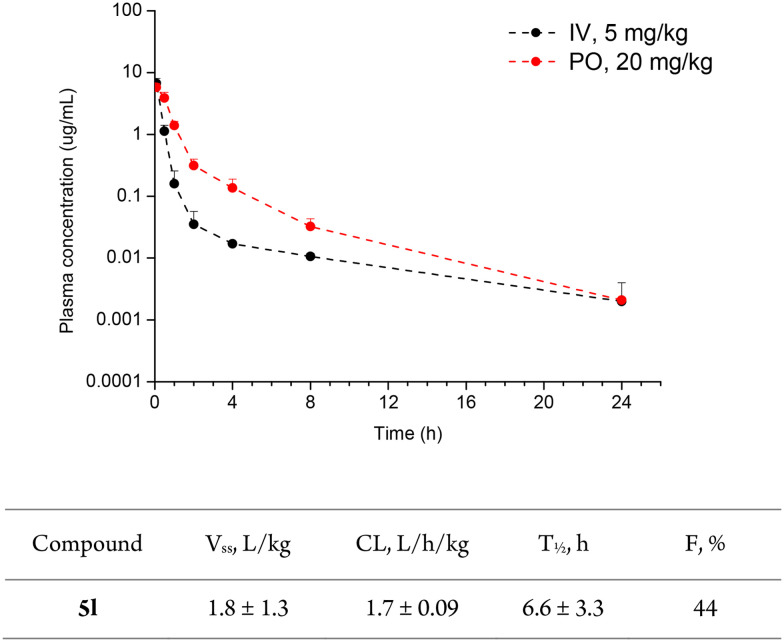
Pharmacokinetic profile of 5l obtained using male mice.

*In vivo* pharmacokinetic profiles of 5k and 5l were tested using male mice. Both compounds demonstrated high bioavailability upon oral administration and excellent pharmacokinetic (PK) profile. Pharmacokinetic data on compound 5k can be found in ESI† (Fig. S42, Table S5). The reported PK data for both compounds was comparable to the recently reported LATS inhibitor, 2, NIBR-LTSi.^26^ Other reported LATS inhibitors, including 1, Truli, and 3, GA-017, did not provide information on their PK profiles. The selected PK parameters for 5l including volume of distribution (*V*_ss_), clearance rate (CL), half-life (*T*_½_) and bioavailability (*F*) are depicted in Fig. 5.

## 3. Conclusion

We have developed novel selective LATS inhibitors with high oral bioavailability. Using docking studies and AI-assisted prediction of metabolic stability, we identified alternative scaffolds that substantially improved drug-likeness. Interestingly, these modifications also increased selectivity towards other kinases. Biological studies on these compounds for potential applications in regenerative medicine are currently underway, specifically focusing on their ability to modulate the Hippo pathway for treating regenerative diseases. Detailed results from these studies will be reported in due course.

## 4. Experimental part

### 4.1. Chemistry

For more detailed information see ESI.†

#### 4.1.1. General

Commercially available reactants and solvents were used without additional purification. Analytical thin layer chromatography (TLC) was performed on Kieselgel 60 F_254_ glass plates precoated with a 0.2 mm thickness of silica gel. The TLC plates were visualized by shortwave (254 nm). Medium-pressure liquid chromatography (MPLC) was performed on CombiFlash NextGen 300+ apparatus using Buchi FlashPure EcoFlex silica cartridges with 50 μm particle size. Preparatory TLC was performed on Kieselgel 60 F_254_ glass plates precoated with a 1.0 mm thickness of silica gel. ^1^H NMR spectra were obtained at 300 MHz, 400 MHz or 500 MHz (Bruker). ^13^C NMR spectra were acquired at 101 MHz and 125 MHz (Bruker). Liquid-chromatography mass spectrometry (LCMS) with an electrospray ionization (ESI) method was used to obtain mass spectra. High-resolution mass spectra (HRMS) were recorded with an electron impact ionization (EI) using a sector field mass analyzer. The melting points were determined in capillary tubes on digital melting point apparatus electrothermal IA9300. Compound purity was measured using a Shimadzu Nexera lite HPLC system. Data acquisition and processing were performed using LabSolutions software.

The compounds 4a–b, 5a–m, 6a–e were synthesized according to the reported procedure.^1,2^

#### 4.1.2. 3-Benzylthiazol-2(3*H*)-imine (4a)

Benzyl bromide (1.1 eq., 32.95 mmol, 5.6 g) was added to a mixture containing 2-aminothiazole (1.0 eq., 29.95 mmol, 3.0 g) dissolved in 21 mL of *N*,*N*-dimethylformamide (DMF). The resulting reaction mixture was heated at 50 °C for 18 h. After cooling, the reaction was concentrated under reduced pressure, the residue was dissolved in ethyl acetate (EA) and diluted with aqueous 10 M NaOH at 0 °C. The reaction mixture was stirred for additional 1 h. Following that, the reaction mixture was extracted with EA and water, the organic layer was collected and washed with a saturated aqueous NaCl solution (brine). Subsequently, the organic phase was dried using anhydrous Na_2_SO_4_, and the solvent was evaporated under reduced pressure. Afterwards, the residue obtained was purified by MPLC (methanol : dichloromethane (DCM) = 10 : 90), providing 4a as yellow oil.

Yield: 67%; ^1^H NMR (300 MHz, CDCl_3_) *δ* 7.40–7.27 (m, 5H), 6.35 (d, *J* = 5.0 Hz, 1H), 5.79 (d, *J* = 5.0 Hz, 1H), 4.91 (s, 2H); ^13^C NMR (101 MHz, CDCl_3_) *δ* 164.89, 136.71, 128.97, 127.93, 127.85, 127.08, 97.96, 49.11; LCMS (ESI) *m*/*z*: 191.00 [M + H]^+^.

#### 4.1.3. 3-Benzyloxazol-2(3*H*)-imine (4b)

To a solution of 2-aminooxazole (1.0 eq., 1.1893 mmol, 100 mg) dissolved in 5 mL of acetone was added benzyl bromide (1.1 eq., 1.3082 mmol, 223 mg). The reaction mixture was heated at 60 °C for 6 h. After cooling, the reaction was concentrated under reduced pressure, the residue obtained was dried under high vacuum pump providing 4b as a yellow oil. The crude product was used further without purification.

#### 4.1.4. General procedure for the synthesis of compounds 5a–f, 5k–m and 6c–e

To a solution containing carboxylic acid (0.8 eq.) dissolved in DMF was added HATU (1.5 eq.) at 0 °C. After the reaction was stirred for 30 min, DIPEA (3.0 eq.) and imine (1.0 eq.) were added to the reaction mixture. The reaction temperature was increased to rt and stirred for overnight. After the reaction was completed, it was quenched by addition of water. In case solid formation was observed, it was filtered out and dried. Other than that, the reaction mixture was extracted with EA, the organic layer was collected and washed with a brine. Subsequently, the organic phase was dried using anhydrous Na_2_SO_4_, and the solvent was evaporated under reduced pressure. The product was further purified by MPLC (methanol : DCM = 10 : 90) or by preparatory TLC (methanol : DCM = 5 : 95), providing compounds 5a–f, 5k–m and 6c–e.

##### 4.1.4.1. (*Z*)-*N*-(3-Benzylthiazol-2(3*H*)-ylidene)benzamide (5a)

Yield: 56%; m.p.: 68–69 °C; HPLC: 95.9% (*t*_R_ = 10.0 min); ^1^H NMR (300 MHz, CDCl_3_) *δ* 8.40–8.33 (m, 2H), 7.51–7.41 (m, 3H), 7.39–7.31 (m, 5H), 6.96 (d, *J* = 4.8 Hz, 1H), 6.65 (d, *J* = 4.7 Hz, 1H), 5.50 (s, 2H); ^13^C NMR (101 MHz, CDCl_3_) *δ* 174.29, 168.28, 137.04, 135.73, 133.60, 131.61, 130.26, 129.44, 129.20, 128.58, 128.31, 128.18, 125.72, 109.54, 52.12; LC–MS (ESI) *m*/*z*: 295.1 [M + H]^+^.

##### 4.1.4.2. (*Z*)-*N*-(3-Benzylthiazol-2(3*H*)-ylidene)nicotinamide (5b)

Yield: 56%; m.p.: 69–70 °C; HPLC: 99.0% (*t*_R_ = 2.7 min); ^1^H NMR (300 MHz, CDCl_3_) *δ* 9.54 (dd, *J* = 2.1, 0.9 Hz, 1H), 8.70 (dd, *J* = 4.8, 1.8 Hz, 1H), 8.54 (dt, *J* = 7.9, 2.1 Hz, 1H), 7.41–7.31 (m, 6H), 7.02 (d, *J* = 4.7 Hz, 1H), 6.72 (d, *J* = 4.7 Hz, 1H), 5.51 (s, 2H); ^13^C NMR (101 MHz, CDCl_3_) *δ* 172.58, 168.27, 151.88, 151.08, 136.85, 135.38, 132.50, 129.25, 128.69, 128.23, 126.06, 123.28, 109.95, 52.30; LC–MS (ESI) *m*/*z*: 296.10 [M + H]^+^.

##### 4.1.4.3. (*Z*)-*N*-(3-Benzylthiazol-2(3*H*)-ylidene)-1*H*-indole-3-carboxamide (5c)

Yield: 27%; m.p.: 209–210 °C; HPLC: 99.6% (*t*_R_ = 14.9 min); ^1^H NMR (400 MHz, CDCl_3_) *δ* 9.08 (s, 1H), 8.58–8.49 (m, 1H), 8.08 (d, *J* = 2.9 Hz, 1H), 7.42–7.30 (m, 6H), 7.24–7.16 (m, 2H), 6.87 (d, *J* = 4.8 Hz, 1H), 6.55 (d, *J* = 4.8 Hz, 1H), 5.48 (s, 2H); ^13^C NMR (101 MHz, CDCl_3_) *δ* 173.07, 166.91, 136.88, 135.88, 131.37, 129.11, 128.38, 128.16, 126.38, 125.44, 122.54, 122.22, 121.39, 116.06, 111.77, 108.85, 51.91; LC–MS (ESI) *m*/*z*: 334.00 [M + H]^+^.

##### 4.1.4.4. (*Z*)-*N*-(3-Benzylthiazol-2(3*H*)-ylidene)-1-methyl-1*H*-indole-3-carboxamide (5d)

Yield: 9%; m.p.: 184–185 °C; HPLC: 99.3% (*t*_R_ = 17.3 min); ^1^H NMR (400 MHz, CDCl_3_) *δ* 8.52 (d, *J* = 7.1 Hz, 1H), 7.96 (s, 1H), 7.40–7.31 (m, 5H), 7.30–7.20 (m, 3H), 6.88 (d, *J* = 4.8 Hz, 1H), 6.57 (d, *J* = 4.8 Hz, 1H), 5.51 (s, 2H), 3.84 (s, 3H); ^13^C NMR (125 MHz, CDCl_3_) *δ* 172.67, 166.88, 137.73, 136.08, 135.16, 129.19, 128.46, 128.22, 127.25, 125.25, 122.68, 122.36, 121.40, 114.90, 109.58, 108.82, 51.92, 33.47; LC–MS (ESI) *m*/*z*: 348.1 [M + H]^+^.

##### 4.1.4.5. (*Z*)-*N*-(3-Benzylthiazol-2(3*H*)-ylidene)-1*H*-pyrrolo[2,3-c]pyridine-3-carboxamide (5e)

Yield: 29%; m.p.: 149–150 °C; HPLC: 98.2% (*t*_R_ = 4.5 min); ^1^H NMR (500 MHz, MeOD-*d*_4_) *δ* 8.68 (s, 1H), 8.25 (s, 1H), 8.23 (d, *J* = 5.6 Hz, 1H), 8.09 (d, *J* = 5.6 Hz, 1H), 7.39–7.26 (m, 5H), 7.24 (d, *J* = 4.6 Hz, 1H), 6.83 (d, *J* = 4.6 Hz, 1H), 5.50 (s, 2H); ^13^C NMR (101 MHz, MeOD-*d*_4_) *δ* 173.27, 168.70, 138.20, 137.69, 137.63, 135.28, 134.12, 133.80, 129.95, 129.17, 128.78, 128.22, 118.13, 116.82, 110.51, 52.87; LC–MS (ESI) *m*/*z*: 335.00 [M + H]^+^.

##### 4.1.4.6. (*Z*)-*N*-(3-Benzylthiazol-2(3*H*)-ylidene)-1*H*-pyrrolo[3,2-c]pyridine-3-carboxamide (5f)

Yield: 41%; HPLC: 98.8% (*t*_R_ = 4.7 min); ^1^H NMR (400 MHz, DMSO-*d*_6_) *δ* 9.52 (s, 1H), 8.28 (d, *J* = 5.8 Hz, 1H), 8.25 (s, 1H), 7.62 (d, *J* = 4.7 Hz, 1H), 7.53 (d, *J* = 5.8 Hz, 1H), 7.43 (d, *J* = 7.1 Hz, 2H), 7.36 (t, *J* = 7.4 Hz, 2H), 7.29 (t, *J* = 7.3 Hz, 1H), 7.01 (d, *J* = 4.7 Hz, 1H), 5.55 (s, 2H); ^13^C NMR (101 MHz, DMSO-*d*_6_) *δ* 170.43, 166.05, 143.04, 140.66, 139.45, 136.73, 132.95, 128.74, 127.84, 127.79, 127.31, 122.66, 115.31, 108.79, 107.78, 50.94; LC–MS (ESI) *m*/*z*: 335.00 [M + H]^+^.

##### 4.1.4.7. (*Z*)-*N*-(3-Benzylthiazol-2(3*H*)-ylidene)-7*H*-pyrrolo[2,3-*d*]pyrimidine-5-carboxamide (5k)

Yield: 76%; m.p.: 224–225 °C; HPLC: 99.2% (*t*_R_ = 3.1 min); ^1^H NMR (300 MHz, CDCl_3_) *δ* 10.22 (s, 1H), 9.73 (s, 1H), 8.94 (s, 1H), 8.18 (s, 1H), 7.32–7.40 (m, 5H), 6.97 (d, *J* = 4.7 Hz, 1H), 6.68 (d, *J* = 4.7 Hz, 1H), 5.53 (s, 2H); ^13^C NMR (101 MHz, CDCl_3_ + MeOD-*d*_4_) *δ* 171.19, 167.55, 151.85, 151.00, 150.91, 135.22, 132.07, 129.03, 128.37, 127.65, 126.09, 117.29, 114.90, 109.66, 51.99; HRMS (EI) calcd. for C_17_H_13_N_5_OS *m*/*z*: 335.0841, found *m*/*z*: 335.0837 [M]^+^.

##### 4.1.4.8. (*Z*)-*N*-(3-Benzylthiazol-2(3*H*)-ylidene)-1*H*-pyrazolo[3,4-*b*]pyridine-3-carboxamide (5l)

Yield: 28%; m.p.: 244–245 °C; HPLC: 98.8% (*t*_R_ = 9.5 min); ^1^H NMR (300 MHz, CDCl_3_) *δ* 12.13 (s, 1H), 8.79 (dd, *J* = 8.1, 1.6 Hz, 1H), 8.62 (dd, *J* = 4.6, 1.6 Hz, 1H), 7.33–7.37 (m, 5H), 7.25 (tr, *J* = 6 Hz, 1H), 6.98 (d, *J* = 4.7 Hz, 1H), 6.74 (d, *J* = 4.7 Hz, 1H), 5.60 (s, 2H); ^13^C NMR (101 MHz, CDCl_3_ + MeOD-*d*_4_) *δ* 169.58, 168.24, 152.28, 148.84, 135.24, 132.92, 129.12, 128.54, 128.07, 126.22, 118.32, 115.09, 110.28, 52.24; HRMS (EI) calcd. for C_17_H_13_N_5_OS *m*/*z*: 335.0841, found *m*/*z*: 335.0837 [M]^+^.

##### 4.1.4.9. (*Z*)-2-Amino-*N*-(3-benzylthiazol-2(3*H*)-ylidene)-4-oxo-4,7-dihydro-3*H*-pyrrolo[2,3-*d*]pyrimidine-5-carboxamide (5m)

Yield: 7%; HPLC: 95.0% (*t*_R_ = 1.5 min); ^1^H NMR (300 MHz, MeOD-*d*_4_) *δ* 7.66 (s, 1H), 7.34 (s, 6H), 6.61 (s, 1H), 5.42 (s, 2H); ^13^C NMR (101 MHz, MeOD-*d*_4_) *δ* 191.11, 165.76, 147.19, 135.96, 129.16, 128.63, 128.53, 127.75, 126.85, 126.19, 119.49, 106.63, 39.38; LC–MS (ESI) *m*/*z*: 367.37 [M + H]^+^.

##### 4.1.4.10. (*Z*)-*N*-(3-Benzyloxazol-2(3*H*)-ylidene)-7*H*-pyrrolo[2,3-*d*]pyrimidine-5-carboxamide (6c)

Yield: 38%; m.p.: 210–211 °C; HPLC: 95.4% (*t*_R_ = 1.3 min); ^1^H NMR (400 MHz, DMSO-*d*_6_) *δ* 12.52 (s, 1H), 9.39 (s, 1H), 8.79 (s, 1H), 8.13 (d, *J* = 2.5 Hz, 1H), 7.72 (d, *J* = 1.7 Hz, 1H), 7.54 (d, *J* = 1.7 Hz, 1H), 7.45–7.29 (m, 5H), 5.13 (s, 2H); ^13^C NMR (101 MHz, DMSO-*d*_6_) *δ* 168.39, 156.38, 151.95, 151.42, 150.21, 136.00, 132.19, 132.07, 128.80, 128.00, 127.74, 117.69, 116.76, 114.81, 47.98; LC–MS (ESI) *m*/*z*: 320.10 [M + H]^+^.

##### 4.1.4.11. (*Z*)-*N*-(3-Benzyloxazol-2(3*H*)-ylidene)-1*H*-pyrazolo[3,4-*b*]pyridine-3-carboxamide (6d)

Yield: 16%; m.p.: 170–171 °C; HPLC: 99.7% (*t*_R_ = 4.5 min); ^1^H NMR (400 MHz, DMSO-*d*_6_) *δ* 8.52 (d, *J* = 4.4 Hz, 1H), 8.48 (dd, *J* = 8.1, 1.6 Hz, 1H), 7.80 (d, *J* = 1.7 Hz, 1H), 7.61 (d, *J* = 1.7 Hz, 1H), 7.45–7.30 (m, 5H), 7.21 (dd, *J* = 8.1, 4.5 Hz, 1H), 5.16 (s, 2H); ^13^C NMR (101 MHz, DMSO-*d*_6_) *δ* 166.67, 157.09, 152.34, 148.67, 141.86, 135.80, 132.55, 131.83, 128.83, 128.03, 127.61, 118.07, 118.00, 114.04, 48.17; LC–MS (ESI) *m*/*z*: 320.00 [M + H]^+^.

##### 4.1.4.12. (*Z*)-*N*-(3-Benzylthiazol-2(3*H*)-ylidene)-7*H*-pyrrolo[2,3-*d*]pyrimidine-6-carboxamide (6e)

Yield: 46%; m.p.: 249–250 °C; HPLC: 95.0% (*t*_R_ = 4.3 min); ^1^H NMR (300 MHz, DMSO-*d*_6_) *δ* 12.67 (s, 1H), 9.12 (s, 1H), 8.84 (s, 1H), 7.70 (d, *J* = 4.6 Hz, 1H), 7.47 (d, *J* = 7.0 Hz, 2H), 7.42–7.24 (m, 5H), 7.14 (d, *J* = 4.6 Hz, 1H), 5.66 (s, 2H); ^13^C NMR (101 MHz, DMSO-*d*_6_) *δ* 166.81, 166.51, 152.71, 151.38, 151.26, 137.08, 136.68, 128.71, 128.29, 128.23, 127.94, 127.83, 118.50, 110.04, 103.15, 50.93; LC–MS (ESI) *m*/*z*: 336.37 [M + H]^+^.

#### 4.1.5. General procedure for the synthesis of compounds 6a–b

To a solution containing carboxylic acid (1.0 eq.) dissolved in pyridine (40 eq.) was added (HBTU) (2.0 eq.) at 0 °C. After the reaction was stirred for 30 min, 2-aminothiazole (1.5 eq.) was added to the reaction mixture. The reaction temperature was gradually increased to 80 °C and stirred for 16 h. The reaction temperature was increased to 100 °C and the reaction was stirred for an additional 5 h. After cooling down, the reaction was quenched by ice-cold water. The solid formed was filtered out, washed with water and acetonitrile, then dried, providing 6a–b.

##### 4.1.5.1. (*Z*)-*N*-(Thiazol-2(3*H*)-ylidene)-1*H*-pyrrolo[2,3-*b*]pyridine-3-carboxamide (6a)

Yield: 56%; m.p.: 232–233 °C; HPLC: 97.1% (*t*_R_ = 2.4 min); ^1^H NMR (300 MHz, DMSO-*d*_6_) *δ* 12.45 (s, 1H), 12.27 (s, 1H), 8.68 (d, *J* = 2.7 Hz, 1H), 8.54 (dd, *J* = 7.9, 1.7 Hz, 1H), 8.34 (dd, *J* = 4.7, 1.7 Hz, 1H), 7.52 (d, *J* = 3.6 Hz, 1H), 7.25 (dd, *J* = 7.9, 4.7 Hz, 1H), 7.21 (d, *J* = 3.6 Hz, 1H); ^13^C NMR (101 MHz, DMSO-*d*_6_) *δ* 161.80, 158.67, 148.62, 144.12, 137.48, 130.59, 129.36, 118.76, 117.64, 113.03, 107.08; LC–MS (ESI) *m*/*z*: 245.00 [M + H]^+^.

##### 4.1.5.2. (*Z*)-*N*-(Thiazol-2(3*H*)-ylidene)-7*H*-pyrrolo[2,3-*d*]pyrimidine-5-carboxamide (6b)

Yield: 69%; m.p.: 302–303 °C; HPLC: 99.3% (*t*_R_ = 0.98 min); ^1^H NMR (400 MHz, DMSO-*d*_6_) *δ* 12.86 (s, 1H), 12.46 (s, 1H), 9.47 (s, 1H), 8.89 (s, 1H), 8.71 (d, *J* = 2.6 Hz, 1H), 7.54 (d, *J* = 3.5 Hz, 1H), 7.25 (d, *J* = 3.5 Hz, 1H); ^13^C NMR (101 MHz, DMSO-*d*_6_) *δ* 161.03, 158.45, 152.25, 151.88, 150.18, 137.57, 131.41, 117.04, 113.36, 107.63; LC–MS (ESI) *m*/*z*: 246.00 [M + H]^+^.

#### 4.1.6. Procedure for the synthesis of (*Z*)-1-benzyl-*N*-(3-benzylthiazol-2(3*H*)-ylidene)-1*H*-pyrrolo[2,3-*b*]pyridine-3-carboxamide (5i)

Step 1. (Z)-*N*-(3-benzylthiazol-2(3*H*)-ylidene)-1*H*-pyrrolo[2,3-*b*]pyridine-3-carboxamide 1 (Truli) was synthesized according to the reported procedure.^36^ All the analytical data were compared and matched with the reference compound. Step 2. A compound 1 (1.0 eq., 0.0598 mmol, 20 mg) obtained in the abovementioned step was dissolved in 1 mL of DMF, and sodium hydride (60% in mineral oil) (1.5 eq., 3.6 mg) was added to the resulting solution at 0 °C. After 10 min, benzyl bromide (1.2 eq., 0.0718 mmol, 12 mg) was added to the reaction mixture. The reaction was stirred at rt for 1.5 h. After cooling, the reaction was quenched by slowly addition of saturated aqueous ammonium chloride solution. The reaction was extracted with EA and water, the organic layer was collected and washed with brine. Afterwards, the organic layer was dried using anhydrous Na_2_SO_4_, and the solvent was evaporated under reduced pressure. Afterwards, the residue obtained was purified by MPLC (methanol : DCM = 10 : 90), providing 5i as a white solid.

Yield: 61%; m.p.: 95–96 °C; HPLC: 98.1% (*t*_R_ = 17.7 min); ^1^H NMR (400 MHz, CDCl_3_) *δ* 8.68 (dd, *J* = 7.9, 1.6 Hz, 1H), 8.34 (dd, *J* = 4.7, 1.6 Hz, 1H), 8.03 (s, 1H), 7.35–7.20 (m, 10H), 7.15 (dd, *J* = 7.9, 4.7 Hz, 2H), 6.87 (d, *J* = 4.8 Hz, 1H), 6.56 (d, *J* = 4.8 Hz, 1H), 5.50 (s, 2H), 5.44 (s, 2H); ^13^C NMR (101 MHz, CDCl_3_) *δ* 172.09, 167.13, 148.43, 143.70, 137.08, 135.80, 133.60, 130.84, 129.19, 128.93, 128.51, 128.12, 127.97, 127.85, 125.46, 119.67, 117.75, 114.00, 109.08, 52.02, 48.34; LC–MS (ESI) *m*/*z*: 425.3 [M + H]^+^.

#### 4.1.7. General procedure for the synthesis of compounds 5g–h and 5j

A corresponding carboxamide (1.0 eq.) was dissolved in DMF, and benzyl bromide (1.2 eq.) was added to the resulting solution. The reaction mixture was heated to 60 °C and stirred for overnight. After cooling down, the reaction mixture was diluted with EA and aqueous 10 M NaOH at 0 °C. The reaction mixture was stirred for an additional 1 h. If the formation of solid was observed, the solid was filtered out, washed with water and dried. If the formation of the solid was not observed, the reaction mixture was extracted with EA and water, the organic layer was collected, washed with brine and dried using anhydrous Na_2_SO_4_. The solvent was removed using rotary evaporator and dried under high vacuum. The product was purified by MPLC (methanol : DCM = 10 : 90), providing corresponding compounds 5g–h and 5j.

##### 4.1.7.1. (*Z*)-6-Benzyl-*N*-(3-benzylthiazol-2(3*H*)-ylidene)-6*H*-pyrrolo[2,3-*c*]pyridine-3-carboxamide (5g)

Yield: 9%; HPLC: 97.2% (*t*_R_ = 10.4 min); ^1^H NMR (400 MHz, CDCl_3_) *δ* 9.33 (s, 1H), 8.73 (d, *J* = 6.7 Hz, 1H), 8.66 (s, 1H), 8.10 (s, 1H), 7.95 (d, *J* = 6.7 Hz, 1H), 7.50–7.27 (m, 10H), 7.02 (d, *J* = 4.7 Hz, 1H), 6.72 (d, *J* = 4.7 Hz, 1H), 5.61 (s, 2H), 5.47 (s, 2H); ^13^C NMR (101 MHz, CDCl_3_) *δ* 170.85, 169.85, 142.60, 135.82, 135.18, 132.58, 131.42, 130.44, 130.09, 129.64, 129.37, 128.80, 128.74, 127.98, 126.22, 119.85, 118.27, 109.76, 65.00, 52.36; LC–MS (ESI) *m*/*z*: 425.20 [M + H]^+^.

##### 4.1.7.2. (*Z*)-5-Benzyl-*N*-(3-benzylthiazol-2(3*H*)-ylidene)-5*H*-pyrrolo[3,2-*c*]pyridine-3-carboxamide (5h)

Yield: 64%; m.p.: 189–190 °C; HPLC: 99.0% (*t*_R_ = 10.1 min); ^1^H NMR (300 MHz, CDCl_3_) *δ* 9.23 (s, 1H), 8.86 (s, 1H), 7.69 (d, *J* = 6.8 Hz, 1H), 7.52 (dd, *J* = 6.8, 1.9 Hz, 1H), 7.43–7.27 (m, 8H), 7.16 (dd, *J* = 6.8, 2.9 Hz, 2H), 6.90 (d, *J* = 4.8 Hz, 1H), 6.54 (d, *J* = 4.8 Hz, 1H), 5.44 (s, 2H), 5.37 (s, 2H); ^13^C NMR (101 MHz, CDCl_3_ + MeOD-*d*^4^) *δ* 172.20, 166.70, 135.75, 135.61, 134.54, 129.33, 129.21, 129.1, 129.03, 128.33, 127.86, 127.59, 125.70, 113.94, 108.75, 62.96, 51.79; LC–MS (ESI) *m*/*z*: 425.10 [M + H]^+^.

##### 4.1.7.3. (*Z*)-7-Benzyl-*N*-(3-benzylthiazol-2(3*H*)-ylidene)-7*H*-pyrrolo[2,3-*b*]pyridine-3-carboxamide (5j)

Yield: 63%; m.p.: 107–108 °C; HPLC: 96.5% (*t*_R_ = 10.4 min); ^1^H NMR (400 MHz, CDCl_3_) *δ* 8.87 (dd, *J* = 7.5, 1.2 Hz, 1H), 8.75 (s, 1H), 7.59 (dd, *J* = 6.3, 1.2 Hz, 1H), 7.38–7.27 (m, 10H), 6.95 (dd, *J* = 7.5, 6.3 Hz, 1H), 6.87 (d, *J* = 4.8 Hz, 1H), 6.50 (d, *J* = 4.8 Hz, 1H), 5.88 (s, 2H), 5.44 (s, 2H); ^13^C NMR (101 MHz, CDCl_3_) *δ* 172.42, 166.39, 151.60, 150.81, 136.03, 134.82, 133.59, 129.56, 129.30, 129.15, 129.03, 128.80, 128.51, 128.28, 128.16, 125.33, 115.80, 112.22, 108.37, 55.72, 51.86; LC–MS (ESI) *m*/*z*: 425.00 [M + H]^+^.

### 4.2. Molecular docking simulation and AI-prediction of metabolic stability

The molecular docking was performed on the HyperLab (https://www.hyperlab.hits.ai/en) online platform with the homology model created from the crystal structure of kinase ROCK1 bound to azaindole thiazole inhibitor^34^ as there is no known crystal structure of the LATS kinases. The X-ray crystal structure of ROCK1 kinase complex (PDB ID: 5KKS) was downloaded from the protein data bank (https://www.rcsb.org). The 2D structure of the analyzed compounds 1, 5a–m and 6a–e were drawn using ChemDraw software. Binding energies were automatically calculated after registering the molecular structures of the analyzed compounds.^32,33^

KRICT-AI (pre-trained machine learning model, PredMS) platform was accessed online *via*https://predms.netlify.app/ and used to get predicted values of metabolic stabilities^34^ of the analyzed compounds 1, 5a–m and 6a–e. PredMS predicts metabolic stability for a given compound as stable (≥50% remaining at 30 min) or unstable (<50% remaining at 30 min) in human liver microsomes. The chemical structures of the compounds were presented in the simplified molecular-input line-entry system (SMILES) format and were further submitted for evaluation.

### 4.3. *In vitro* kinase activity assay

The Eurofins Kinase Profiler service was requested and used for obtaining *in vitro* kinase activity data. Kinase activity was determined at 1 μM compound concentration and at 90 μM ATP concentration for LATS1(h) and at 155 μM for LATS2(h) kinases for compounds 1, 5a–m and 6a–e. For LATS1(h) and LATS2(h), the selected kinase was incubated with 8 mM MOPS (pH 7.0), 0.2 mM EDTA, 250 μM RLGRDKYKTLRQIRQ, 10 mM magnesium acetate, and [γ-33P-ATP]. The reaction was initiated by adding the Mg/ATP mixture. After 40 minutes of incubation at room temperature, the reaction was halted by adding phosphoric acid to a final concentration of 0.5%. A 10 μL of the reaction mixture were then spotted onto a P30 filtermat, washed four times for 4 minutes each in 0.425% phosphoric acid, and one time in methanol before drying and scintillation counting. The values represent the average of two independent experiments. The IC_50_ data for compounds 1, 5k, and 5l was also determined using the Eurofins IC_50_ Profiler service at 90 μM ATP concentration for LATS1(h) and at 155 μM for LATS2(h) kinases. The IC_50_ curves (refer to Fig. S39–S41†) were generated using 9 test compound concentrations, diluted in half-log increments from 10 μM to 1 nM, alongside vehicle control wells. The values represent the mean of two independent experiments.

### 4.4. Kinase profiling

A set of 468 kinase inhibitory tests was performed on the compound 5l at a concentration of 100 nM by using the scanMAX kinase assay panel of KINOME*scan*. In this study, the kinase binding maps of 5l demonstrated the strength and relative specificity of kinase-binding interactions. TREE*spot* interaction map for compound 5l was generated online using the TREE*spot*™ software tool.

### 4.5. Experimental determination of metabolic stability and thermodynamic solubility

Microsomes diluted with potassium phosphate buffer were incubated at 37 °C for 5 minutes, then the tested compound and NADPH were added and reacted at 37 °C for 30 minutes (test compound final conc.: 1 μM, microsome final conc.: 0.5 mg mL^−1^). To terminate the reaction, cold acetonitrile containing an internal standard was added and then treated with deproteinization. After centrifugation (4000 rpm, 4 °C, 15 min), the supernatant was analyzed by LC–MS/MS (mass spectrometry (Agilent 6460) with HPLC (Agilent 1260)).^39,40^ To measure solubility, 3 mg of each compound was weighed into 1.5 mL Eppi tubes. 700 μL of D_2_O (deuterium oxide) was then added and the sample was sonicated for 5 min and was subjected to shaking on a high-speed vibrating mixer for 24 h at rt and was centrifuged at 10 000 rpm for 5 min. For analysis, the supernatant was filtered using 0.45 μm PVDF syringe filter. For the quantitation, DMSO (dimethyl sulfoxide) was used as an internal standard. The concentration of each compound in D_2_O was calculated based on the integration ratio of the compound signal to the internal standard DMSO signal^41^ (2.71 ppm, 6H) (*n* = 3).

### 4.6. *In vivo* pharmacokinetics

Pharmacokinetic profiles of 5k and 5l were obtained using male mice. Blood was centrifuged to separate plasma, and 9× the volume of cold acetonitrile containing an internal standard is added, followed by deproteinization. After centrifugation (13 000 rpm, 4 °C, 10 min), the supernatant was analyzed by LC–MS/MS (mass spectrometry (Agilent 6460) with HPLC (Agilent 1260)).^42^ For the calibration curve of the compound, a 10× higher concentration solution (0.5–8000 ng mL^−1^) was prepared by adding it to blank plasma, and it was further prepared in the same manner as the tested compounds. All animal procedures were performed in accordance with the Guidelines for Care and Use of Laboratory Animals of Korea Research Institute of Chemical Technology and approved by the Animal Ethics Committee of Korea Research Institute of Chemical Technology.

## Data availability

All data generated or analysed during this study are included in this published article [and its ESI† files].

## Author contributions

Guldana Issabayeva: formal analysis, investigation, writing (original draft). On-Yu Kang: formal analysis, investigation, methodology. Seong Yun Choi: formal analysis, investigation, methodology. Ji Young Hyun: investigation, writing (review and editing). Seong Jun Park: investigation, writing (review and editing). Hei-Chul Jeung: funding acquisition, investigation, writing (review and editing). Hwan Jung Lim: funding acquisition, investigation, supervision, writing (original draft, review and editing). G. I. and O.-Y. K. contributed equally.

## Conflicts of interest

The authors declare that they have no known competing financial interests. The content and writing are solely responsible to the authors.

## Supplementary Material

MD-OLF-D4MD00492B-s001
