# Supplementary material for: Discovery of selective LATS inhibitors via scaffold hopping: enhancing drug-likeness and kinase selectivity for potential applications in regenerative medicine
Source: RSC Med Chem. 2024 Sep 14;15(12):4080–9. doi: 10.1039/d4md00492b (PMC11428031; doi:10.1039/d4md00492b)
Supplement: MD-015-D4MD00492B-s001 [file MD-015-D4MD00492B-s001.pdf]

Supplementary Information (SI) for RSC Medicinal Chemistry.
This journal is © The Royal Society of Chemistry 2024

Electronic Supplementary Information

Discovery of Selective LATS Inhibitors via Scaffold Hopping: Enhancing Drug-Likeness and Kinase Selectivity for Potential Applications in Regenerative Medicine

Guldana Issabayeva[a,b], On-Yu Kang[a], Seong Yun Choi[a,b], Ji Young Hyun[a,b], Seong Jun Park[a,b,*] Hei-Chul Jeung[c,*] Hwan Jung Lim[a,b,*]

[a] Data Convergence Drug Research Center, Korea Research Institute of Chemical Technology, 141 Gajeong-ro, Daejeon 34114, Republic of Korea

[b] Department of Medicinal Chemistry and Pharmacology, University of Science & Technology, 217 Gajeong-ro, Daejeon 34113, Republic of Korea

[c] Department of Medical Oncology, Yonsei University College of Medicine, 211 Eonju-ro, Gangnam-gu, Seoul 06273, Republic of Korea

*E-mail addresses: *indium@krict.re.kr*

TABLE OF CONTENTS

1. **Experimental section and synthesis**
2. **^{1}H NMR and ^{13}C NMR spectra**
3. **In vitro kinase activity assay**
4. **Solubility**
5. **Experimentally obtained metabolic stability**
6. **Mouse pharmacokinetics**
7. **Kinase screening results**
8. **KRICT-AI assisted prediction of metabolic stability**
9. **Molecular docking simulation**
10. **References**

1. Experimental section and synthesis

Commercially available reactants and solvents were purchased from commercial suppliers and used without additional purification. Analytical thin layer chromatography (TLC) was performed on Kieselgel 60 F_{254} glass plates precoated with a 0.2 mm thickness of silica gel. The TLC plates were visualized by shortwave (254 nm). Medium-pressure liquid chromatography (MPLC) was performed on CombiFlash NextGen 300+ apparatus using Buchi FlashPure EcoFlex silica cartridges with 50 µm particle size. Preparatory TLC was performed on Kieselgel 60 F_{254} glass plates precoated with a 1.0 mm thickness of silica gel. ^{1}H NMR spectra were obtained at 300 MHz, 400 MHz or 500 MHz Bruker using $CDCl_3$, MeOD-d_4 or DMSO-d_6 as a solvent. ^{1}H NMR assignment abbreviations are the following: singlet (s), doublet (d), triplet (t), quartet (q), doublet of doublets (dd), doublet of triplets (dt), and multiplet (m). ^{13}C NMR spectra were acquired at 101 MHz and 125 MHz Bruker using $CDCl_3$, MeOD-d_4 or DMSO-d_6 as a solvent and TMS as an internal standard. Liquid-chromatography mass spectrometry (LCMS) with an electrospray ionization (ESI) method was used to obtain mass spectra. High-resolution mass spectra (HRMS) were recorded with an electron impact ionization (EI) using a sector field mass analyzer. The melting points were determined in capillary tubes on digital melting point apparatus electrothermal IA9300. Compound purity was measured using a Shimadzu Nexera lite HPLC system. Data acquisition and processing were performed using LabSolutions software. The HPLC conditions included a Sepax Proteomix RP-1000 column (5 µm, 1000Å, 4.6 × 150 mm), a flow rate of 0.5 mL/min, UV detection at 220 nm and 280 nm, and a gradient of 100% water (0.1% trifluoroacetic acid) maintained from 0 to 5 min. A linear gradient from 100% water (0.1% trifluoroacetic acid) to 90% acetonitrile (0.1% trifluoroacetic acid) occurred from 5 to 17 min, returning to 100% water (0.1% trifluoroacetic acid) in 5 min and maintained for an additional 10 min (35 min).

The compounds **4a-b**, **5a-m**, **6a-e** were synthesized according to the reported procedure.[1,2]

1.1 Procedure for the synthesis of 3-benzylthiazol-2(3*H*)-imine **4a**

Benzyl bromide (1.1 eq., 32.95 mmol, 5.6 g) was added to a mixture containing 2-aminothiazole (1.0 eq., 29.95 mmol, 3.0 g) dissolved in 21 mL of *N*,*N*-dimethylformamide (DMF). The resulting reaction mixture was heated at 50 ºC for 18 h. After cooling, the reaction was concentrated under reduced pressure by rotary evaporator. The residue was dissolved in ethyl acetate (EA) and diluted with aqueous 10 M NaOH at 0 ºC. The reaction mixture was

stirred for additional 1 h. Following that, the reaction mixture was extracted with EA and water, the organic layer was collected and washed with a saturated aqueous NaCl solution (brine). Subsequently, the organic phase was dried using anhydrous Na_2SO_4, and the solvent was evaporated under reduced pressure. Afterwards, the residue obtained was purified by MPLC (methanol:dichloromaethane (DCM) = 10:90), providing 3-benzylthiazol-2(3*H*)-imine **4a** as yellow oil.

1.2 Procedure for the synthesis of 3-benzyloxazol-2(3*H*)-imine **4b**

To a solution of 2-aminooxazole (1.0 eq., 1.1893 mmol, 100 mg) dissolved in 5 mL of acetone was added benzyl bromide (1.1 eq., 1.3082 mmol, 223 mg). The reaction mixture was heated at 60 ^oC for 6 h. After cooling, the reaction was concentrated under reduced pressure by rotary evaporator. The residue obtained was dried under high vacuum pump providing 3-benzyloxazol-2(3*H*)-imine **4b** as a yellow oil. The crude product was used further without purification.

1.3 General procedure for the synthesis of compounds **5a-f, 5k-m** and **6c-e**

To a solution containing carboxylic acid (0.8 eq.) dissolved in 4 mL of DMF was added 1-[bis(dimethylamino)methylene]-1*H*-1,2,3-triazolo[4,5-*b*]pyridinium 3-oxid hexafluorophosphate (HATU) (1.5 eq.) at 0 ^oC. After the reaction was stirred for 30 min, *N,N*-diisopropylethylamine (DIPEA) (3.0 eq.) and imine (1.0 eq.) were added to the reaction mixture. The reaction temperature was increased to rt and stirred for overnight. After the reaction was completed, it was quenched by addition of water. In case solid formation was observed, it was filtered out and dried. Other than that, the reaction mixture was extracted with EA, the organic layer was collected and washed with a brine. Subsequently, the organic phase was dried using anhydrous Na_2SO_4, and the solvent was evaporated under reduced pressure. The product was further purified by MPLC (methanol:DCM = 10:90) or by preparatory TLC (methanol:DCM = 5:95), providing compounds **5a-f, 5k-m** and **6c-e**.

1.4 General procedure for the synthesis of compounds **6a-b**

To a solution containing carboxylic acid (1.0 eq.) dissolved in pyridine (40 eq.) was added *N,N,N',N'*-Tetramethyl-O-(1*H*-benzotriazol-1-yl)uronium hexafluorophosphate (HBTU) (2.0 eq.) at 0 ^oC. After the reaction was stirred for 30 min, 2-aminothiazole (1.5 eq.) was added to the reaction mixture. The reaction temperature was gradually increased to 80 ^oC and stirred for

16 h. The reaction temperature was increased to 100 ºC and the reaction was stirred for an additional 5 h. After cooling down, the reaction was quenched by ice-cold water. The solid formed was filtered out, washed with water and acetonitrile, then dried, providing **6a-b**.

1.5 Procedure for the synthesis of (Z)-1-benzyl-*N*-(3-benzylthiazol-2(3*H*)-ylidene)-1*H*-pyrrolo[2,3-b]pyridine-3-carboxamide **5i**

Step 1.

(Z)-*N*-(3-benzylthiazol-2(3*H*)-ylidene)-1*H*-pyrrolo[2,3-*b*]pyridine-3-carboxamide (Truli) **1** was synthesized according to the reported procedure.[1] All the analytical data were compared and matched with the reference compound Truli.

Step 2.

A compound **1** (1.0 eq., 0.0598 mmol, 20 mg) obtained in the abovementioned step was dissolved in 1 mL of DMF, and sodium hydride (60% in mineral oil) (1.5 eq., 3.6 mg) was added to the resulting solution at 0 ºC. After 10 min, benzyl bromide (1.2 eq., 0.0718 mmol, 12 mg) was added to the reaction mixture. The reaction was stirred at rt for 1.5 h. After cooling, the reaction was quenched by slowly addition of saturated aqueous ammonium chloride solution. The reaction was extracted with EA and water, the organic layer was collected and washed with brine. Afterwards, the organic layer was dried using anhydrous Na_2SO_4, and the solvent was evaporated under reduced pressure. Afterwards, the residue obtained was purified by MPLC (methanol:DCM = 10:90), providing (Z)-1-benzyl-*N*-(3-benzylthiazol-2(3*H*)-ylidene)-1*H*-pyrrolo[2,3-*b*]pyridine-3-carboxamide **5i** as a white solid.

1.6 General procedure for the synthesis of compounds **5g-h** and **5j**

A corresponding carboxamide (1.0 eq.) was dissolved in DMF, and benzyl bromide (1.2 eq.) was added to the resulting solution. The reaction mixture was heated to 60 ºC and stirred for overnight. After cooling down, the reaction mixture was diluted with EA and aqueous 10 M NaOH at 0 ºC. The reaction mixture was stirred for additional 1 h. If the formation of solid was observed, the solid was filtered out, washed with water and dried. If the formation of the solid was not observed, the reaction mixture was extracted with EA and water, the organic layer was collected, washed with brine and dried using anhydrous Na_2SO_4. The solvent was removed

using rotary evaporator and dried under high vacuum. The product was purified by MPLC (methanol:DCM = 10:90), providing corresponding compounds **5g-h** and **5j**.

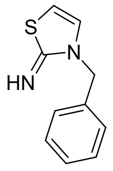

4a

3-benzylthiazol-2(3*H*)-imine (4a): Yield: 67%; ^{1}H NMR (300 MHz, $CDCl_3$) δ 7.40–7.27 (m, 5H), 6.35 (d, J = 5.0 Hz, 1H), 5.79 (d, J = 5.0 Hz, 1H), 4.91 (s, 2H); ^{13}C NMR (101 MHz, $CDCl_3$) δ 164.89, 136.71, 128.97, 127.93, 127.85, 127.08, 97.96, 49.11; LCMS (ESI) *m/z*: 191.00 $[M+H]^+$.

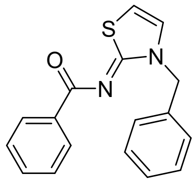

5a

(Z)-*N*-(3-benzylthiazol-2(3*H*)-ylidene)benzamide (5a): Yield: 56%; m.p.: 68-69 ºC; HPLC: 95.9 % (t_R =10.0 min); ^{1}H NMR (300 MHz, $CDCl_3$) δ 8.40–8.33 (m, 2H), 7.51–7.41 (m, 3H), 7.39–7.31 (m, 5H), 6.96 (d, J = 4.8 Hz, 1H), 6.65 (d, J = 4.7 Hz, 1H), 5.50 (s, 2H); ^{13}C NMR (101 MHz, $CDCl_3$) δ 174.29, 168.28, 137.04, 135.73, 133.60, 131.61, 130.26, 129.44, 129.20, 128.58, 128.31, 128.18, 125.72, 109.54, 52.12; LCMS (ESI) *m/z*: 295.1 $[M+H]^+$.

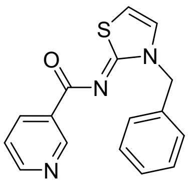

5b

(Z)-*N*-(3-benzylthiazol-2(3*H*)-ylidene)nicotinamide (5b): Yield: 56%; m.p.: 69-70 ºC; HPLC: 99.0 % (t_R =2.7 min); ^{1}H NMR (300 MHz, $CDCl_3$) δ 9.54 (dd, J = 2.1, 0.9 Hz, 1H), 8.70 (dd, J = 4.8, 1.8 Hz, 1H), 8.54 (dt, J = 7.9, 2.1 Hz, 1H), 7.41–7.31 (m, 6H), 7.02 (d, J = 4.7 Hz, 1H), 6.72 (d, J = 4.7 Hz, 1H), 5.51 (s, 2H); ^{13}C NMR (101 MHz, $CDCl_3$) δ 172.58, 168.27, 151.88, 151.08, 136.85, 135.38, 132.50, 129.25, 128.69, 128.23, 126.06, 123.28, 109.95, 52.30; LCMS (ESI) *m/z*: 296.10 $[M+H]^+$.

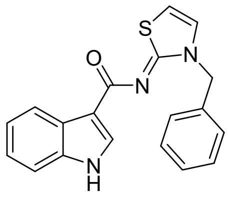

5c

(Z)-*N*-(3-benzylthiazol-2(3*H*)-ylidene)-1*H*-indole-3-carboxamide (5c): Yield: 27%; m.p.: 209-210 ºC; HPLC: 99.6% (t_R = 14.9 min); ^{1}H NMR (400 MHz, $CDCl_3$) δ 9.08 (s, 1H), 8.58–8.49 (m, 1H), 8.08 (d, J = 2.9 Hz, 1H), 7.42–7.30 (m, 6H), 7.24–7.16 (m, 2H), 6.87 (d, J = 4.8 Hz, 1H), 6.55 (d, J = 4.8 Hz, 1H), 5.48 (s, 2H); ^{13}C NMR (101 MHz, $CDCl_3$) δ 173.07, 166.91, 136.88, 135.88, 131.37, 129.11, 128.38, 128.16, 126.38, 125.44, 122.54, 122.22, 121.39, 116.06, 111.77, 108.85, 51.91; LCMS (ESI) *m/z*: 334.00 $[M+H]^+$.

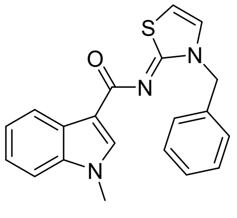

5d

(Z)-*N*-(3-benzylthiazol-2(3*H*)-ylidene)-1-methyl-1*H*-indole-3-carboxamide (5d): Yield: 9%; m.p.: 184-185 ºC; HPLC: 99.3% (t_R = 17.3 min); ^{1}H NMR (400 MHz, $CDCl_3$) δ 8.52 (d, J = 7.1 Hz, 1H), 7.96 (s, 1H), 7.40–7.31 (m, 5H), 7.30–7.20 (m, 3H), 6.88 (d, J = 4.8 Hz, 1H), 6.57 (d, J = 4.8 Hz, 1H), 5.51 (s, 2H), 3.84 (s, 3H); ^{13}C NMR (125 MHz, $CDCl_3$) δ 172.67, 166.88, 137.73, 136.08, 135.16, 129.19, 128.46, 128.22, 127.25, 125.25, 122.68, 122.36, 121.40, 114.90, 109.58, 108.82, 51.92, 33.47; LCMS (ESI) *m/z*: 348.1 $[M+H]^+$.

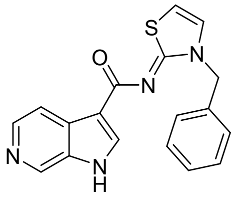

5e

(Z)-*N*-(3-benzylthiazol-2(3*H*)-ylidene)-1*H*-pyrrolo[2,3-*c*]pyridine-3-carboxamide (5e): Yield: 29%; m.p.: 149-150 ºC; HPLC: 98.2% (t_R = 4.5 min); ^{1}H NMR (500 MHz, MeOD-d_4) δ 8.68 (s, 1H), 8.25 (s, 1H), 8.23 (d, J = 5.6 Hz, 1H), 8.09 (d, J = 5.6 Hz, 1H), 7.39–7.26 (m,

5H), 7.24 (d, J = 4.6 Hz, 1H), 6.83 (d, J = 4.6 Hz, 1H), 5.50 (s, 2H); ^{13}C NMR (101 MHz, MeOD-d_4) δ 173.27, 168.70, 138.20, 137.69, 137.63, 135.28, 134.12, 133.80, 129.95, 129.17, 128.78, 128.22, 118.13, 116.82, 110.51, 52.87; LCMS (ESI) *m/z*: 335.00 $[M+H]^+$.

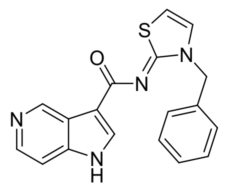

5f

(Z)-*N*-(3-benzylthiazol-2(3*H*)-ylidene)-1*H*-pyrrolo[3,2-*c*]pyridine-3-carboxamide (5f): Yield: 41%; HPLC: 98.8% (t_R = 4.7 min); ^{1}H NMR (400 MHz, DMSO-d_6) δ 9.52 (s, 1H), 8.28 (d, J = 5.8 Hz, 1H), 8.25 (s, 1H), 7.62 (d, J = 4.7 Hz, 1H), 7.53 (d, J = 5.8 Hz, 1H), 7.43 (d, J = 7.1 Hz, 2H), 7.36 (t, J = 7.4 Hz, 2H), 7.29 (t, J = 7.3 Hz, 1H), 7.01 (d, J = 4.7 Hz, 1H), 5.55 (s, 2H); ^{13}C NMR (101 MHz, DMSO-d_6) δ 170.43, 166.05, 143.04, 140.66, 139.45, 136.73, 132.95, 128.74, 127.84, 127.79, 127.31, 122.66, 115.31, 108.79, 107.78, 50.94; LCMS (ESI) *m/z*: 335.00 $[M+H]^+$.

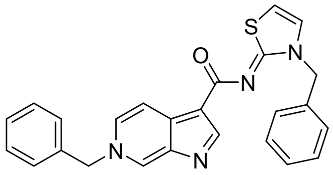

5g

(Z)-6-benzyl-*N*-(3-benzylthiazol-2(3*H*)-ylidene)-6*H*-pyrrolo[2,3-*c*]pyridine-3-carboxamide (5g): Yield: 9%; HPLC: 97.2 % (t_R = 10.4 min); ^{1}H NMR (400 MHz, $CDCl_3$) δ 9.33 (s, 1H), 8.73 (d, J = 6.7 Hz, 1H), 8.66 (s, 1H), 8.10 (s, 1H), 7.95 (d, J = 6.7 Hz, 1H), 7.50–7.27 (m, 10H), 7.02 (d, J = 4.7 Hz, 1H), 6.72 (d, J = 4.7 Hz, 1H), 5.61 (s, 2H), 5.47 (s, 2H); ^{13}C NMR (101 MHz, $CDCl_3$) δ 170.85, 169.85, 142.60, 135.82, 135.18, 132.58, 131.42, 130.44, 130.09, 129.64, 129.37, 128.80, 128.74, 127.98, 126.22, 119.85, 118.27, 109.76, 65.00, 52.36; LCMS (ESI) *m/z*: 425.20 $[M+H]^+$.

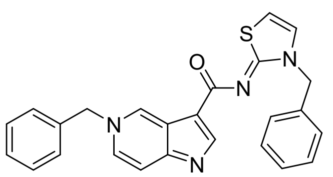

5h

(Z)-5-benzyl-*N*-(3-benzylthiazol-2(3*H*)-ylidene)-5*H*-pyrrolo[3,2-*c*]pyridine-3-carboxamide (5h): Yield: 64%; m.p.: 189-190 ºC; HPLC: 99.0% (t_R = 10.1 min); ^{1}H NMR (300 MHz, $CDCl_3$) δ 9.23 (s, 1H), 8.86 (s, 1H), 7.69 (d, J = 6.8 Hz, 1H), 7.52 (dd, J = 6.8, 1.9 Hz, 1H), 7.43–7.27 (m, 8H), 7.16 (dd, J = 6.8, 2.9 Hz, 2H), 6.90 (d, J = 4.8 Hz, 1H), 6.54 (d, J = 4.8 Hz, 1H), 5.44 (s, 2H), 5.37 (s, 2H); ^{13}C NMR (101 MHz, $CDCl_3$+MeOD-d^4) δ 172.20, 166.70, 135.75, 135.61, 134.54, 129.33, 129.21, 129.1, 129.03, 128.33, 127.86, 127.59, 125.70, 113.94, 108.75, 62.96, 51.79; LCMS (ESI) *m/z*: 425.10 [M+H]$^+$.

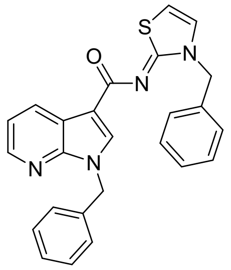

5i

(Z)-1-benzyl-*N*-(3-benzylthiazol-2(3*H*)-ylidene)-1*H*-pyrrolo[2,3-*b*]pyridine-3-carboxamide (5i): Yield: 61%; m.p.: 95-96 ºC; HPLC: 98.1% (t_R = 17.7 min); ^{1}H NMR (400 MHz, $CDCl_3$) δ 8.68 (dd, J = 7.9, 1.6 Hz, 1H), 8.34 (dd, J = 4.7, 1.6 Hz, 1H), 8.03 (s, 1H), 7.35–7.20 (m, 10H), 7.15 (dd, J = 7.9, 4.7 Hz, 2H), 6.87 (d, J = 4.8 Hz, 1H), 6.56 (d, J = 4.8 Hz, 1H), 5.50 (s, 2H), 5.44 (s, 2H); ^{13}C NMR (101 MHz, $CDCl_3$) δ 172.09, 167.13, 148.43, 143.70, 137.08, 135.80, 133.60, 130.84, 129.19, 128.93, 128.51, 128.12, 127.97, 127.85, 125.46, 119.67, 117.75, 114.00, 109.08, 52.02, 48.34; LCMS (ESI) *m/z*: 425.3 [M+H]$^+$.

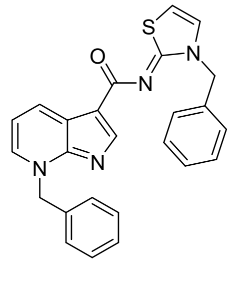

5j

(Z)-7-benzyl-*N*-(3-benzylthiazol-2(3*H*)-ylidene)-7*H*-pyrrolo[2,3-*b*]pyridine-3-carboxamide (5j): Yield: 63%**;** m.p.: 107-108 ºC; HPLC: 96.5% (t_R = 10.4 min); ^{1}H NMR (400 MHz, $CDCl_3$) δ 8.87 (dd, J = 7.5, 1.2 Hz, 1H), 8.75 (s, 1H), 7.59 (dd, J = 6.3, 1.2 Hz, 1H), 7.38–7.27 (m, 10H), 6.95 (dd, J = 7.5, 6.3 Hz, 1H), 6.87 (d, J = 4.8 Hz, 1H), 6.50 (d, J = 4.8

Hz, 1H), 5.88 (s, 2H), 5.44 (s, 2H); ^{13}C NMR (101 MHz, $CDCl_3$) δ 172.42, 166.39, 151.60, 150.81, 136.03, 134.82, 133.59, 129.56, 129.30, 129.15, 129.03, 128.80, 128.51, 128.28, 128.16, 125.33, 115.80, 112.22, 108.37, 55.72, 51.86; LCMS (ESI) *m/z*: 425.00 $[M+H]^+$.

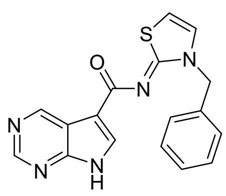

5k

(Z)-*N*-(3-benzylthiazol-2(3*H*)-ylidene)-7*H*-pyrrolo[2,3-*d*]pyrimidine-5-carboxamide (5k): Yield: 76%; m.p.: 224-225 ºC; HPLC: 99.2% (t_R = 3.1 min); ^{1}H NMR (300 MHz, $CDCl_3$) δ 10.22 (s, 1H), 9.73 (s, 1H), 8.94 (s, 1H), 8.18 (s, 1H), 7.32–7.40 (m, 5H), 6.97 (d, J = 4.7 Hz, 1H), 6.68 (d, J = 4.7 Hz, 1H), 5.53 (s, 2H); ^{13}C NMR (101 MHz, $CDCl_3$+MeOD-d_4) δ 171.19, 167.55, 151.85, 151.00, 150.91, 135.22, 132.07, 129.03, 128.37, 127.65, 126.09, 117.29, 114.90, 109.66, 51.99; HRMS (EI) calcd. for $C_{17}H_{13}N_5OS$ *m/z*: 335.0841, found *m/z*: 335.0837 $[M]^+$.

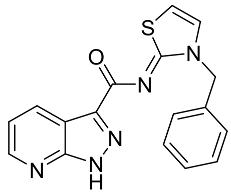

5l

(Z)-*N*-(3-benzylthiazol-2(3*H*)-ylidene)-1*H*-pyrazolo[3,4-*b*]pyridine-3-carboxamide (5l): Yield: 28%; m.p.: 244-245 ºC; HPLC: 98.8% (t_R = 9.5 min); ^{1}H NMR (300 MHz, $CDCl_3$) δ 12.13 (s, 1H), 8.79 (dd, J = 8.1, 1.6 Hz, 1H), 8.62 (dd, J = 4.6, 1.6 Hz, 1H), 7.33–7.37 (m, 5H), 7.25 (tr, J = 6 Hz, 1H), 6.98 (d, J = 4.7 Hz, 1H), 6.74 (d, J = 4.7 Hz, 1H), 5.60 (s, 2H); ^{13}C NMR (101 MHz, $CDCl_3$+MeOD-d_4) δ 169.58, 168.24, 152.28, 148.84, 135.24, 132.92, 129.12, 128.54, 128.07, 126.22, 118.32, 115.09, 110.28, 52.24; HRMS (EI) calcd. for $C_{17}H_{13}N_5OS$ *m/z*: 335.0841, found *m/z*: 335.0837 $[M]^+$.

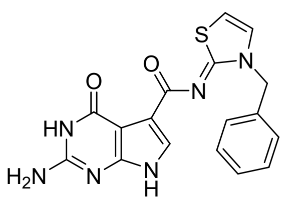

5m

(Z)-2-amino-*N*-(3-benzylthiazol-2(3*H*)-ylidene)-4-oxo-4,7-dihydro-3*H*-pyrrolo[2,3-*d*]pyrimidine-5-carboxamide (5m): Yield: 7%; HPLC: 95.0% (t_R = 1.5 min); ^{1}H NMR (300 MHz, MeOD-d_4) δ 7.66 (s, 1H), 7.34 (s, 6H), 6.61 (s, 1H), 5.42 (s, 2H); ^{13}C NMR (101 MHz, MeOD-d_4) δ 191.11, 165.76, 147.19, 135.96, 129.16, 128.63, 128.53, 127.75, 126.85, 126.19, 119.49, 106.63, 39.38; LCMS (ESI) *m/z*: 367.37 [M+H]$^+$.

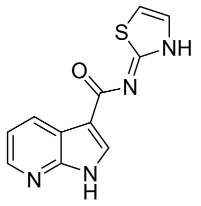

6a

(Z)-*N*-(thiazol-2(3*H*)-ylidene)-1*H*-pyrrolo[2,3-*b*]pyridine-3-carboxamide (6a): Yield: 56%; m.p.: 232-233 ^{o}C; HPLC: 97.1% (t_R = 2.4 min); ^{1}H NMR (300 MHz, DMSO-d_6) δ 12.45 (s, 1H), 12.27 (s, 1H), 8.68 (d, *J* = 2.7 Hz, 1H), 8.54 (dd, *J* = 7.9, 1.7 Hz, 1H), 8.34 (dd, *J* = 4.7, 1.7 Hz, 1H), 7.52 (d, *J* = 3.6 Hz, 1H), 7.25 (dd, *J* = 7.9, 4.7 Hz, 1H), 7.21 (d, *J* = 3.6 Hz, 1H); ^{13}C NMR (101 MHz, DMSO-d_6) δ 161.80, 158.67, 148.62, 144.12, 137.48, 130.59, 129.36, 118.76, 117.64, 113.03, 107.08; LCMS (ESI) *m/z*: 245.00 [M+H]$^+$.

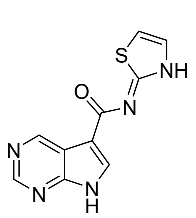

6b

(Z)-*N*-(thiazol-2(3*H*)-ylidene)-7*H*-pyrrolo[2,3-*d*]pyrimidine-5-carboxamide (6b): Yield: 69%; m.p.: 302-303 ^{o}C; HPLC: 99.3% (t_R = 0.98 min); ^{1}H NMR (400 MHz, DMSO-d_6) δ 12.86 (s, 1H), 12.46 (s, 1H), 9.47 (s, 1H), 8.89 (s, 1H), 8.71 (d, *J* = 2.6 Hz, 1H), 7.54 (d, *J* = 3.5 Hz,

1H), 7.25 (d, J = 3.5 Hz, 1H); ^{13}C NMR (101 MHz, DMSO-d_6) δ 161.03, 158.45, 152.25, 151.88, 150.18, 137.57, 131.41, 117.04, 113.36, 107.63; LCMS (ESI) *m/z*: 246.00 [M+H]$^+$.

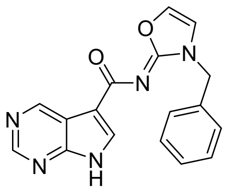

6c

(Z)-*N*-(3-benzyloxazol-2(3*H*)-ylidene)-7*H*-pyrrolo[2,3-*d*]pyrimidine-5-carboxamide (6c): Yield: 38%; m.p.: 210-211 ºC; HPLC: 95.4% (t_R = 1.3 min); ^{1}H NMR (400 MHz, DMSO-d_6) δ 12.52 (s, 1H), 9.39 (s, 1H), 8.79 (s, 1H), 8.13 (d, J = 2.5 Hz, 1H), 7.72 (d, J = 1.7 Hz, 1H), 7.54 (d, J = 1.7 Hz, 1H), 7.45–7.29 (m, 5H), 5.13 (s, 2H); ^{13}C NMR (101 MHz, DMSO-d_6) δ 168.39, 156.38, 151.95, 151.42, 150.21, 136.00, 132.19, 132.07, 128.80, 128.00, 127.74, 117.69, 116.76, 114.81, 47.98; LCMS (ESI) *m/z*: 320.10 [M+H]$^+$.

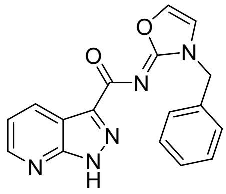

6d

(Z)-*N*-(3-benzyloxazol-2(3*H*)-ylidene)-1*H*-pyrazolo[3,4-*b*]pyridine-3-carboxamide (6d): Yield: 16%; m.p.: 170-171 ºC; HPLC: 99.7% (t_R = 4.5 min); ^{1}H NMR (400 MHz, DMSO-d_6) δ 8.52 (d, J = 4.4 Hz, 1H), 8.48 (dd, J = 8.1, 1.6 Hz, 1H), 7.80 (d, J = 1.7 Hz, 1H), 7.61 (d, J = 1.7 Hz, 1H), 7.45–7.30 (m, 5H), 7.21 (dd, J = 8.1, 4.5 Hz, 1H), 5.16 (s, 2H); ^{13}C NMR (101 MHz, DMSO-d_6) δ 166.67, 157.09, 152.34, 148.67, 141.86, 135.80, 132.55, 131.83, 128.83, 128.03, 127.61, 118.07, 118.00, 114.04, 48.17; LCMS (ESI) *m/z*: 320.00 [M+H]$^+$.

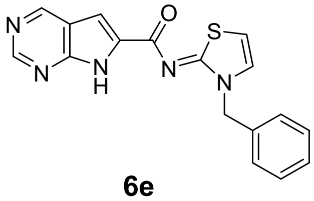

6e

(Z)-*N*-(3-benzylthiazol-2(3*H*)-ylidene)-7*H*-pyrrolo[2,3-*d*]pyrimidine-6-carboxamide (6e): Yield: 46%; m.p.: 249-250 ºC; HPLC: 95.0% (t_R = 4.3 min); ^{1}H NMR (300 MHz, DMSO-d_6)

δ 12.67 (s, 1H), 9.12 (s, 1H), 8.84 (s, 1H), 7.70 (d, J = 4.6 Hz, 1H), 7.47 (d, J = 7.0 Hz, 2H), 7.42–7.24 (m, 5H), 7.14 (d, J = 4.6 Hz, 1H), 5.66 (s, 2H); ^{13}C NMR (101 MHz, DMSO-d_6) δ 166.81, 166.51, 152.71, 151.38, 151.26, 137.08, 136.68, 128.71, 128.29, 128.23, 127.94, 127.83, 118.50, 110.04, 103.15, 50.93; LCMS (ESI) *m/z*: 336.37 $[M+H]^+$.

2. ^{1}H NMR and ^{13}C NMR spectra of **4a**, **5a-m** and **6a-e**.

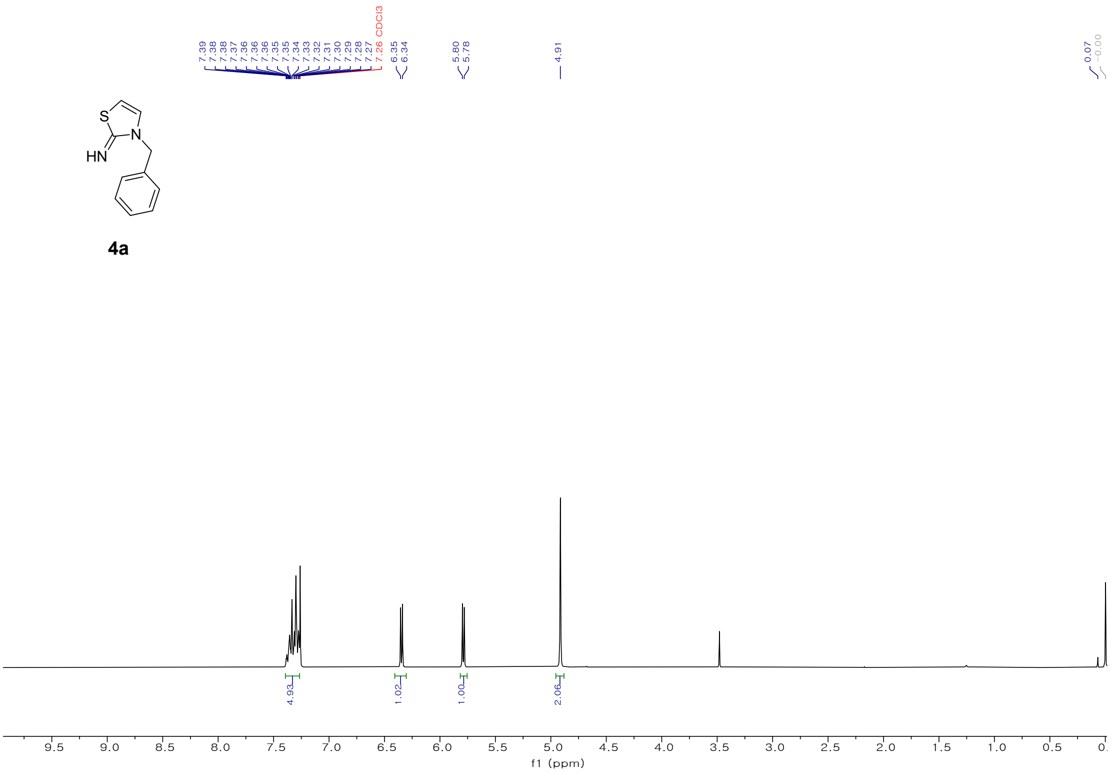

Figure S1. ^{1}H NMR of **4a** (300 MHz, $CDCl_3$).

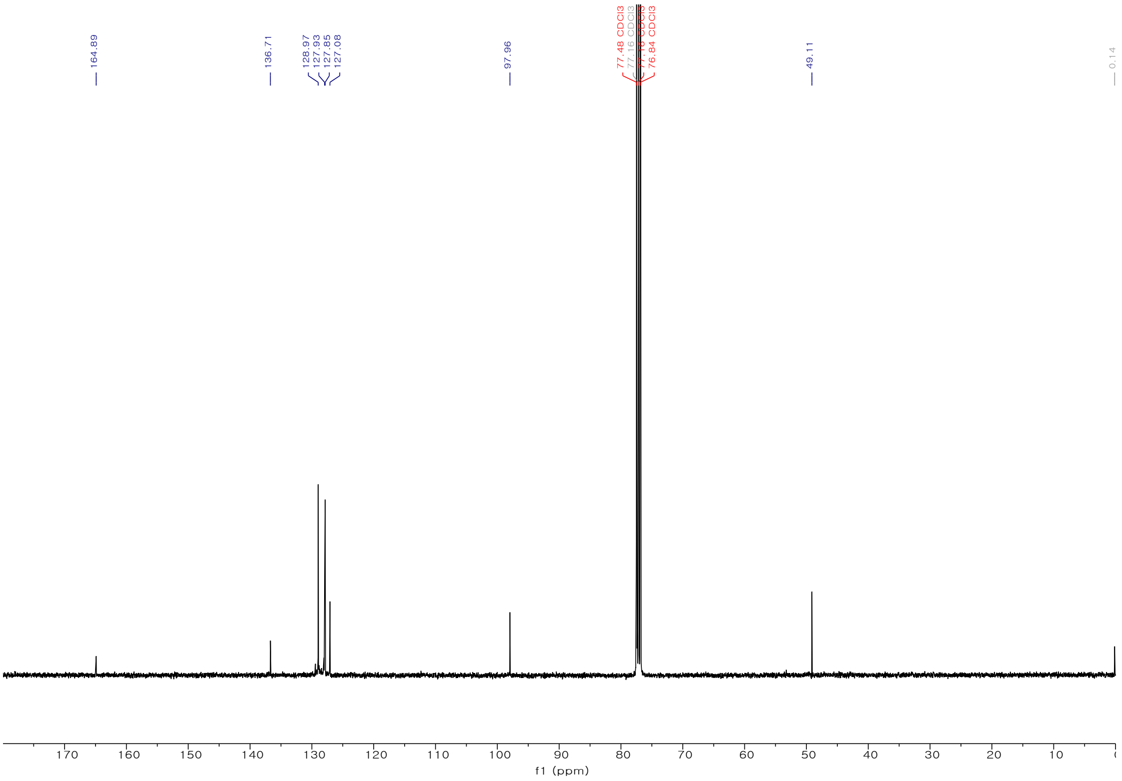

Figure S2. ^{13}C NMR of **4a** (101 MHz, $CDCl_3$).

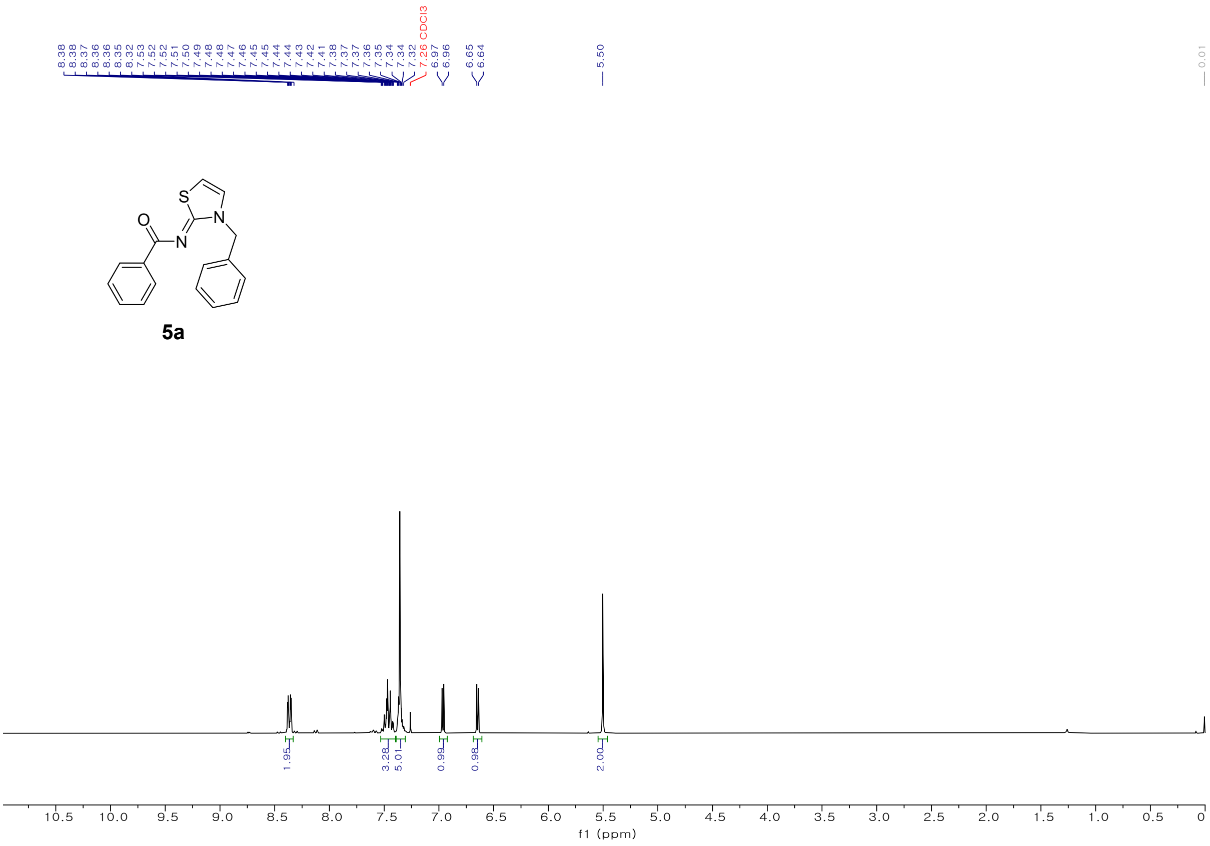

Figure S3. ^{1}H NMR of **5a** (300 MHz, $CDCl_3$).

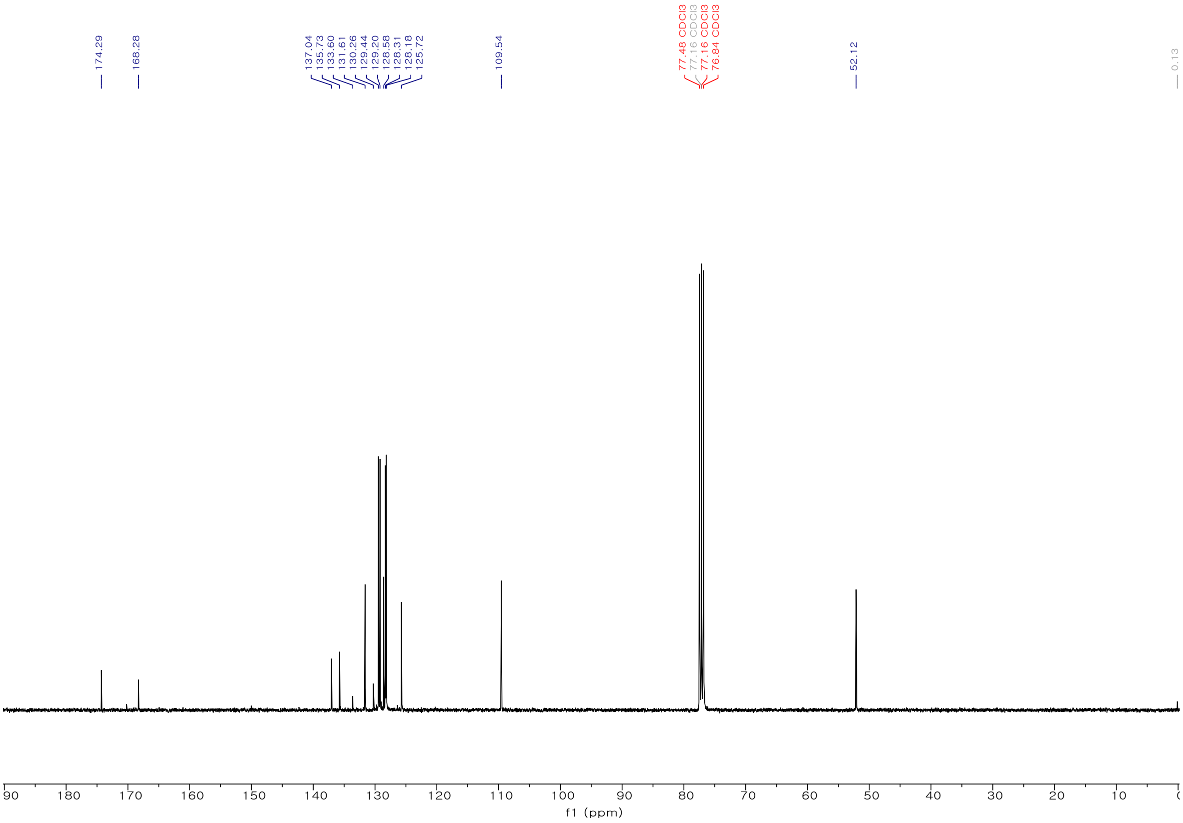

Figure S4. ^{13}C NMR of **5a** (101 MHz, $CDCl_3$).

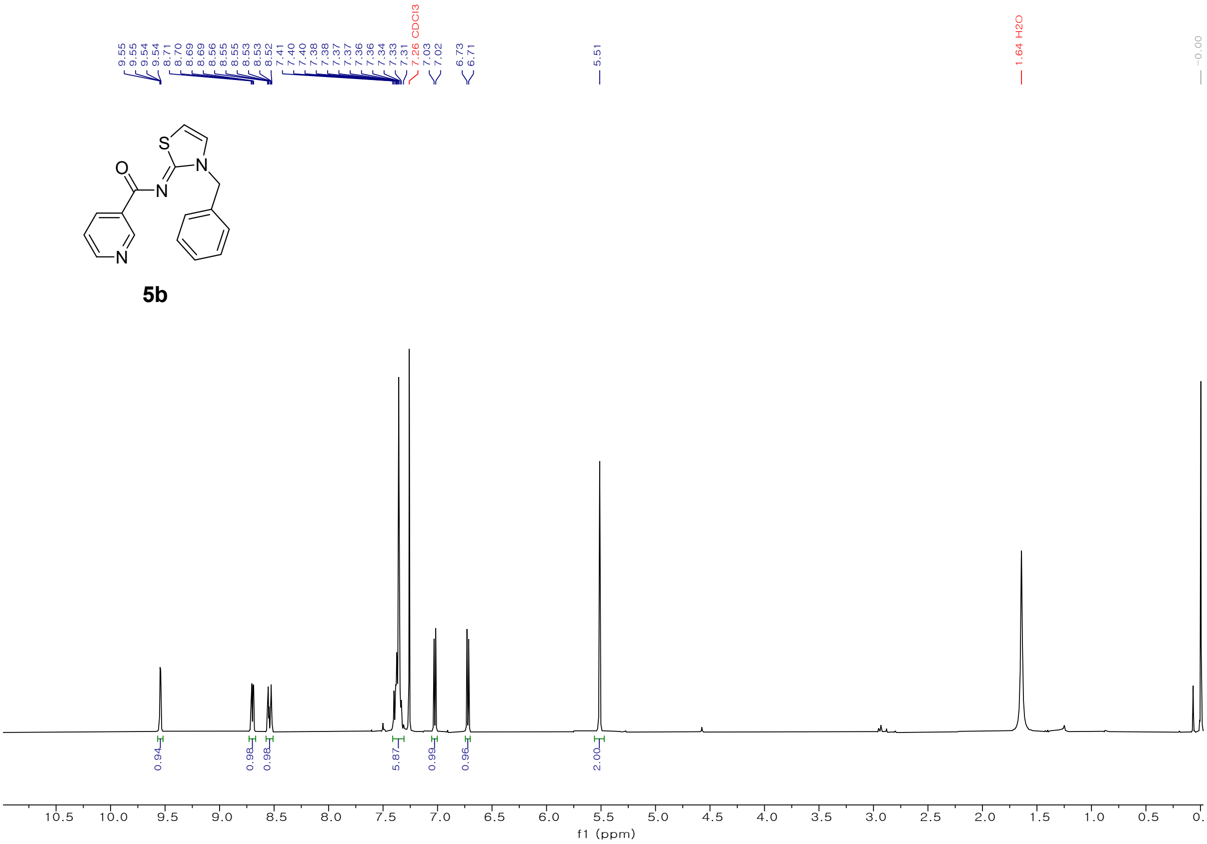

Figure S5. ^{1}H NMR of **5b** (300 MHz, $CDCl_3$).

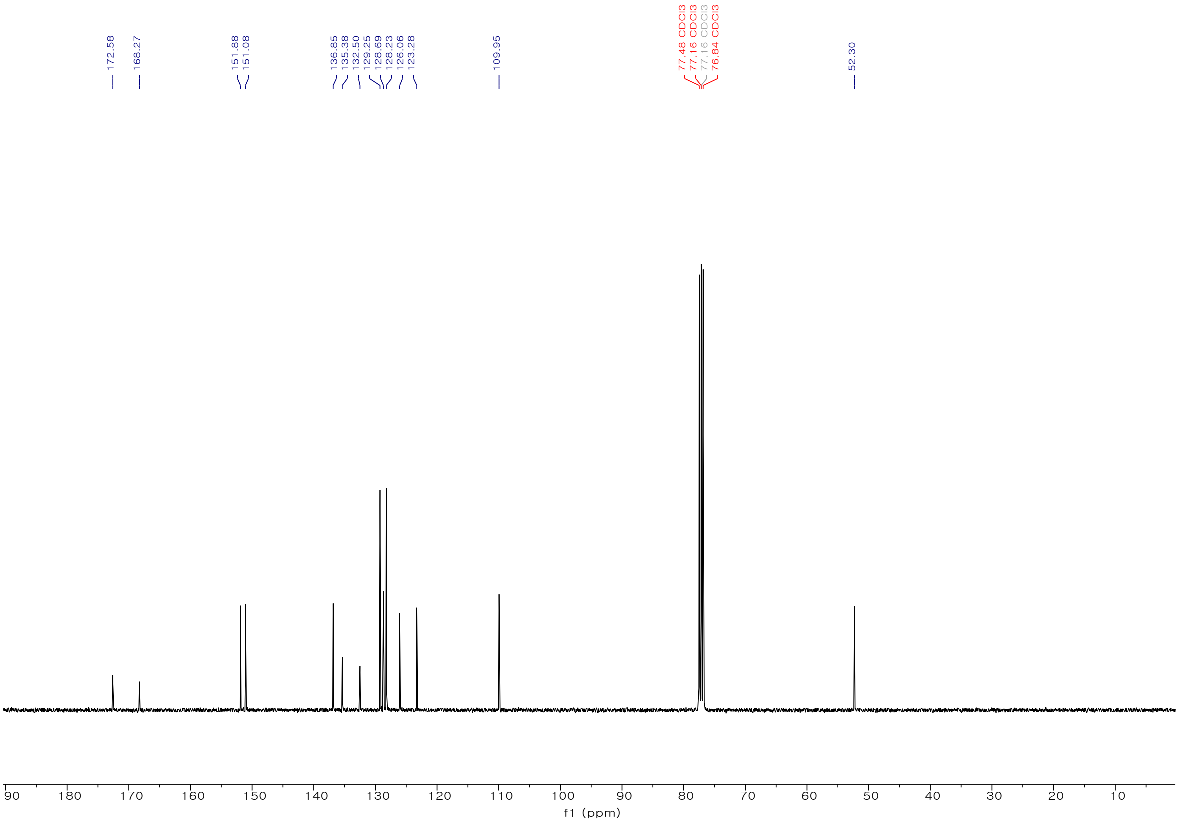

Figure S6. ^{13}C NMR of **5b** (101 MHz, $CDCl_3$).

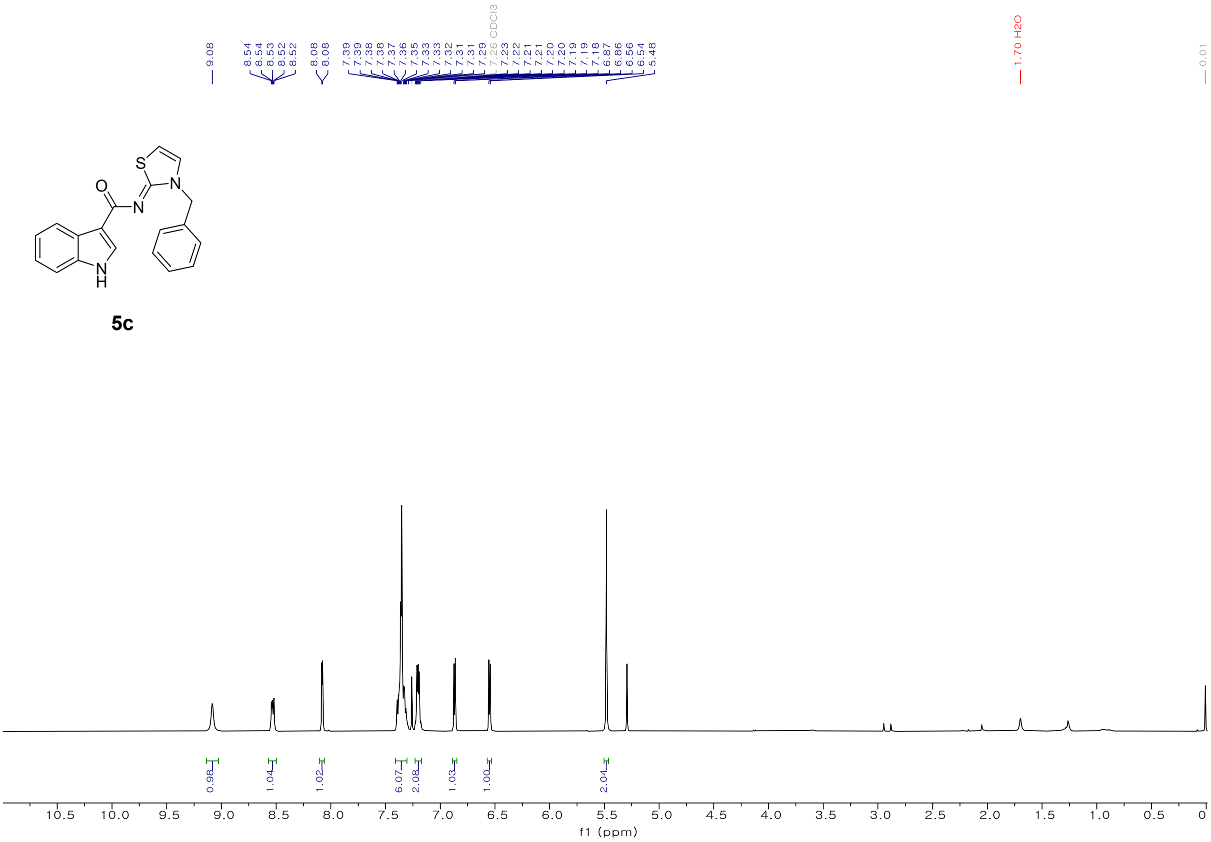

Figure S7. ¹H NMR of **5c** (400 MHz, $CDCl_3$).

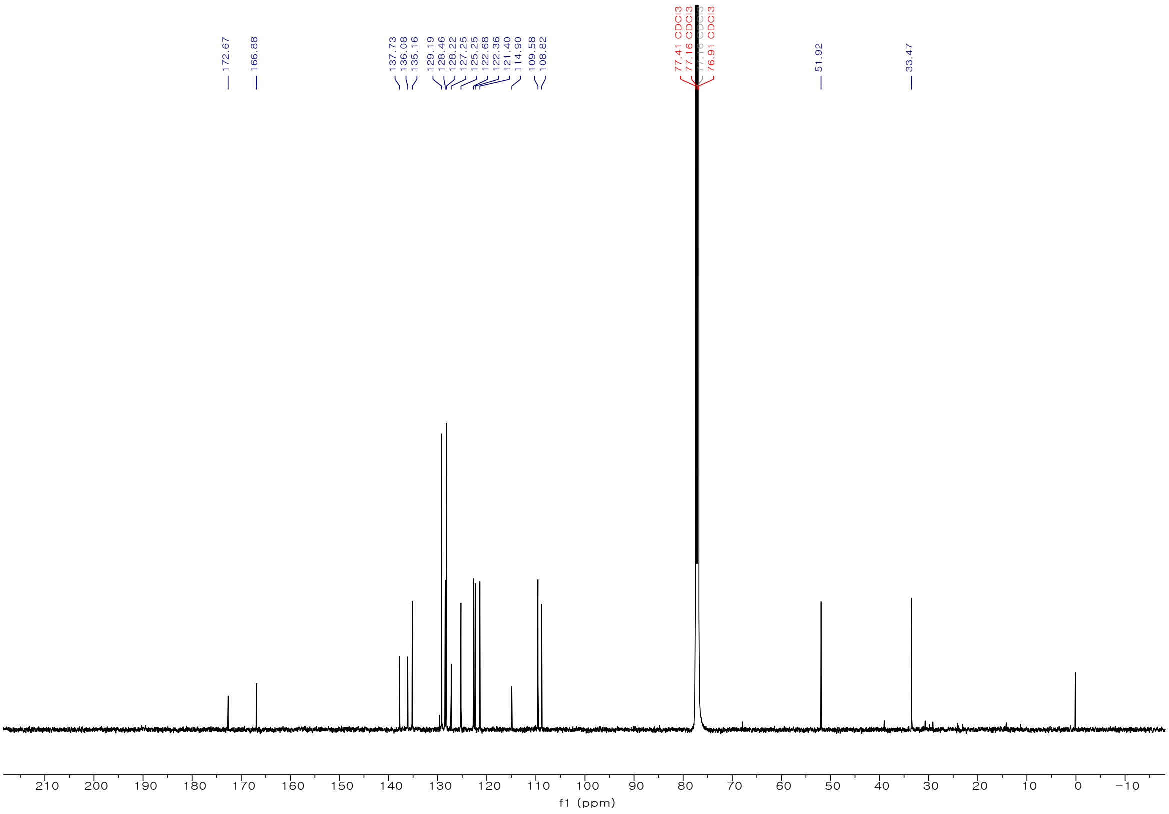

Figure S8. ^{13}C NMR of **5c** (125 MHz, $CDCl_3$).

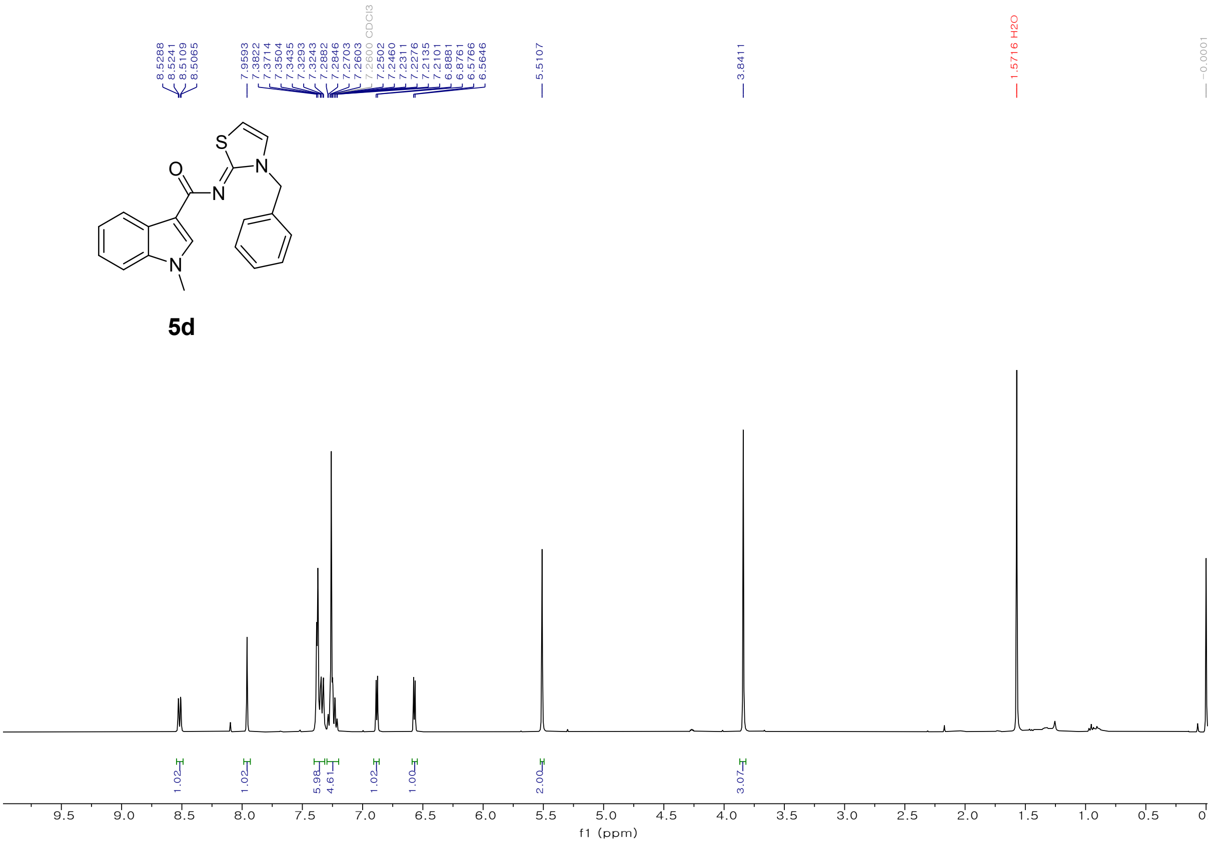

Figure S9. ^{1}H NMR of **5d** (400 MHz, $CDCl_3$).

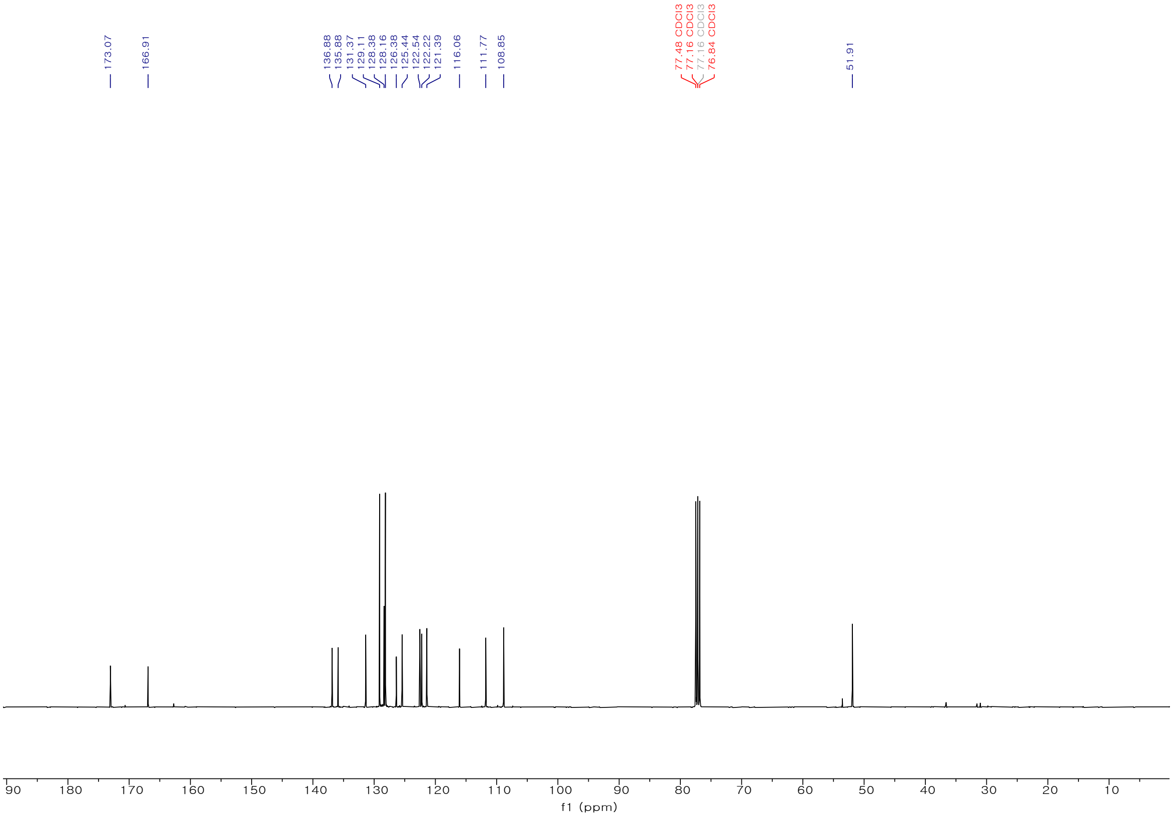

Figure S10. ^{13}C NMR of **5d** (101 MHz, $CDCl_3$).

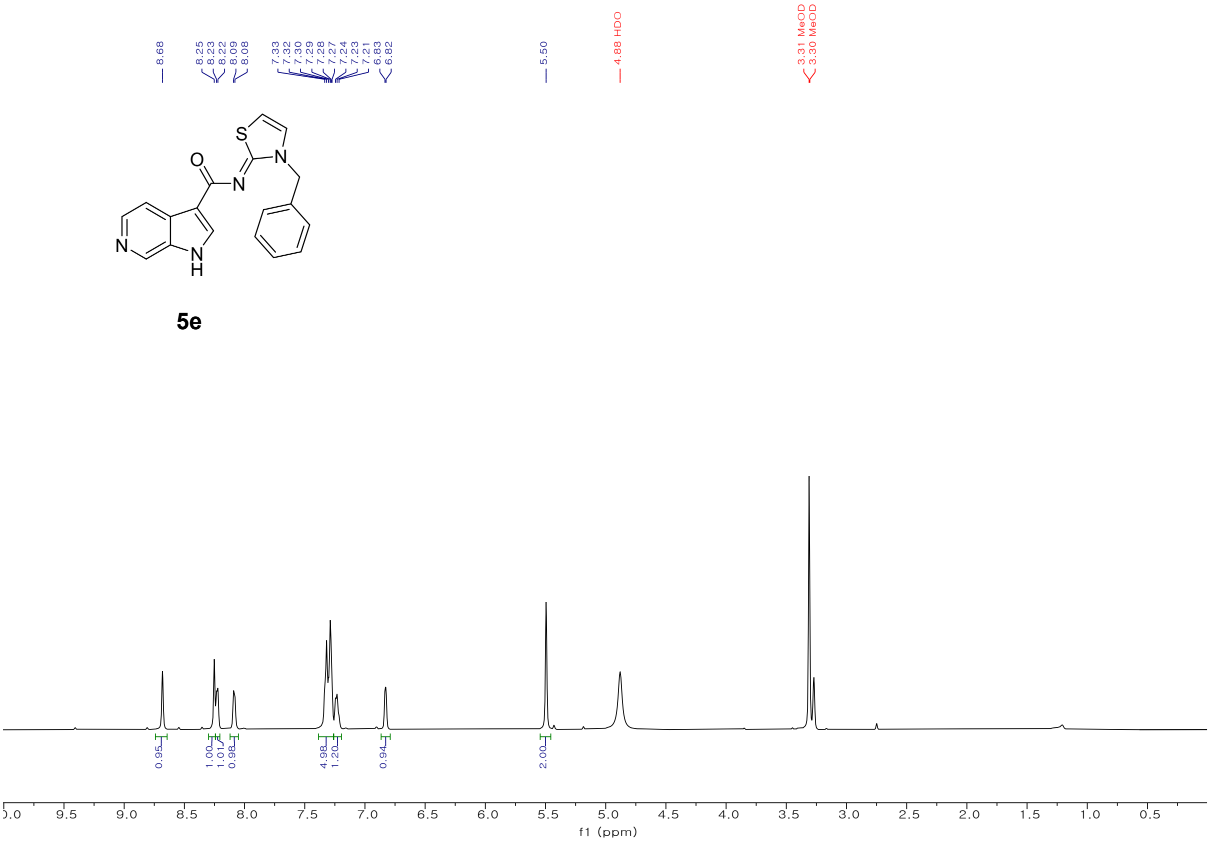

Figure S11. ^{1}H NMR of **5e** (500 MHz, MeOD-d_4).

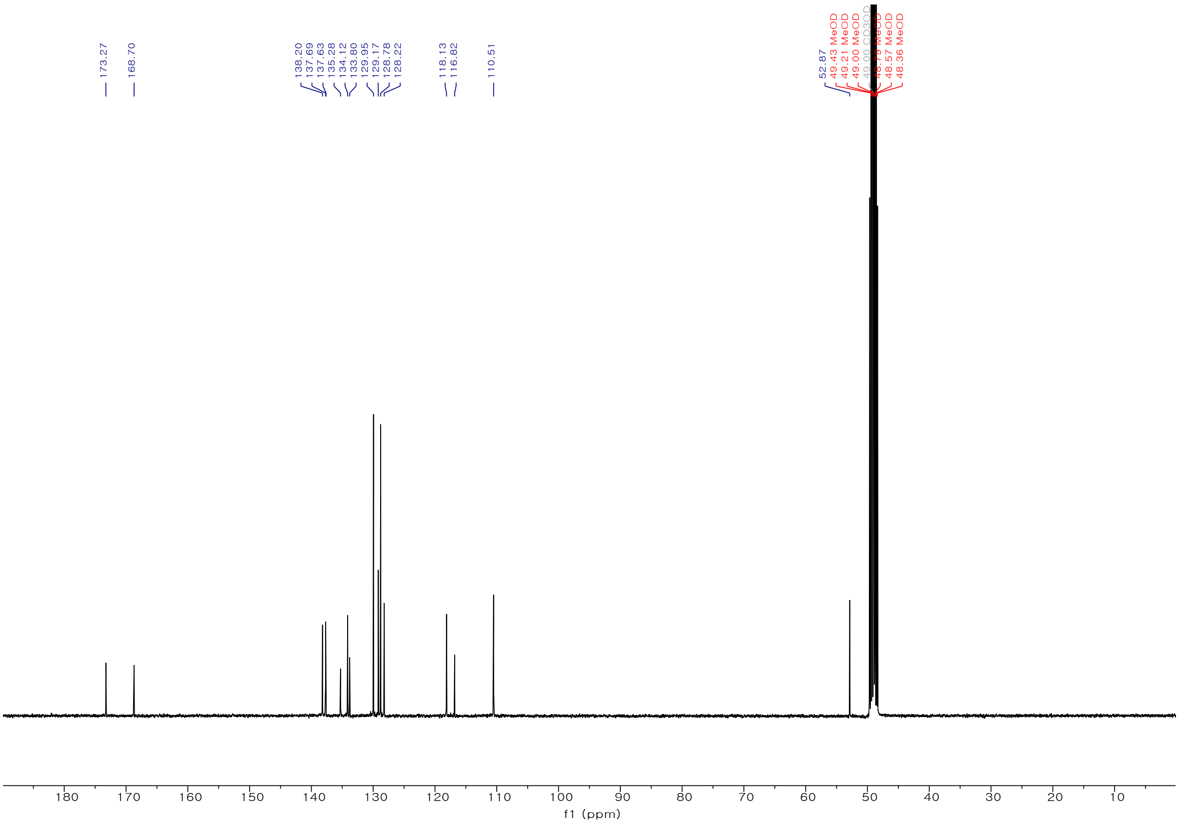

Figure S12. ^{13}C NMR of **5e** (101 MHz, MeOD-d_4).

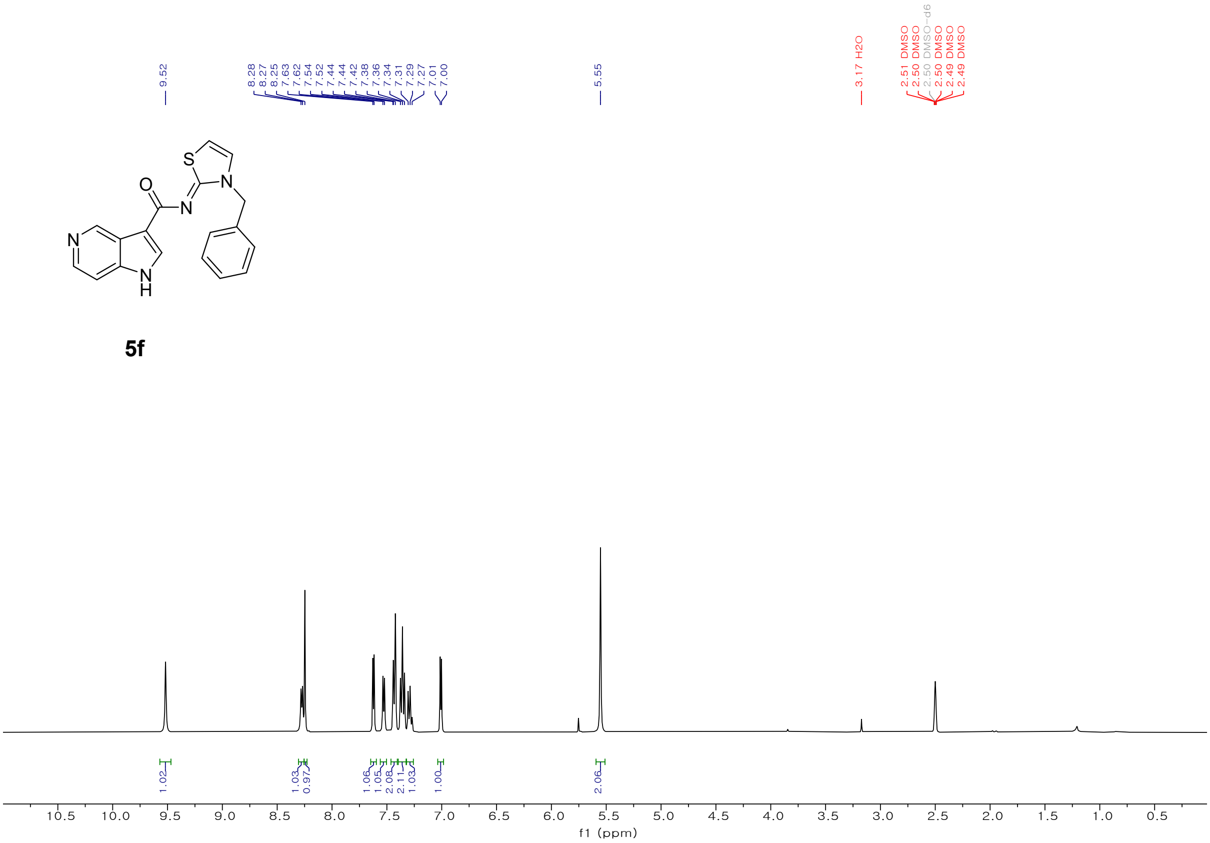

Figure S13. ^{1}H NMR of **5f** (400 MHz, DMSO-d_6).

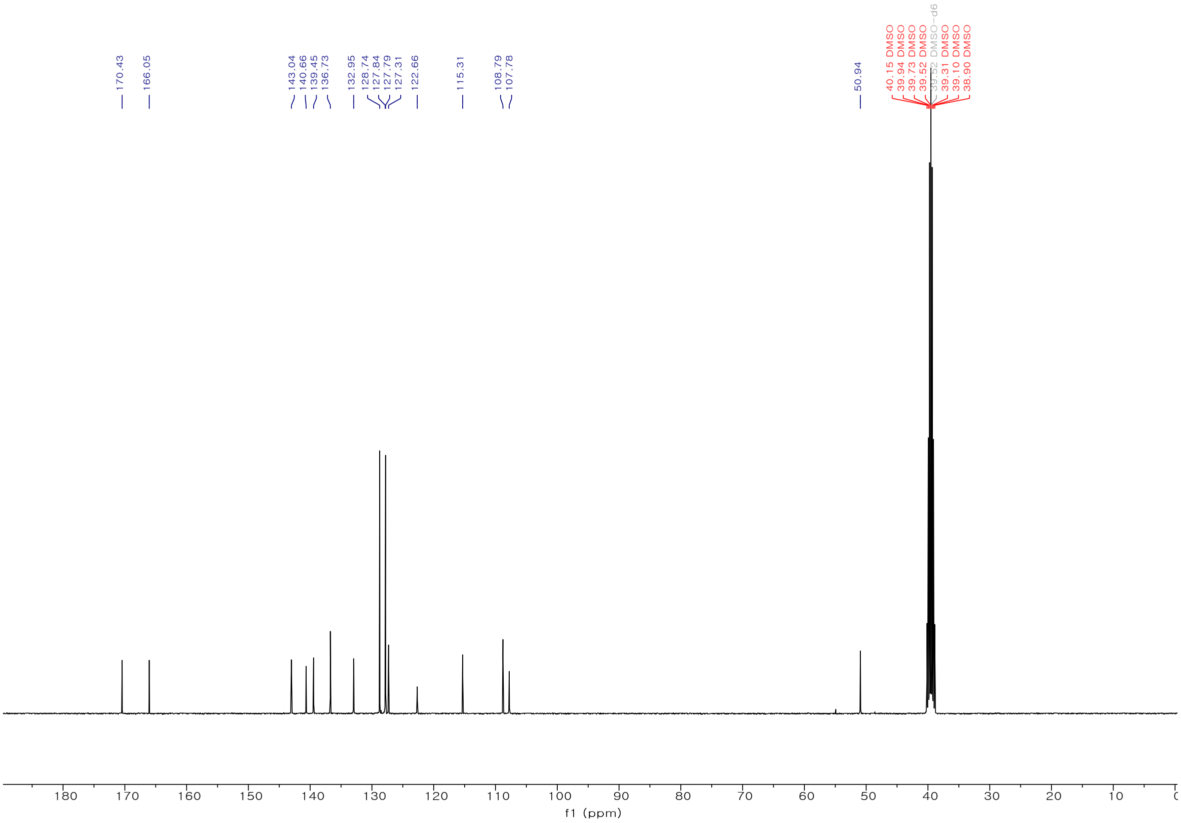

Figure S14. ^{13}C NMR of **5f** (101 MHz, DMSO-d_6).

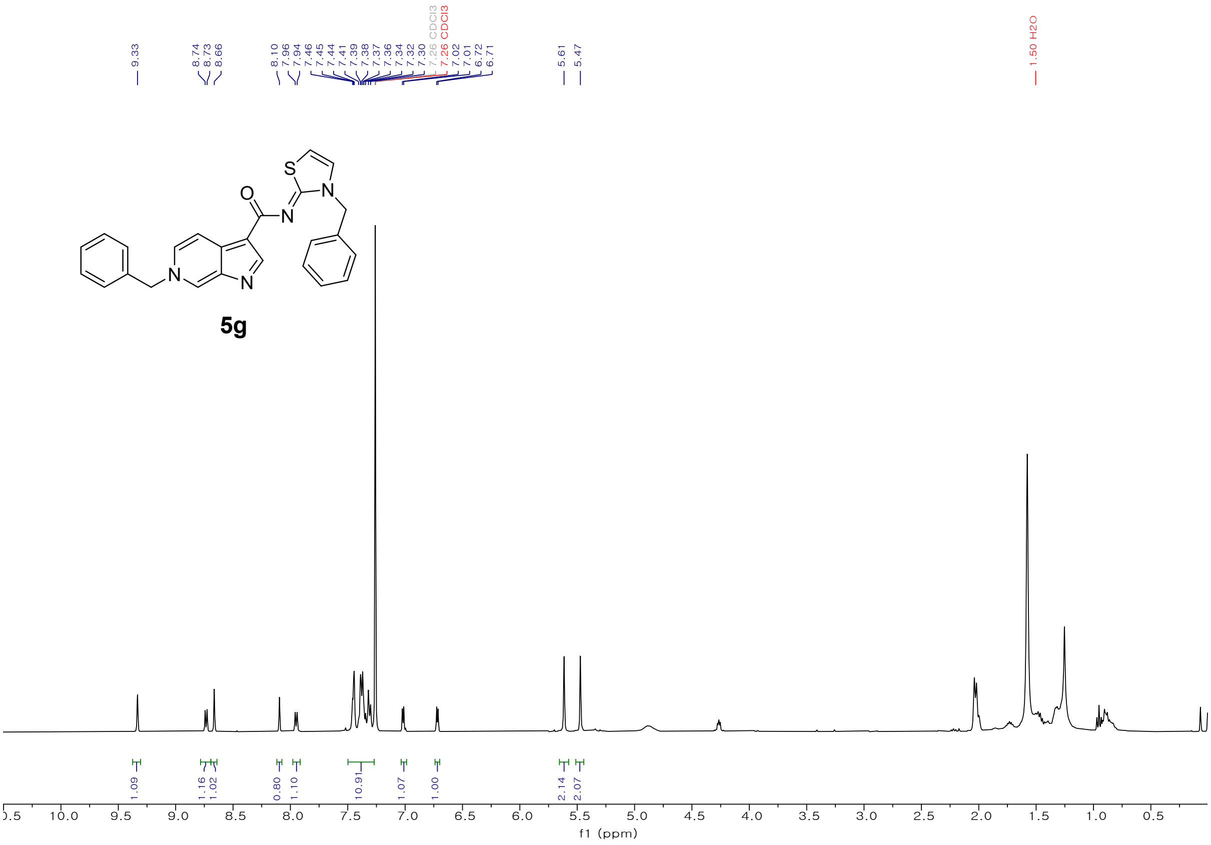

Figure S15. ^{1}H NMR of **5g** (400 MHz, $CDCl_3$).

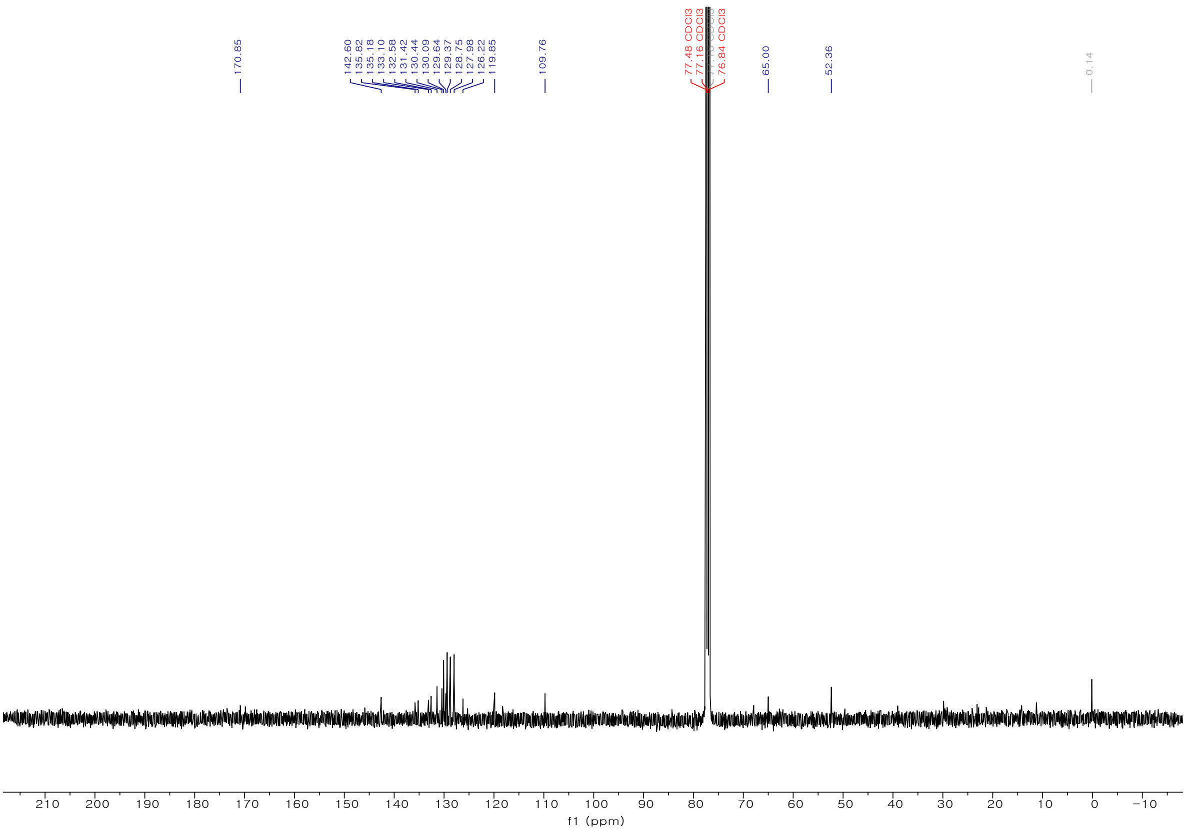

Figure S16. ^{13}C NMR of **5g** (101 MHz, $CDCl_3$).

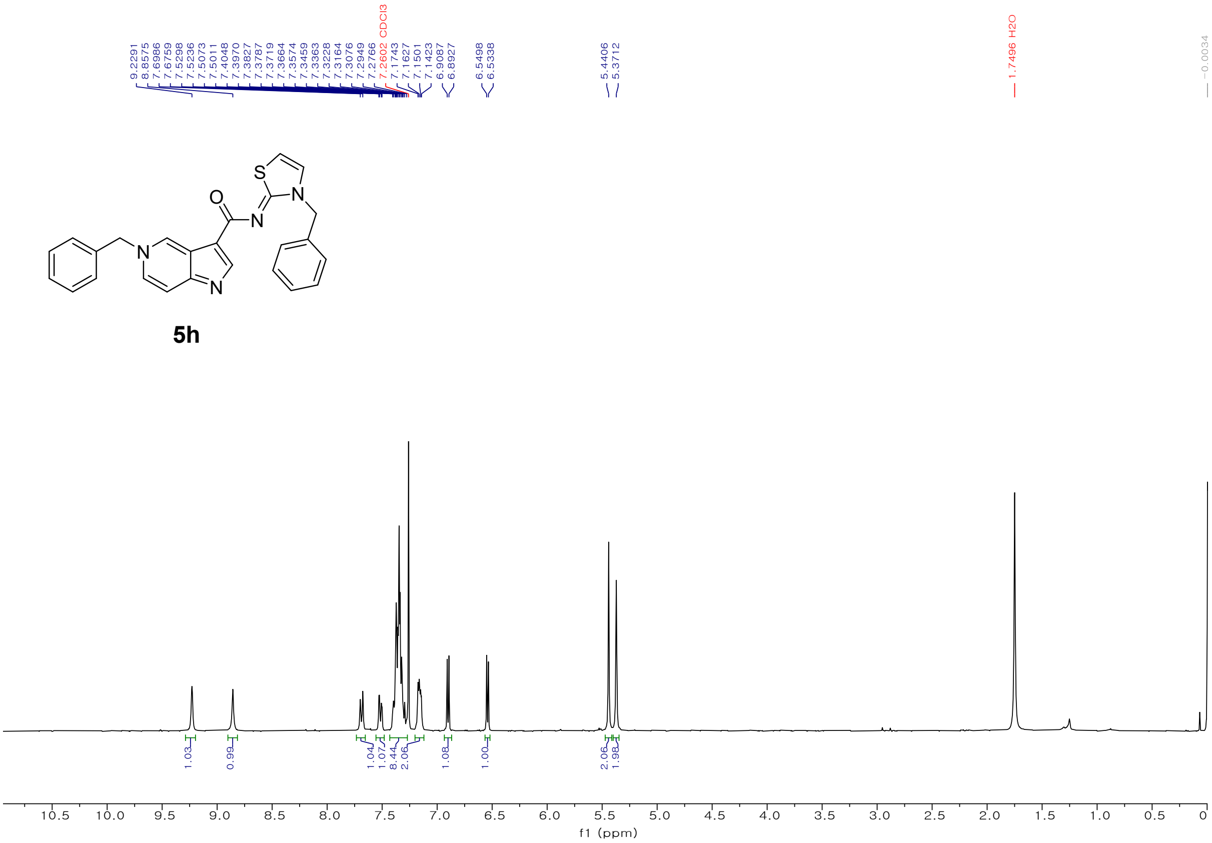

Figure S17. ^{1}H NMR of **5h** (400 MHz, $CDCl_3$).

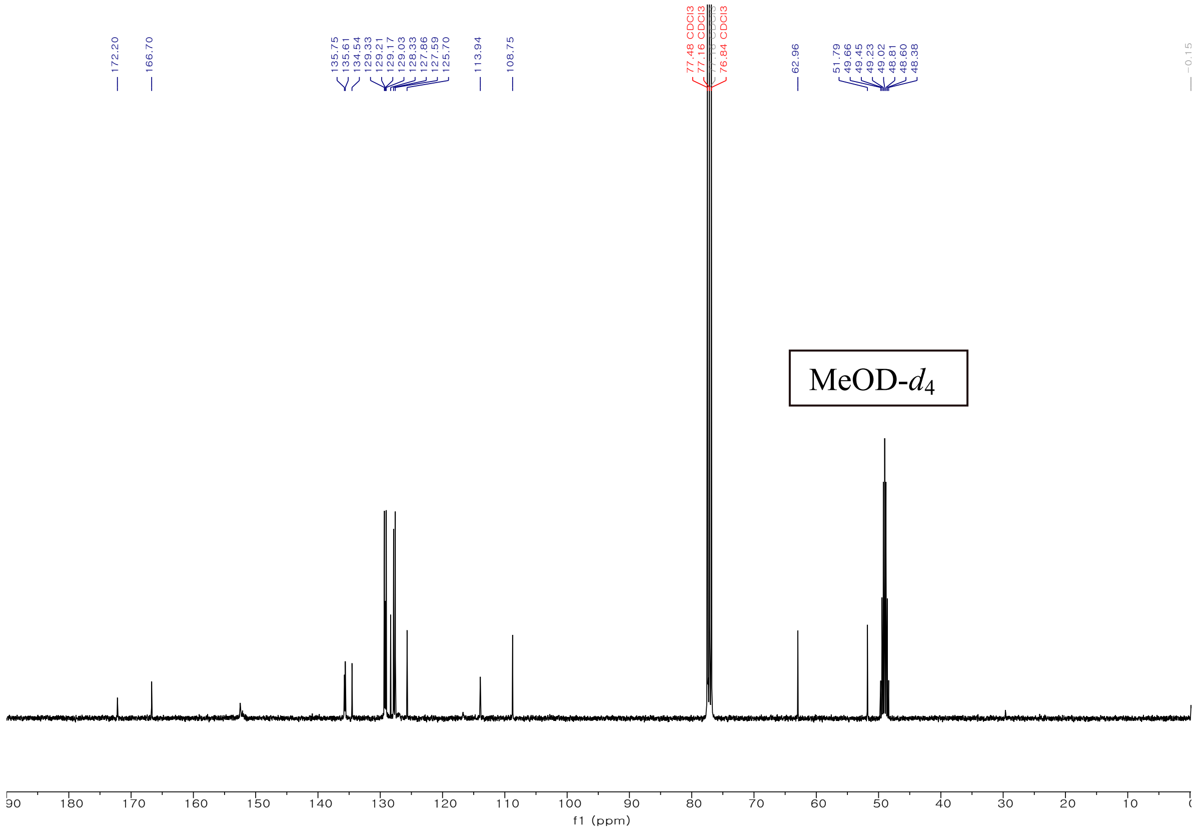

Figure S18. ^{13}C NMR of **5h** (101 MHz, $CDCl_3$+MeOD-d_4).

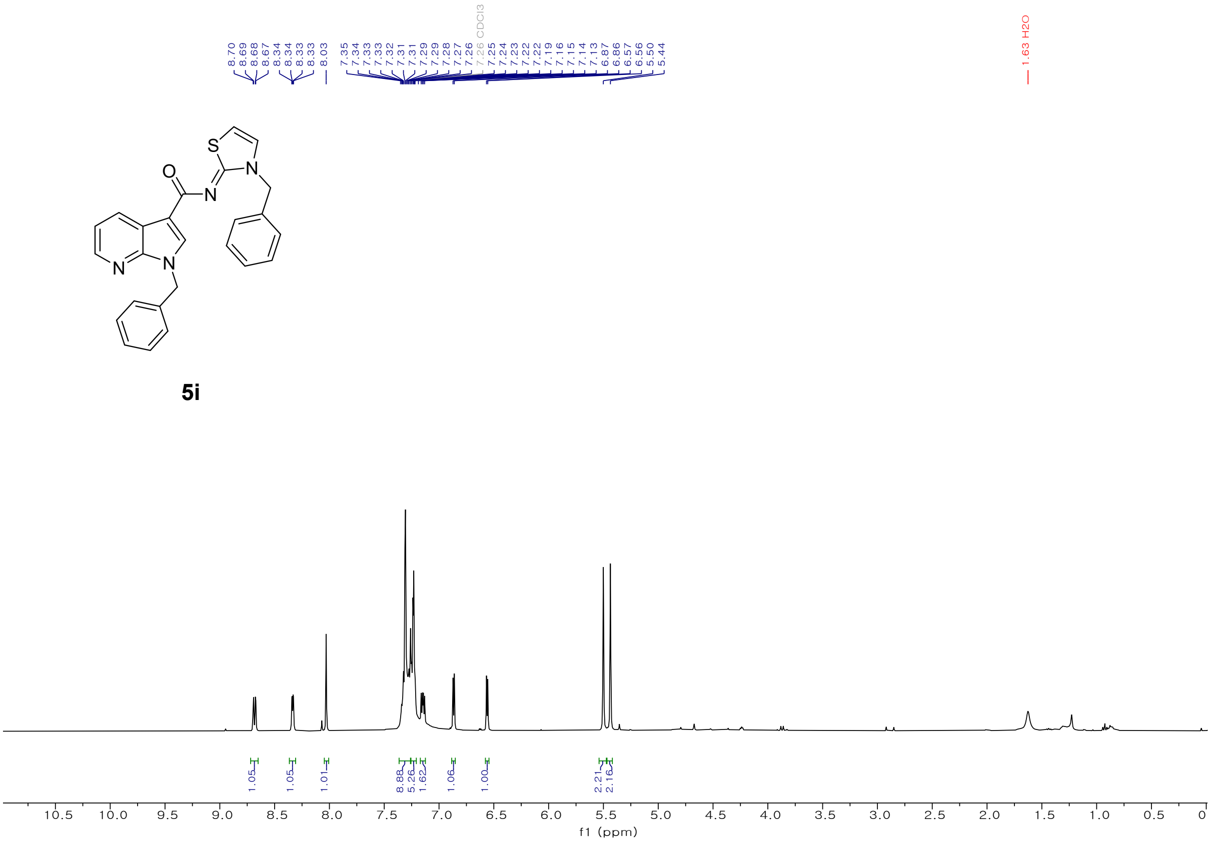

Figure S19. ^{1}H NMR of **5i** (400 MHz, $CDCl_3$).

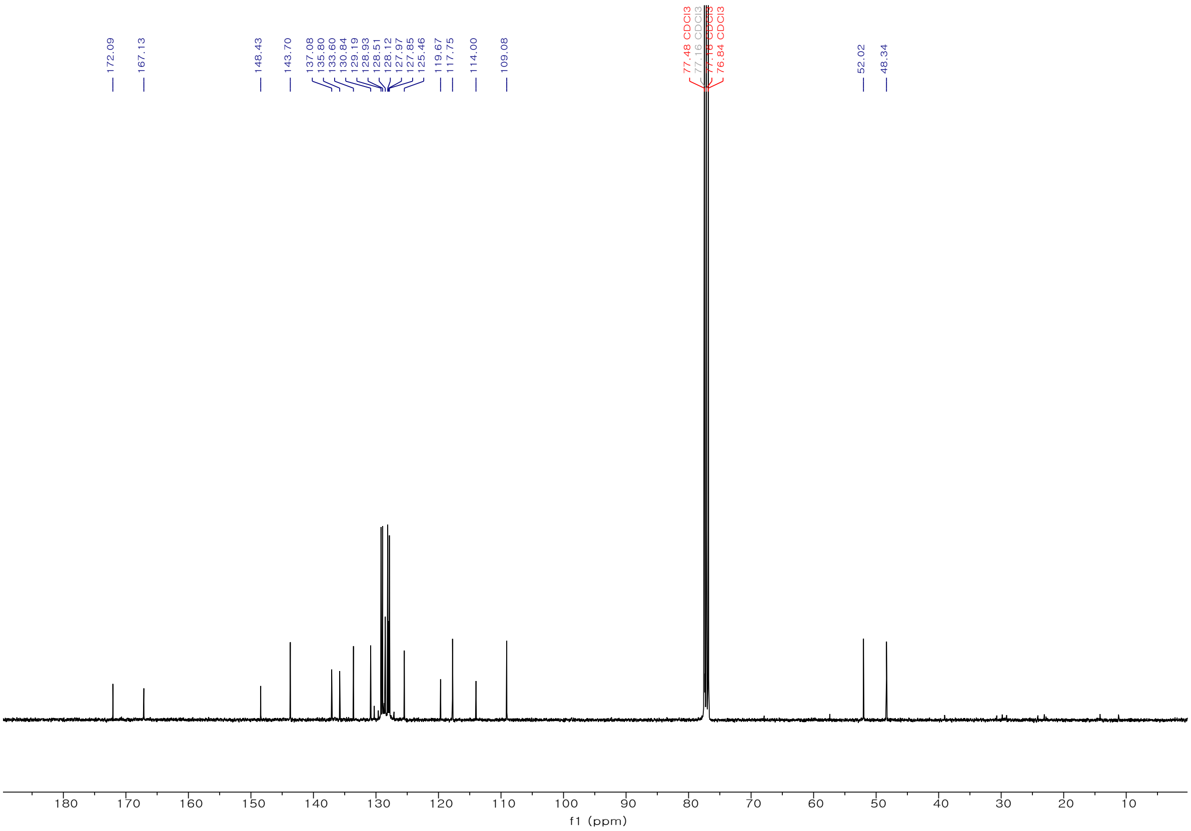

Figure S20. ^{13}C NMR of **5i** (101 MHz, $CDCl_3$).

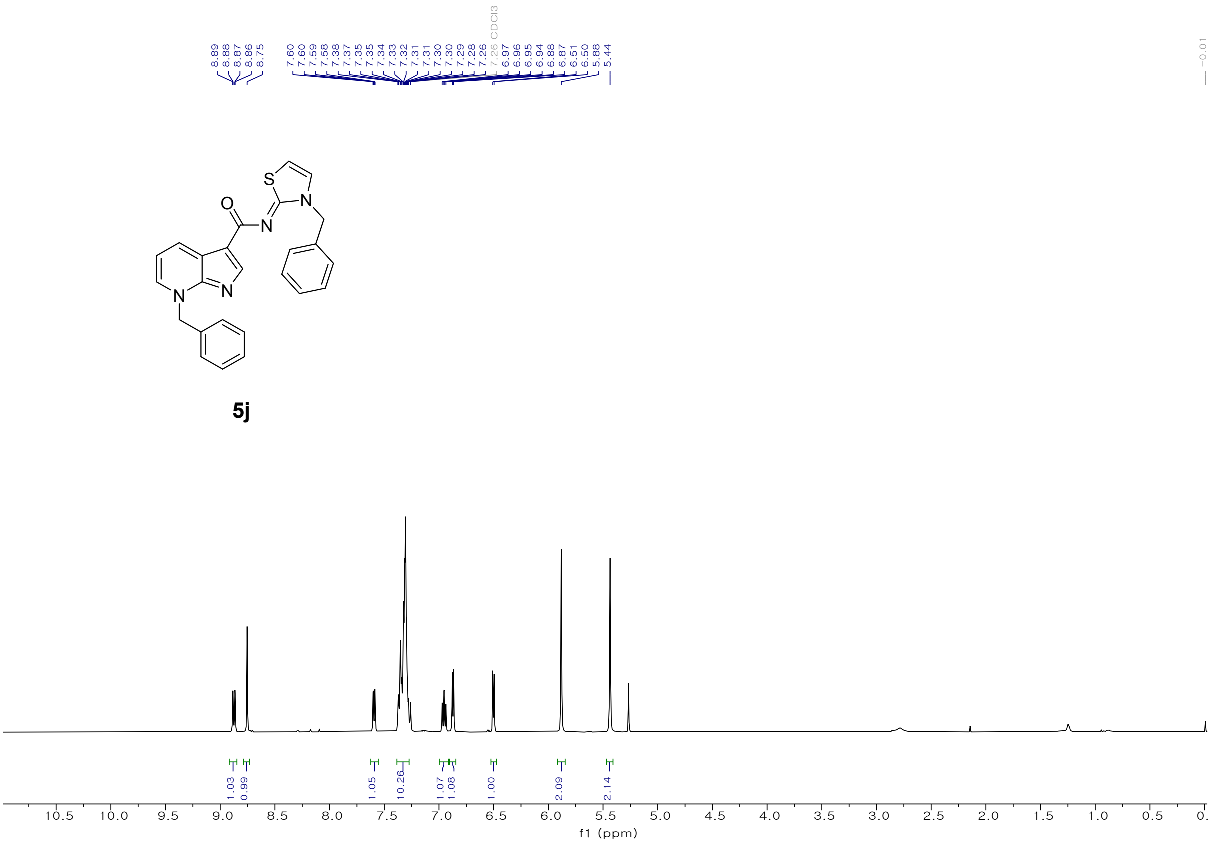

Figure S21. ^{1}H NMR of **5j** (400 MHz, $CDCl_3$).

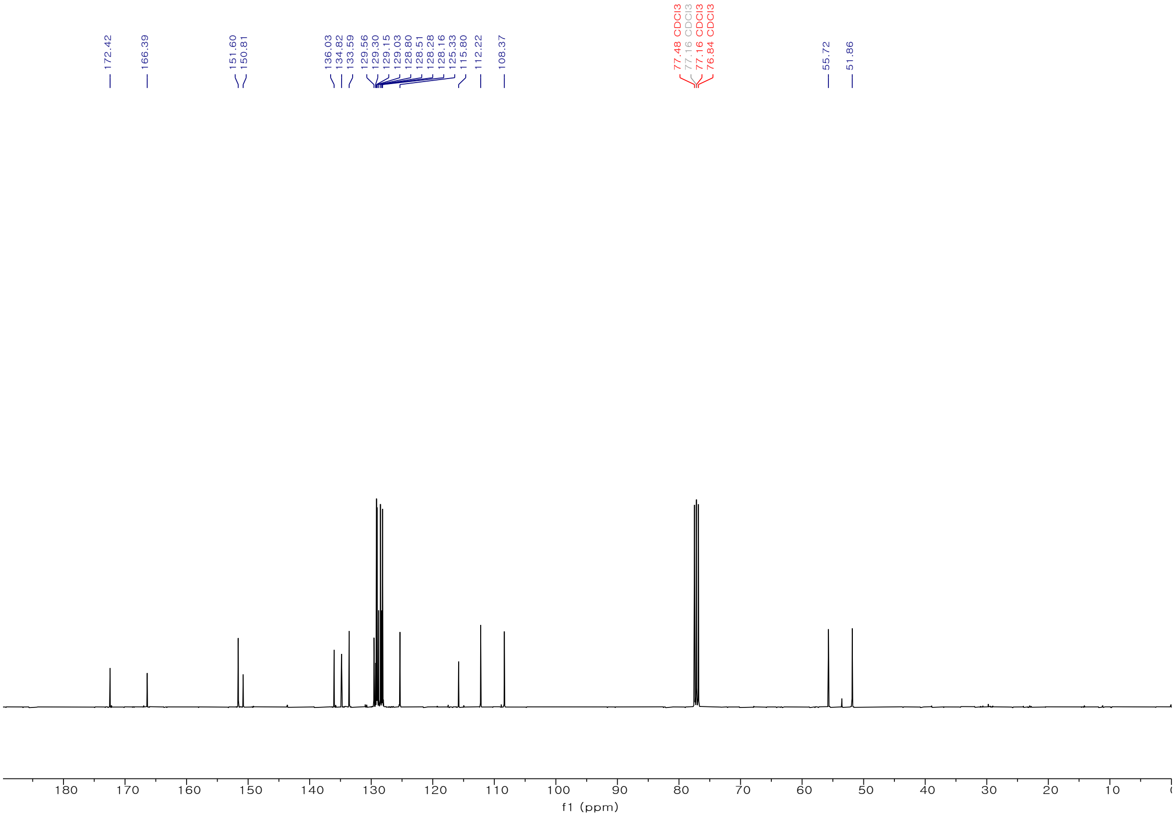

Figure S22. ^{13}C NMR of **5j** (101 MHz, $CDCl_3$).

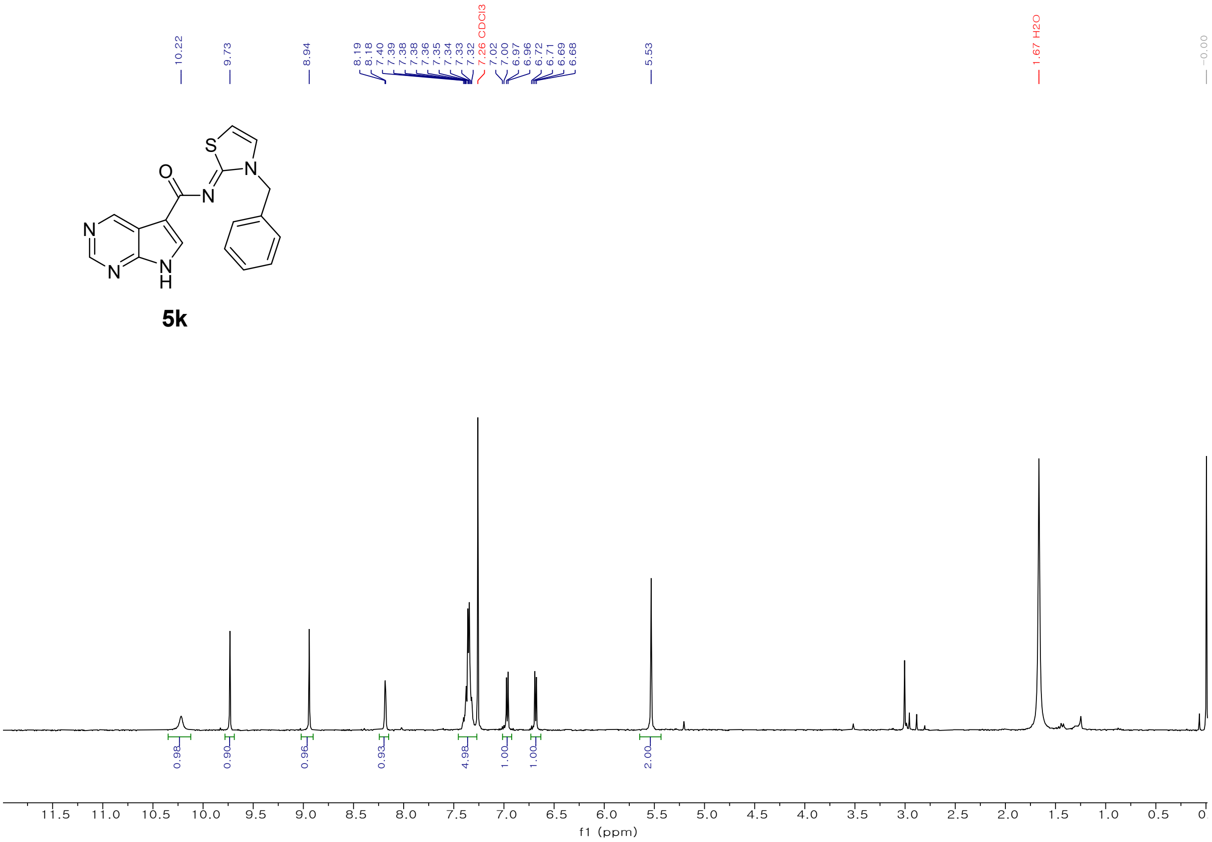

Figure S23. ^{1}H NMR of **5k** (300 MHz, $CDCl_3$).

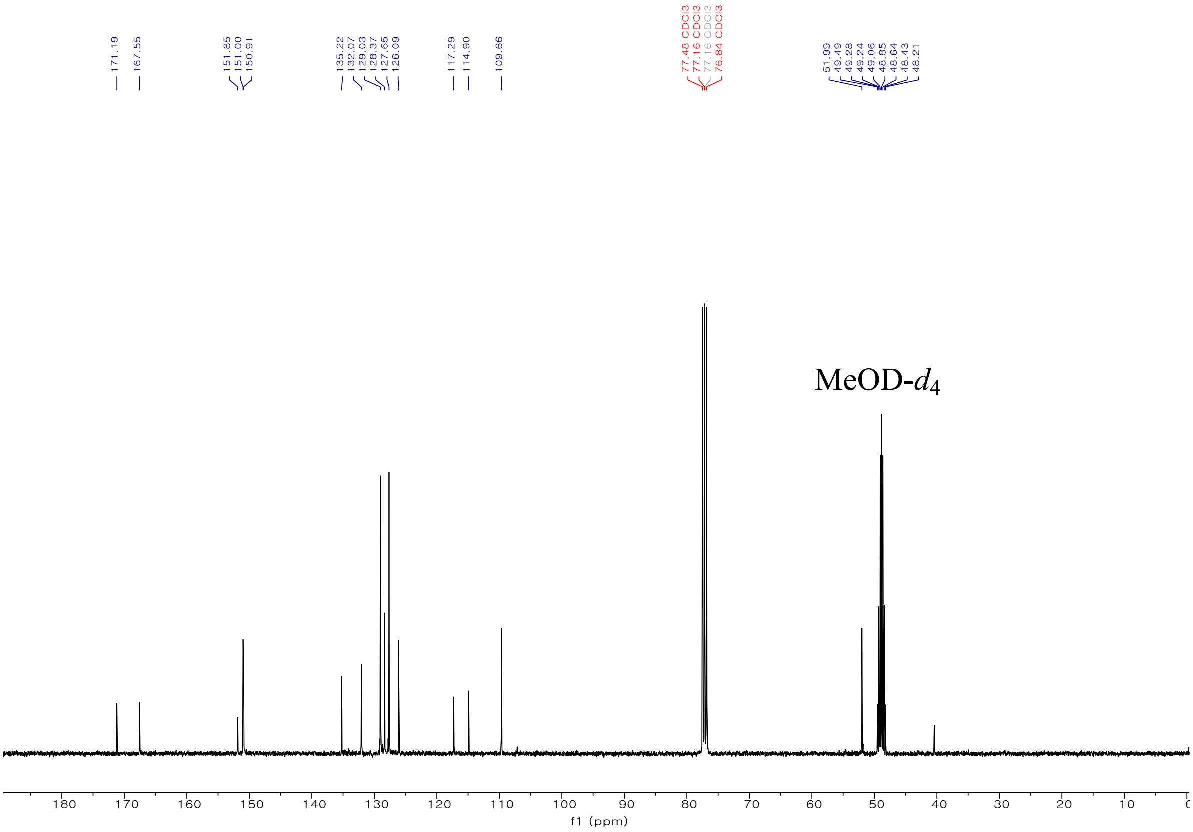

Figure S24. ^{13}C NMR of **5k** (101 MHz, $CDCl_3$+MeOD-d_4).

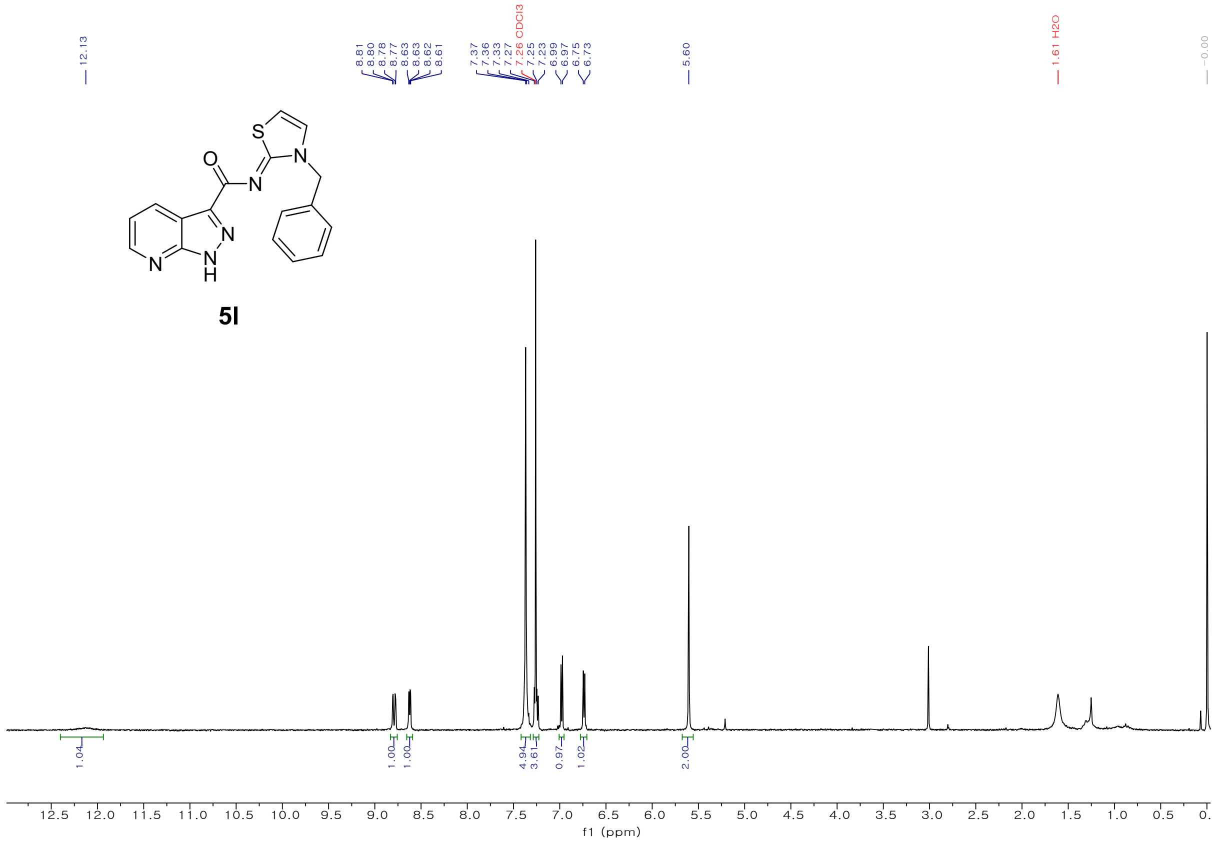

Figure S25. ^{1}H NMR of **5l** (300 MHz, $CDCl_3$).

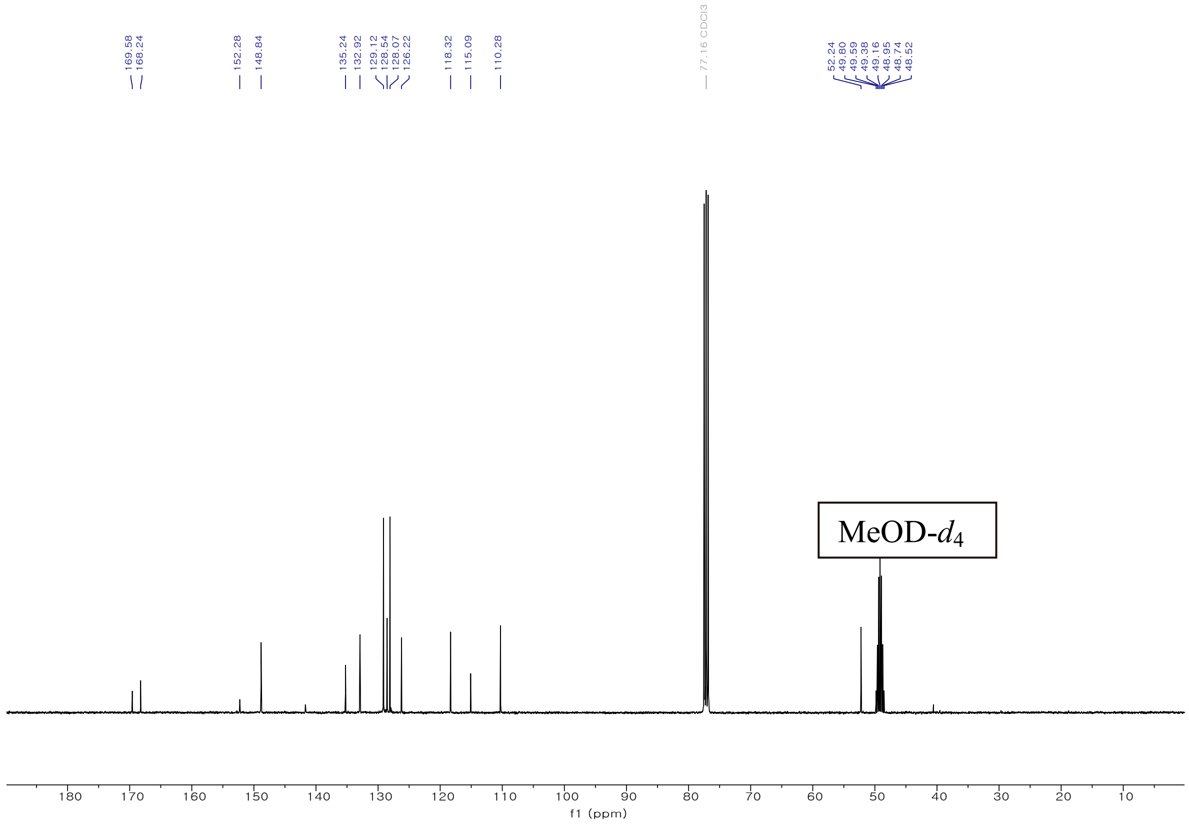

Figure S26. ^{13}C NMR of **5l** (101 MHz, $CDCl_3$+MeOD-d_4).

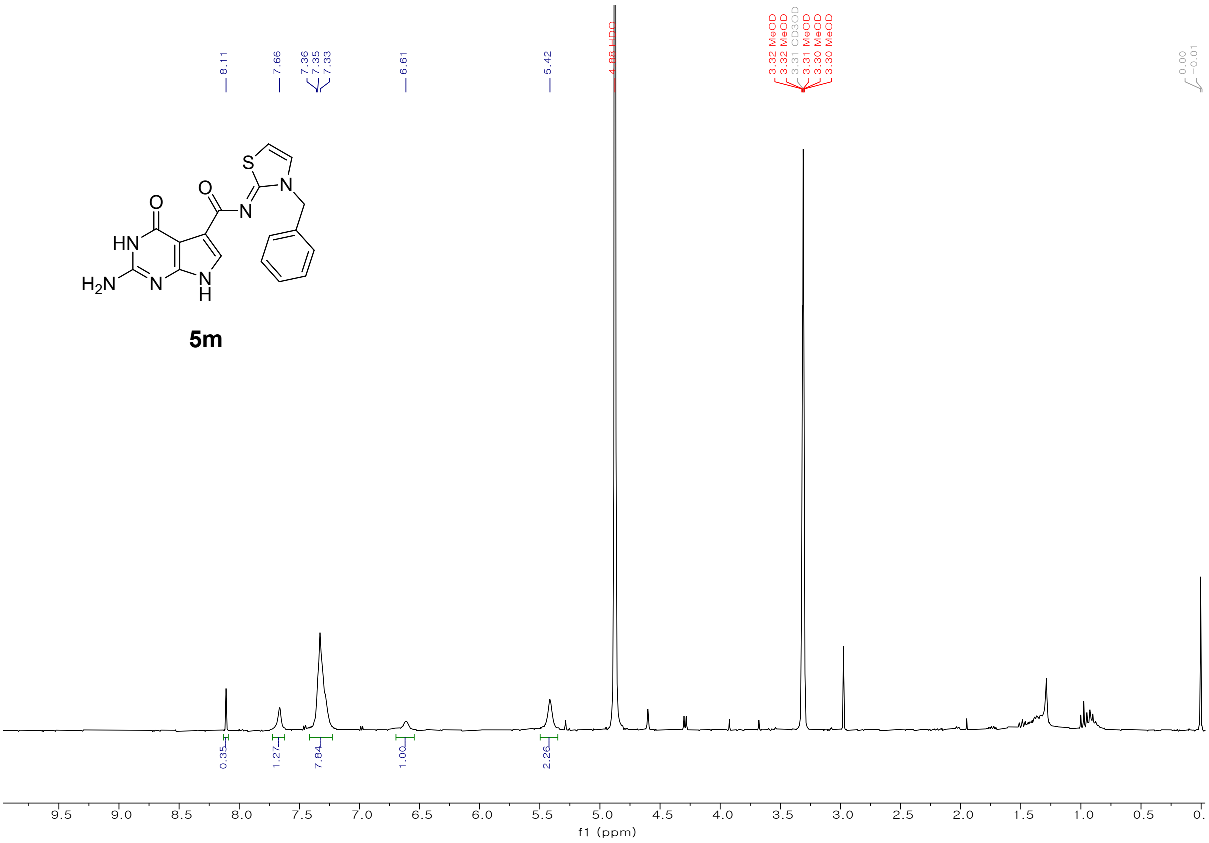

Figure S27. ^{1}H NMR of **5m** (300 MHz, MeOD-d_4).

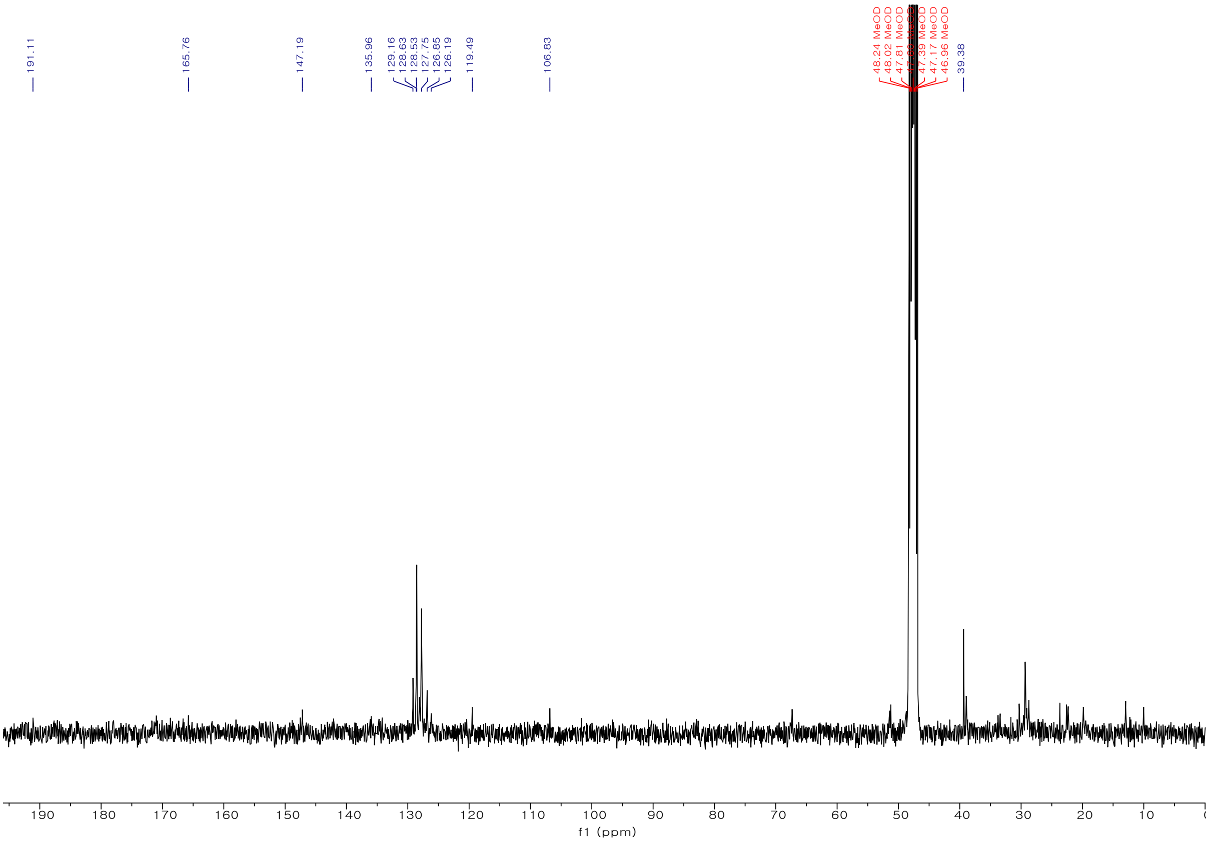

Figure S28. ^{13}C NMR of **5m** (101 MHz, MeOD-d_4).

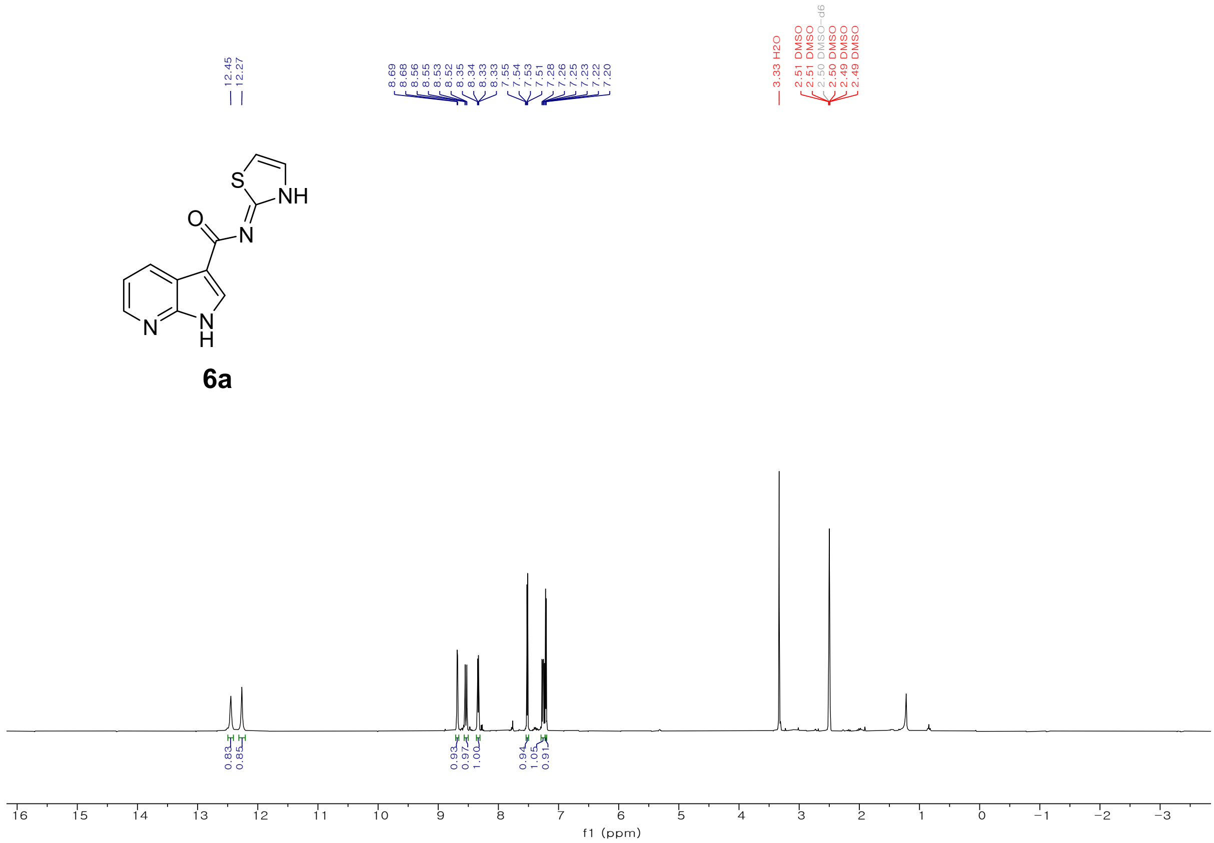

Figure S29. ^{1}H NMR of **6a** (300 MHz, DMSO-d_6).

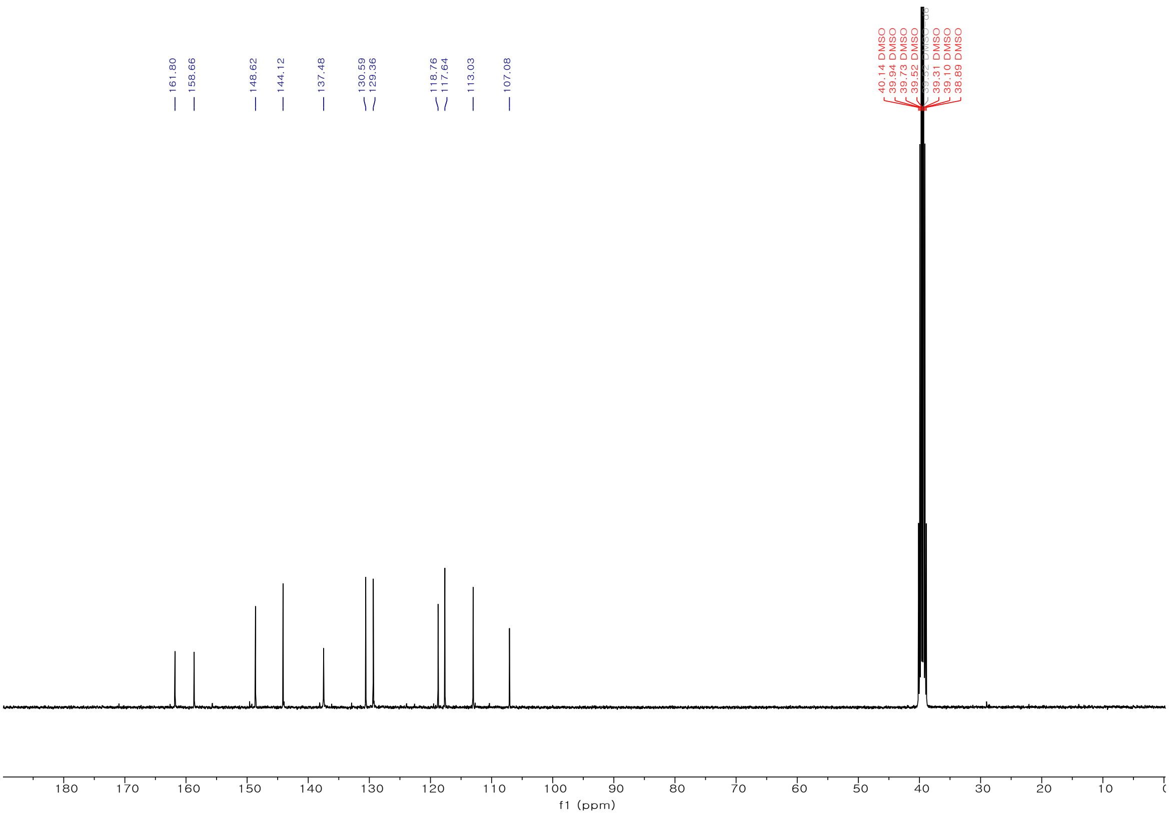

Figure S30. ^{13}C NMR of **6a** (101 MHz, DMSO-d_6).

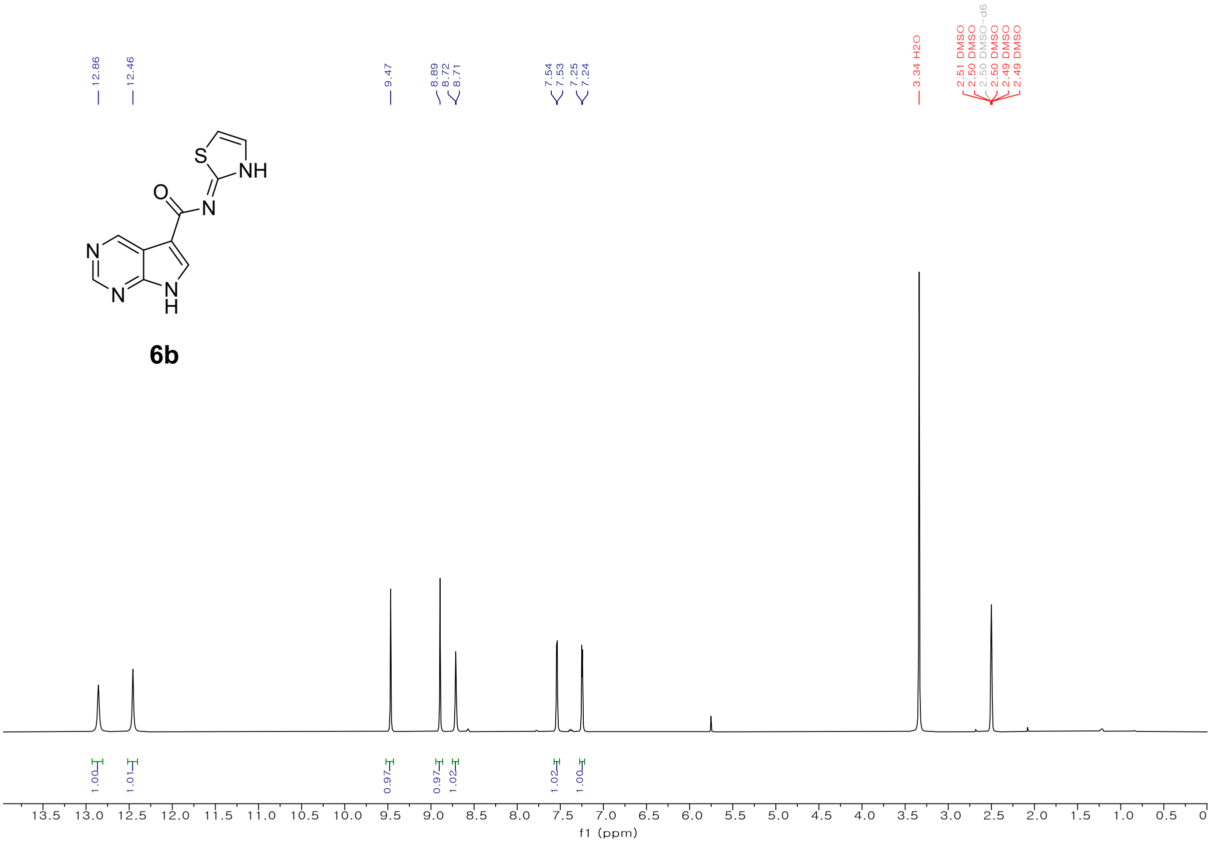

Figure S31. ¹H NMR of **6b** (400 MHz, DMSO-d_6).

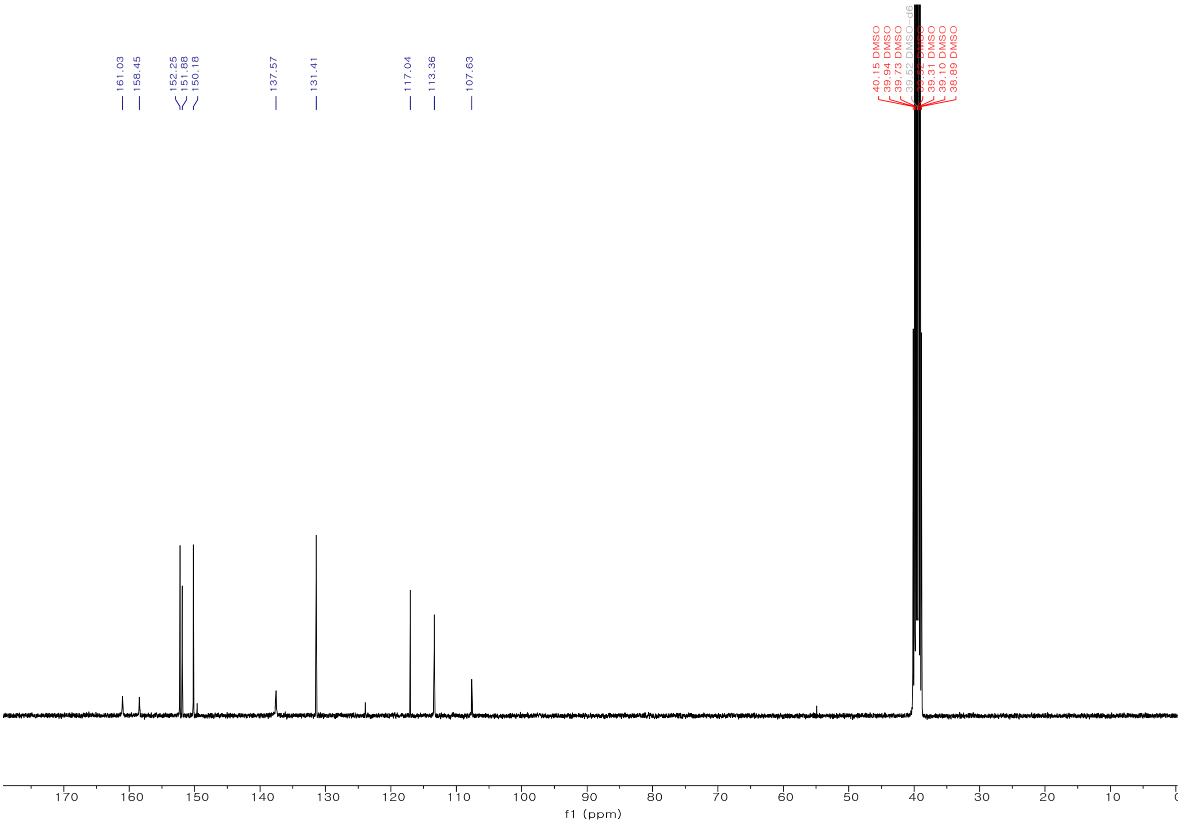

Figure S32. ^{13}C NMR of **6b** (101 MHz, DMSO-d_6).

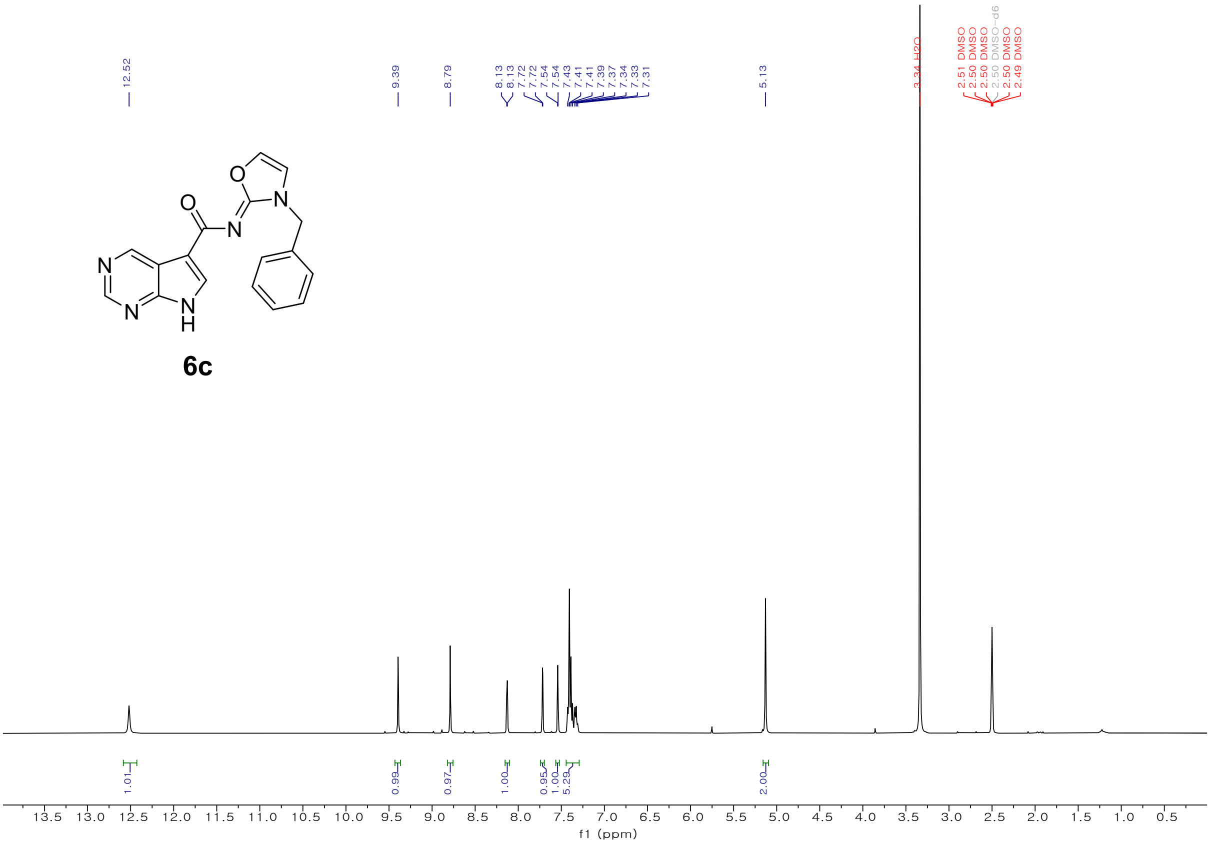

Figure S33. ¹H NMR of **6c** (400 MHz, DMSO-d_6).

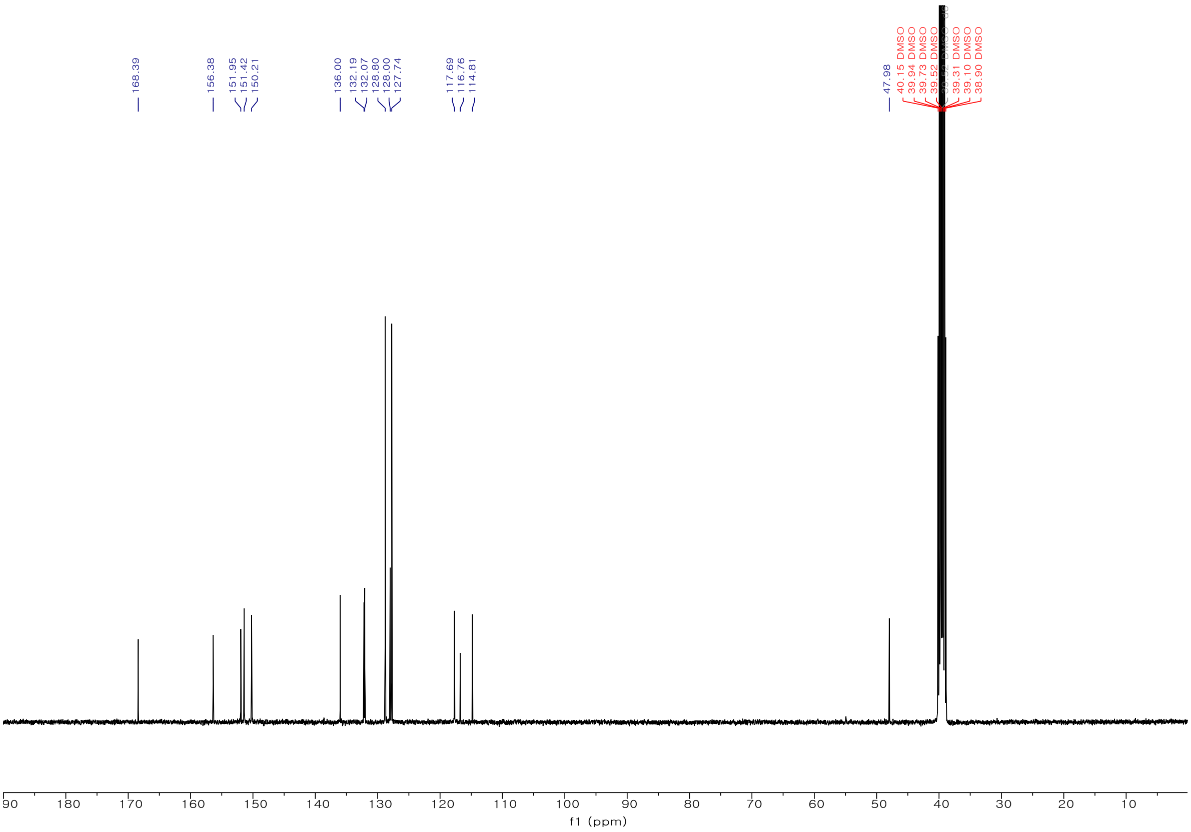

Figure S34. ^{13}C NMR of **6c** (101 MHz, DMSO-d_6).

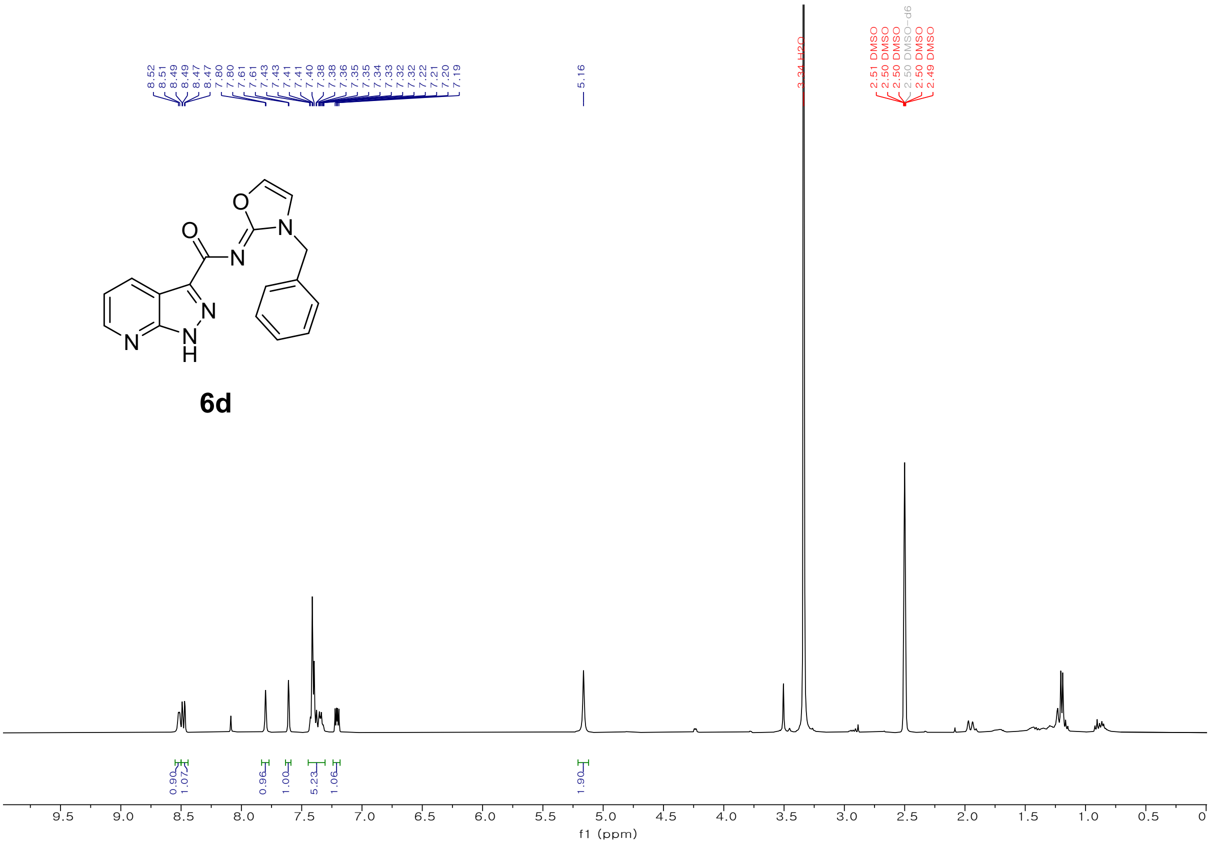

Figure S35. ^{1}H NMR of **6d** (400 MHz, DMSO-d_6).

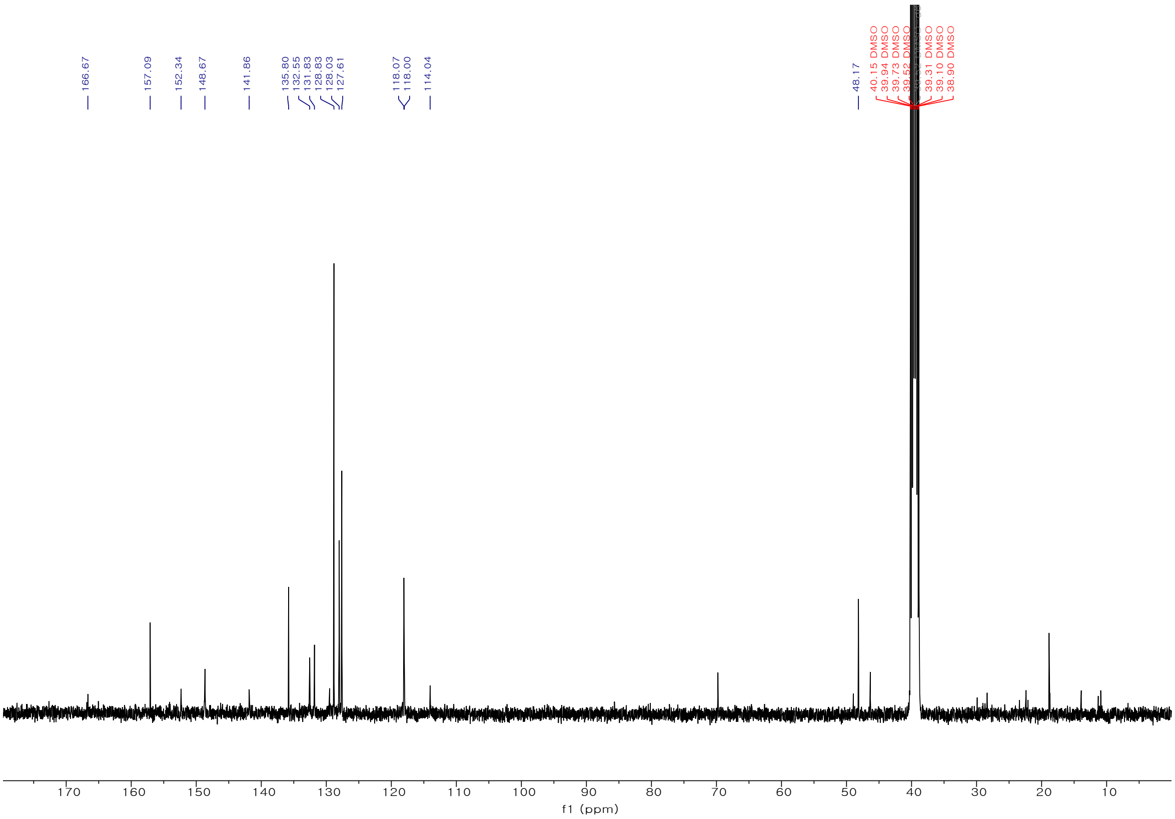

Figure S36. ^{13}C NMR of **6d** (101 MHz, DMSO-d_6).

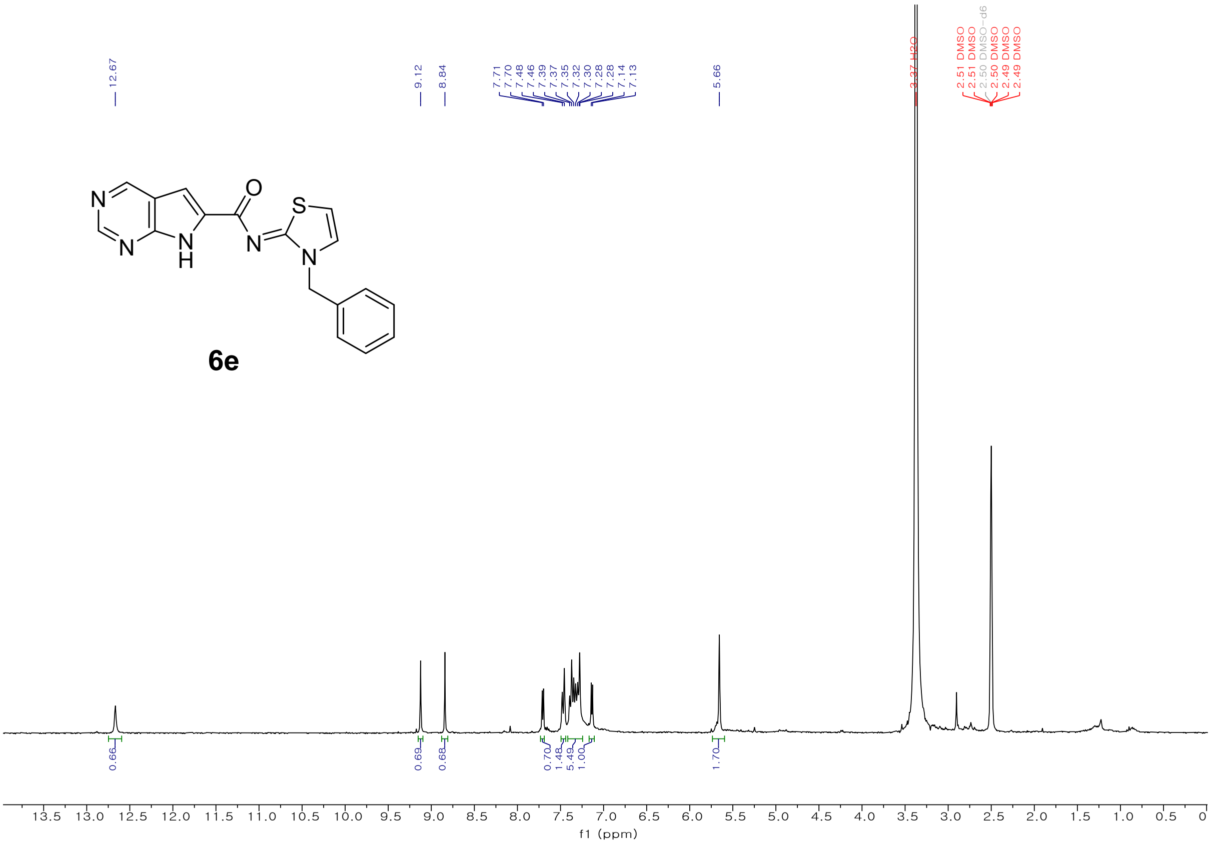

Figure S37. ^{1}H NMR of **6e** (300 MHz, DMSO-d_6).

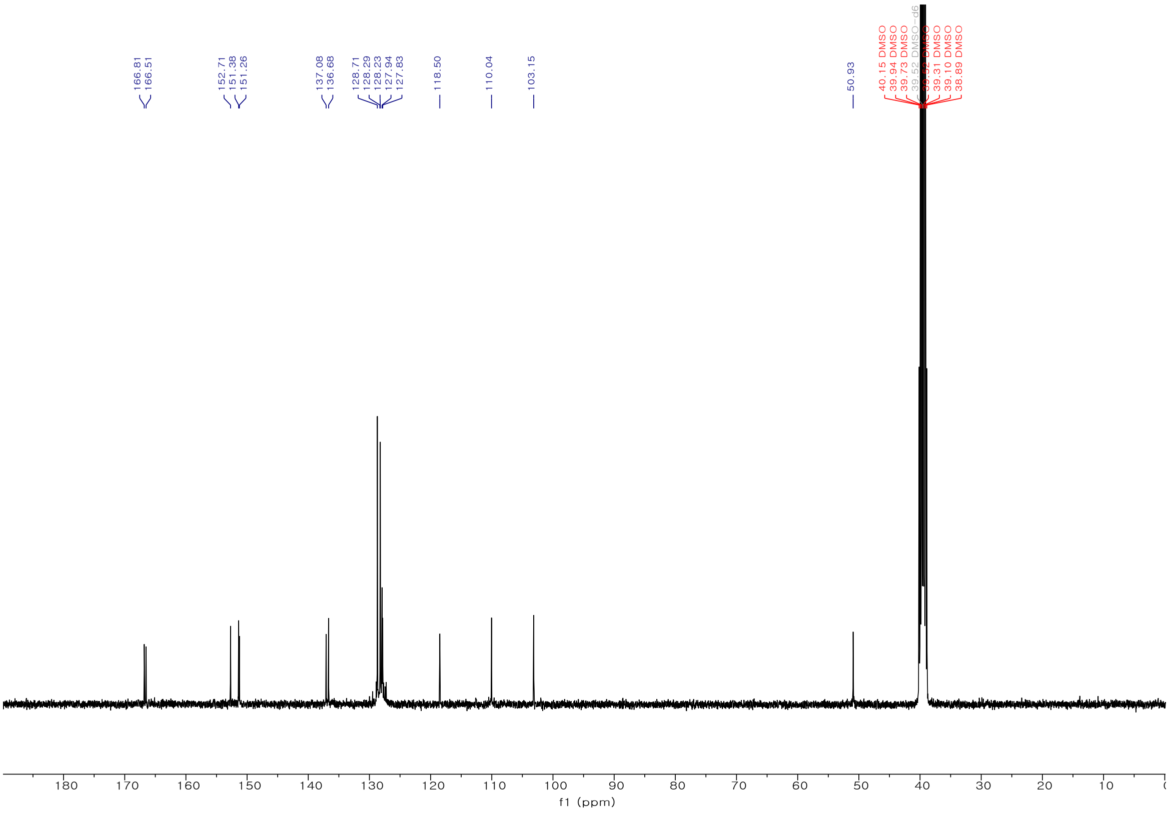

Figure S38. ^{13}C NMR of **6e** (101 MHz, DMSO-d_6)

3. In vitro kinase activity assay

The Eurofins Kinase Profiler service was requested and used for obtaining in vitro kinase activity data. Kinase inhibition was determined at 1 µM compound concentration and at 90 µM ATP concentration for LATS1(h) and at 155 µM for LATS2(h) kinases for compounds 1, 5a-m and 6a-e. For LATS1(h) and LATS2(h), the selected kinase was incubated with 8 mM MOPS pH 7.0, 0.2 mM EDTA, 250 µM KKLNRTLSFAEPG, 10 mM Magnesium acetate and [γ-33P-ATP] (specific activity and concentration as required). The reac MeOD-d_4 d by the addition of the Mg/ATP mix. After incubation for 40 minutes at room temperature, the reaction is stopped by the addition of phosphoric acid to a concentration of 0.5%. 10 µl of the stopped reaction is spotted onto a P30 filtermat and washed four times for 4 minutes in 0.425% phosphoric acid and once in methanol prior to drying and scintillation counting. The values represent the average of two independent experiments. The IC_{50} data for compounds 1, 5k, and 5l was also determined using the Eurofins IC50 Profiler service at 90 µM ATP concentration for LATS1(h) and at 155 µM for LATS2(h) kinases. The IC_{50} curves (refer to Figures S39-S41) were generated using 9 test compound concentrations, diluted in half-log increments from

10 μM to 1 nM, alongside vehicle control wells. The values represent the mean of two independent experiments.

Kinase: LATS1(h)
ATP Concentration: 90 μM
Compound concentration: 1 μM

Table S1. In vitro kinase activity for LATS1

Compound	Enzymatic activity (% Control)	Mean±SD
**1** (Truli)	3	3±1
	2	
**5a**	106	106±0
	106	
**5b**	135	134±2
	132	
**5c**	156	145±15
	134	
**5d**	104	103±2
	101	
**5e**	120	114±10
	107	
**5f**	110	111±1
	112	
**5g**	107	106±2
	104	
**5h**	104	104±0
	104	
**5i**	96	94±4
	91	
**5j**	64	57±10
	50	

**5k**	19	21±2
	22	
**5l**	8	8±0
	8	
**5m**	102	99±4
	96	
**6a**	84	82±4
	79	
**6b**	103	100±4
	97	
**6c**	50	52±2
	53	
**6d**	32	30±3
	28	
**6e**	96	96±0
	96	

Kinase: LATS2(h)

ATP Concentration: 155 µM

Compound concentration: 1 µM

Table S2. In vitro kinase activity for LATS2

Compound	Enzymatic activity (% Control)	Mean± SD
**1** (Truli)	0	1±1
	2	
**5a**	98	101±4
	104	
**5b**	109	109±1
	108	
**5c**	87	92±7
	97	

**5d**	90	90±0
	89	
**5e**	103	109±7
	114	
**5f**	107	102±7
	97	
**5g**	83	86±4
	89	
**5h**	104	104±0
	104	
**5i**	55	55±1
	54	
**5j**	25	23±3
	20	
**5k**	22	21±2
	19	
**5l**	0	1±1
	1	
**5m**	112	111±2
	109	
**6a**	80	78±4
	75	
**6b**	106	103±4
	100	
**6c**	59	61±2
	62	
**6d**	30	28±3
	26	
**6e**	108	107±2
	105	

Table S3. Estimated IC_{50} values of **1**, **5k** and **5l**

Compound	Kinase	IC_{50} (nM)
**1** (Truli)	LATS1(h)	22
**1** (Truli)	LATS2(h)	6
**5k**	LATS1(h)	265
**5k**	LATS2(h)	395
**5l**	LATS1(h)	43
**5l**	LATS2(h)	24

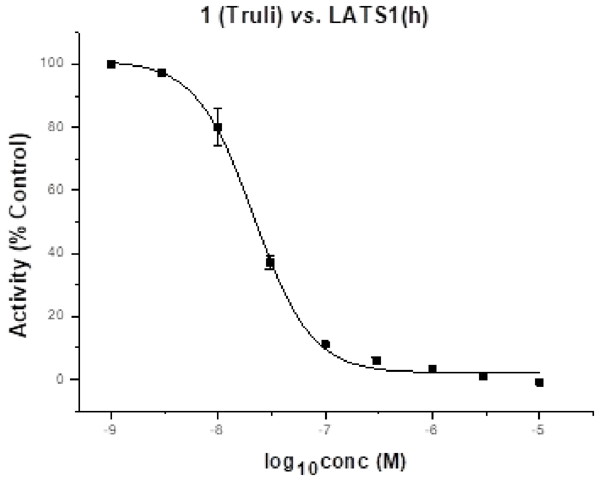

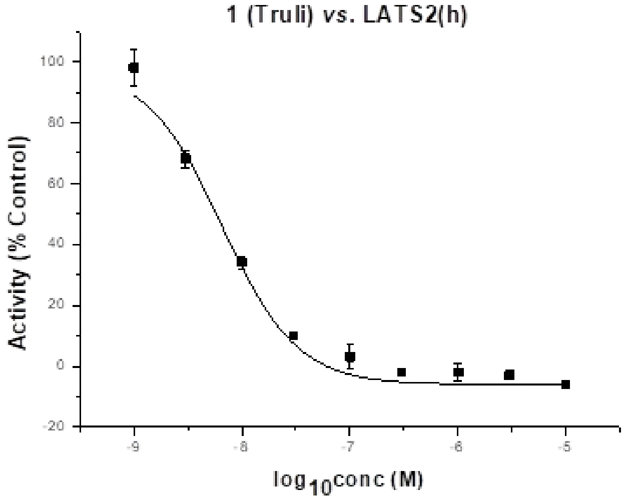

Figure S39. Estimated IC_{50} values of **1** (Truli) measured at 90 μM ATP concentration for LATS1(h) and at 155 μM ATP concentration for LATS2(h).

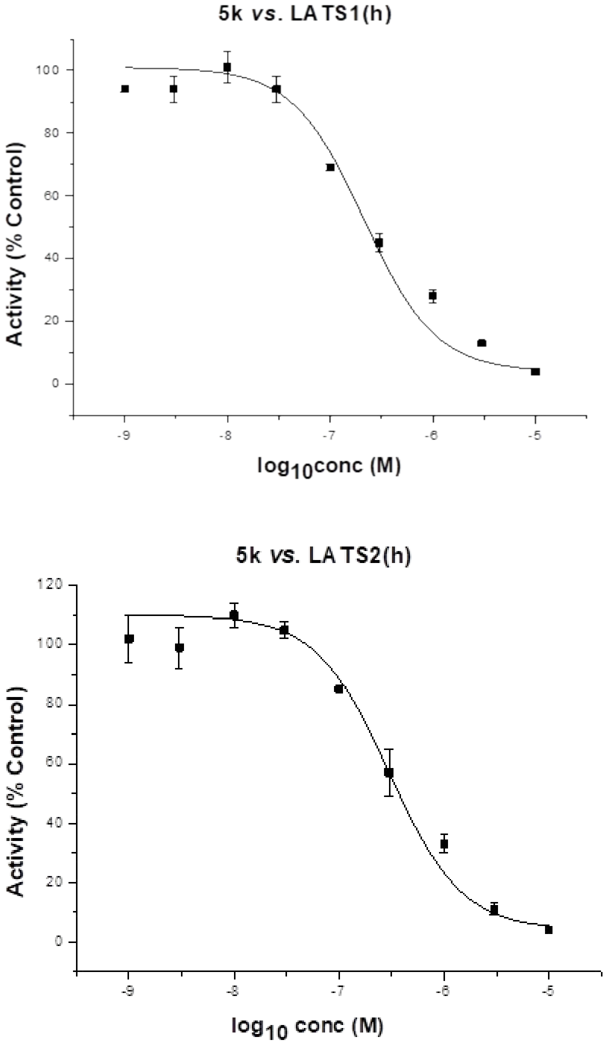

Figure S40. Estimated IC_{50} values of **5k** measured at 90 µM ATP concentration for LATS1(h) and at 155 µM ATP concentration for LATS2(h).

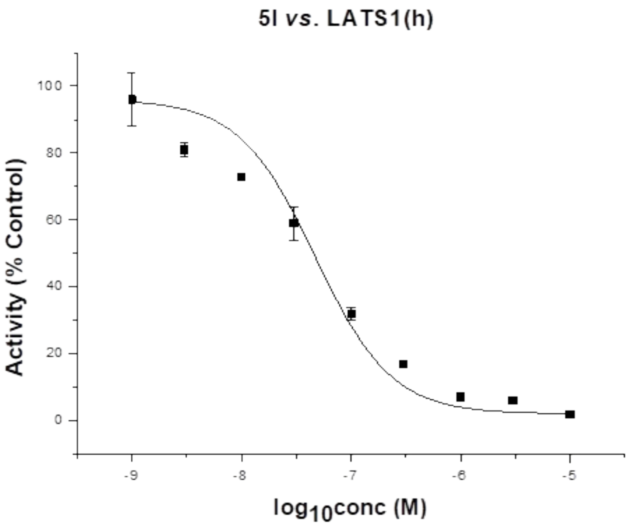

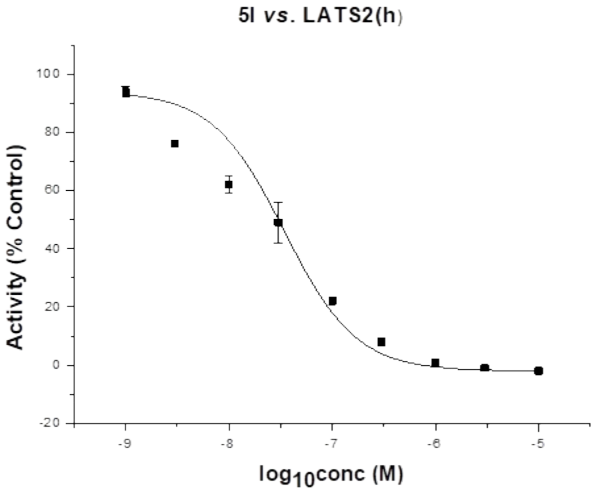

Figure S41. Estimated IC_{50} values of **5l** measured at 90 μM ATP concentration for LATS1(h) and at 155 μM ATP concentration for LATS2(h).

4. Solubility[3]

3 mg of each compound was weighed into 1.5 mL Eppi tubes. 700 μL of D_2O (deuterium oxide) was then added and the sample was sonicated for 5 min and was subjected to shaking on a high-speed vibrating mixer for 24 h at rt. After, sample was centrifuged at 10,000 rpm for 5 min. For analysis, the supernatant was filtered using 0.45 μm PVDF syringe filter. For the quantitation, DMSO (dimethyl sulfoxide) was used as an internal standard. The concentration of each compound in D_2O was calculated based on the integration ratio of the compound signal to the internal standard DMSO signal (2.71 ppm, 6H) (n=3).

5. Metabolic stability[4,5]

Microsomes diluted with Potassium phosphate buffer were incubated at 37°C for 5 minutes, then the tested compound and NADPH were added and reacted at 37°C for 30 minutes (test compound final conc.: 1μM, microsome final conc.: 0.5 mg/mL). To terminate the reaction, cold acetonitrile containing an internal standard was added and then treated with deproteinization. After centrifugation (4,000 rpm, 4°C, 15 min), the supernatant was analyzed by LC-MS/MS (Mass spectrometry (Agilent 6460) with HPLC (Agilent 1260)).

Table S4. Liver microsomal phase I stability (% of remaining after 30 min) (mean ± SD, n=3)

Compound	Mouse (%)	Human (%)
**1** (Truli)	0.18 ± 0.03	2.68 ± 0.46
**5k**	13.81 ± 0.55	43.10 ± 1.10
**5l**	0.69 ± 0.17	34.92 ± 1.31

6. Mouse pharmacokinetics[6]

Pharmacokinetic profiles of **5k** and **5l** were obtained using male mice. Blood was centrifuged to separate plasma, and 9x the volume of cold acetonitrile containing an internal standard is added, followed by deproteinization. After centrifugation (13,000 rpm, 4 °C, 10 min), the supernatant was analyzed by LC-MS/MS (Mass spectrometry (Agilent 6460) with HPLC (Agilent 1260)). For the calibration curve of the compound, a 10x higher concentration solution (0.5-8000 ng/mL) was prepared by adding it to blank plasma, and it was further prepared in the same manner as the tested compound.

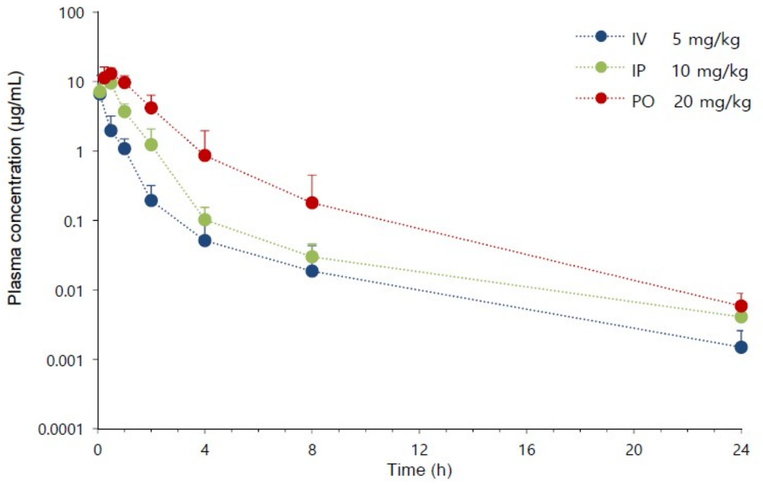

Figure S42. Plasma concentration-time profiles of **5k** in male mice (n=3).

Table S5. Pharmacokinetic parameters of **5k** in male mice.

Parameter	IV, 5 mg/kg	IP, 10 mg/kg	PO, 20 mg/kg
T_{max} (h)	NA	0.5 ± 0	0.42 ± 0.14
C_{max} (μg/mL)	NA	9.51 ± 2.03	13.32 ± 2.82
$T_{1/2}$ (h)	2.86 ± 1.88	5.24 ± 2.45	4.25 ± 3.57
AUC_{last} (μg*h/mL)	4.34 ± 2.17	11.42 ± 3.28	25.71 ± 8.01
AUC_{∞}(μg*h/mL)	4.34 ± 2.16	11.46 ± 3.25	25.75 ± 7.97
CL (L/h/kg)	1.41 ± 0.82	NA	NA
V_{ss} (L/kg)	3 ± 4.42	NA	NA
MRT_{last} (h)	1.43 ± 1.65	1.14 ± 0.15	1.74 ± 0.99
MRT_{∞} (h)	1.49 ± 1.72	1.27 ± 0.15	1.81 ± 0.94
F_t (%)	NA	131.71	148.23

NA, not applicable; ND, not detected; NC, not calculated

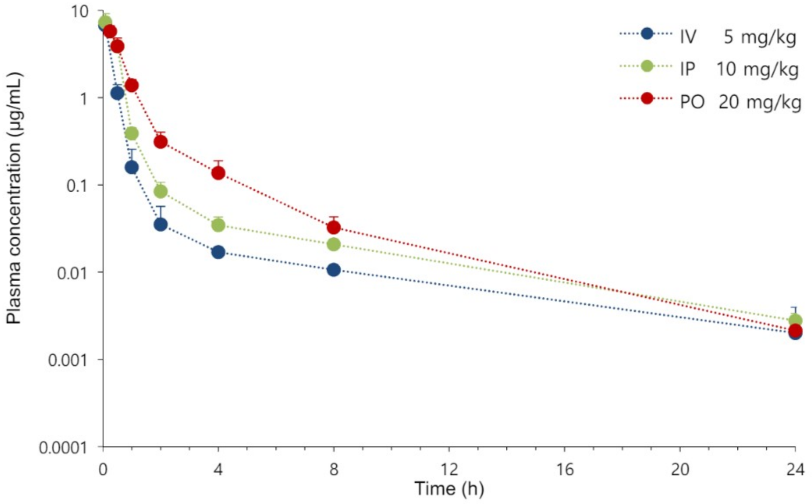

Figure S43. Plasma concentration-time profiles of **5l** in male mice (n=3).

Table S6. Pharmacokinetic parameters of **5l** in male mice.

Parameter	IV, 5 mg/kg	IP, 10 mg/kg	PO, 20 mg/kg
T_{max} (h)	NA	0.08 ± 0.00	0.25 ± 0.00
C_{max} (μg/mL)	NA	7.30 ± 1.88	5.74 ± 0.92
$T_{1/2}$ (h)	6.56 ± 3.32	5.59 ± 0.88	3.46 ± 0.33

AUC_{last} (μg*h/mL)	2.94 ± 0.18	4.37 ± 0.72	5.15 ± 0.59
AUC_{∞}(μg*h/mL)	2.96 ± 0.15	4.39 ± 0.71	5.16 ± 0.59
CL (L/h/kg)	1.69 ± 0.09	NA	NA
V_{ss} (L/kg)	1.80 ± 1.32	NA	NA
MRT_{last} (h)	0.71 ± 0.25	0.97 ± 0.12	1.50 ± 0.10
MRT_{∞} (h)	1.04 ± 0.71	1.14 ± 0.21	1.56 ± 0.10
F_t (%)	NA	74.36	43.89

NA, not applicable; ND, not detected; NC, not calculated

7. Kinase screening results

A set of 468 kinase inhibitory tests was performed on the compound **5l** at a concentration of 100 nM by using the scanMAX kinase assay panel of KINOMEscan. In this study, the kinase binding maps of **5l** demonstrated the strength and relative specificity of kinase-binding interactions. Results are reported as the percentage of the control (%Ctrl), where %Ctrl = [(positive control signal - test compound signal)/(positive control signal - negative control signal)]*100. Dimethyl sulfoxide (DMSO) was used as the negative control. Lower values of %Ctrl indicate a stronger interaction between **5l** and kinases. TREE*spot* was generated online using the TREE*spot*™ software tool. A large red circle indicates higher-affinity binding of numerous kinases.

Table S7. Target enzymes binding more potently than LATS1 and LATS2. Of 468 kinases in a binding panel, these enzymes bound 100 nM **5l** compound relatively more strongly than LATS1 or LATS2 did.

KINOMEscan Gene Symbol	%Ctrl @ 100 nM*
LATS1	92
LATS2	27
DMPK	6.5
FLT3(D835V)	16
YSK4	7.1

*Data shown are the relative binding (% to control) of each kinase to its respective ligand with no competitor added in the presence of 100 nM **5l**.

Selectivity Score (S-scores)

Selectivity Score or S-score is a quantitative measure of compound selectivity. It is calculated by dividing the number of kinases that compounds bind to by the total number of distinct kinases tested, excluding mutant kinases.

S = Number of hits / Number of assays

This value can be calculated using %Ctrl as a potency threshold (below) and provides a quant itative method of describing compound selectivity to facilitate comparison of different compounds.

S(35) = (number of non-mutant kinases with %Ctrl <35)/(number of non-mutant kinases tested)

S(10) = (number of non-mutant kinases with %Ctrl <10)/(number of non-mutant kinases tested)

S(1) = (number of non-mutant kinases with %Ctrl <1)/(number of non-mutant kinases tested)

Table S8. S-score values

Compound name	Selectivity score type	Number hits	Number of non-mutant kinases	Screening conc. (nM)	Selectivity score
**5l**	S(35)	3	403	100	0.007
**5l**	S(10)	2	403	100	0.005
**5l**	S(1)	0	403	100	0

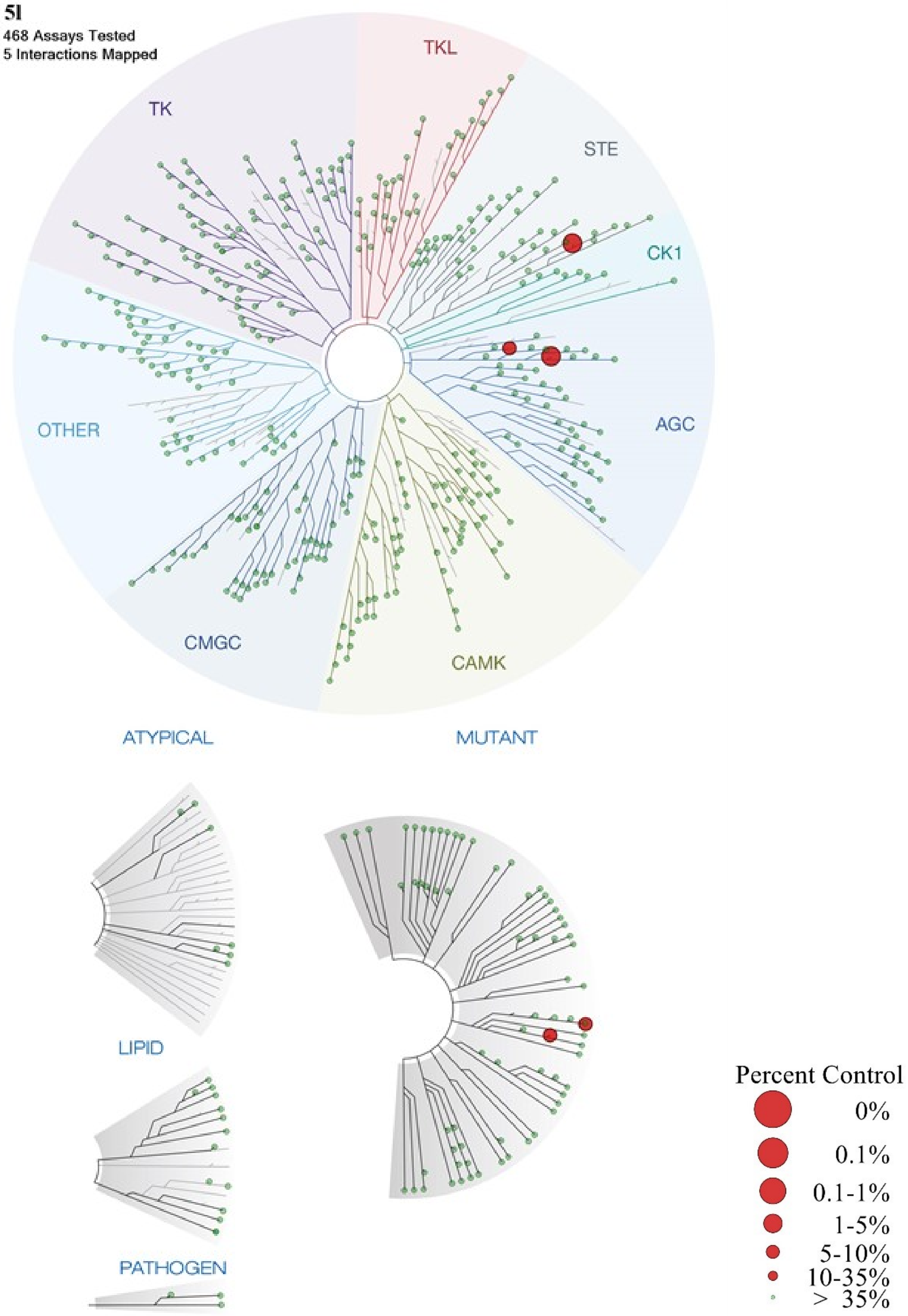

Figure S44. The TREEspot compound profile of **5l**.

8. KRICT-AI assisted prediction of metabolic stability

KRICT-AI (pre-trained machine learning model, PredMS)[7] platform was accessed online via https://predms.netlify.app/ and used to get predicted values of metabolic stabilities of the analyzed compounds **1**, **5a-m** and **6a-e**. PredMS predicts metabolic stability for a given compound as stable (≥ 50% remaining at 30 min) or unstable (< 50% remaining at 30 min) in human liver microsomes. The chemical structures of the compounds were presented in the simplified molecular-input line-entry system (SMILES) format and were further submitted for evaluation.

Table S9. KRICT-AI assisted prediction data

**Compound**	**Metabolic stability**
**1** (Truli)	Human: Unstable (0.457)
**2** (NIBR-LTSi)	Human: Stable (0.646)
**3** (GA-017)	Human: Stable (0.799)
**5a**	Human: Unstable (0.363)
**5b**	Human: Unstable (0.428)
**5c**	Human: Unstable (0.428)
**5d**	Human: Unstable (0.403)
**5e**	Human: Unstable (0.480)
**5f**	Human: Unstable (0.489)
**5g**	Human: Unstable (0.282)
**5h**	Human: Unstable (0.301)
**5i**	Human: Unstable (0.292)
**5j**	Human: Unstable (0.277)
**5k**	Human: Stable (0.592)
**5l**	Human: Stable (0.561)
**5m**	Human: Stable (0.719)
**6a**	Human: Stable (0.792)
**6b**	Human: Stable (0.829)
**6c**	Human: Stable (0.601)

**6d**	Human: Stable (0.619)
**6e**	Human: Stable (0.619)

9. Molecular Docking simulation results[8,9]

The molecular docking was performed on the HyperLab (https://www.hyperlab.hits.ai/en) online platform with the homology model created from the crystal structure of kinase ROCK1 bound to azaindole thiazole inhibitor[10] as there is no known crystal structure of the LATS kinases. The X-ray crystal structure of ROCK1 kinase complex (PDB ID: 5KKS) was downloaded from the protein data bank (www.rcsb.org). The 2D structure of the analyzed compounds **1**, **5a-m** and **6a-e** were drawn using ChemDraw software. Binding energies were automatically calculated after registering the molecular structures of the analyzed compounds. It uses artificial intelligence to predict drug-protein binding energy. The stronger the prediction value (binding score), the higher the probability of showing activity. A molecule with a binding score of -10 kcal/mol is more likely to be active compared to a molecule with -8 kcal/mol.

Table S10. Summary of interaction types in protein homology modeling

**Compound name**	**Backbone interactions (AA residue, type of interaction, bond length)**	**Side chain interactions (AA residue, type of interaction, bond length)**
1 (Truli)	Met156, hbond, vdw, 3.05Å<br>Glu154, hbond, vdw, 2.8Å<br>Ile82, vdw, 3.29Å	Val137, vdw, 3.48Å<br>Ala103, vdw, 3.44Å<br>Lys105, vdw, 3.38Å<br>Asp216, vdw, 3.25Å<br>Val90, vdw, 3.54Å<br>Leu205, vdw, 3.27Å<br>Leu205, vdw, 3.48Å
**5a**	Asp216, hbond, vdw, 2.95Å	Ala103, vdw, 3.43Å<br>Val137, vdw, 3.47Å<br>Ala215, vdw, 3.49Å<br>Lys105, vdw, 3.58Å<br>Lys105, weak hbond, 3.67Å
**5b**	Asp216, hbond, vdw, 2.95Å	Ala103, vdw, 3.4Å<br>Ala215, vdw, 3.27Å<br>Ala215, vdw, 3.4Å<br>Lys105, vdw, 3.49Å<br>Lys105, weak hbond, 3.64Å
**5c**	Glu154, hbond, vdw, 3.02Å<br>Met156, vdw, 3.31Å<br>Asp202, vdw, 3.32Å	Ala103, vdw, 3.24Å<br>Leu205, vdw, 3.33Å<br>Val137, vdw, 3.49Å<br>Asp216, vdw, 3.23Å<br>Lys105, vdw, 3.31Å

		Val90, vdw, 3.45Å
**5d**	Asp216, weak hbond, 3.28Å<br>Asp216, vdw, 3.24Å	Lys105, vdw, 3.5Å<br>Met153, vdw, 3.54Å<br>Leu205, vdw, 3.41Å
**5e**	Met156, hbond, vdw, 2.99Å	Val90, vdw, 3.25Å<br>Val90, vdw, 3.32Å<br>Leu205, vdw, 3.4Å<br>Ala103, vdw, 3.36Å
**5f**	Asp216, vdw, weak hbond, 2.99Å<br>Gly83, vdw, 3.48Å<br>Glu89, vdw, 3.45Å	Ala103, vdw, 3.4Å<br>Ala215, vdw, 3.24Å<br>Val90, vdw, 3.42Å<br>Val90, vdw, 3.49Å<br>Lys105, vdw, 3.18Å
**5g**	Phe87, hbond, vdw, 2.9Å<br>Gly88, hbond, 3.19Å	Val90, vdw, 3.41Å
**5h**	Arg84, vdw, 3.24Å<br>Arg84, vdw, 2.97Å<br>Ile82, vdw, 2.76Å<br>Ile82, vdw, 3.2Å	Asp216, vdw, 2.95Å<br>Asp216, vdw, 3.49Å<br>Phe368, vdw, 3.49Å<br>Phe368, vdw, 3.47Å<br>Met153, vdw, 3.4Å<br>Val90, vdw, 3.45Å
**5i**	Asp202, vdw, 3.36Å<br>Asp216, vdw, weak hbond, 3.12Å<br>Ile82, vdw, 3.03Å<br>Ile82, vdw, 2.99Å	Met153, vdw, 3.41Å<br>Asp150, vdw, 3.19Å<br>Phe368, vdw, 3.48Å<br>Phe368, vdw, 3.44Å
**5j**	-	Val90, vdw, 3.27Å<br>Phe87, vdw, 3.47Å
**5k**	Glu154, hbond, vdw, 3.15Å<br>Met156, hbond, vdw, 2.96Å	Asp216, vdw, 3.49Å<br>Ala215, vdw, 3.59Å<br>Val90, vdw, 3.46Å<br>Val90, vdw, 3.19Å<br>Ala103, vdw, 3.48Å<br>Leu205, vdw, 3.5Å
**5l**	Glu154, hbond, vdw, 2.81Å<br>Met156, hbond, vdw, 3.01Å<br>Ile82, vdw, 3.23Å<br>Ile82, vdw, 3.28Å	Ala103, vdw, 3.47Å<br>Leu205, vdw, 3.29Å<br>Met153, vdw, 3.4Å<br>Lys105, vdw, 3.29Å<br>Asp216, vdw, 3.25Å
**5m**	Glu154, vdw, weak hbond, 2.8Å<br>Asp216, hbond, 3.25Å<br>Arg84, vdw, 3.27Å<br>Arg84, vdw, 3.06Å	Met153, vdw, weak hbond, 3.4Å<br>Ala215, vdw, 3.27Å<br>Leu205, vdw, 3.49Å
**6a**	Glu154, hbond, vdw, 2.88Å<br>Met156, hbond, vdw, 2.96Å	Ala103, vdw, 3.45Å<br>Leu205, vdw, 3.31Å<br>Lys105, vdw, 3.44Å<br>Asp216, vdw, 3.3Å
**6b**	Met156, hbond, vdw, 2.95Å<br>Glu154, hbond, vdw, 3Å	Met153, vdw, 3.3Å<br>Ala103, vdw, 3.46Å
**6c**	Met156, hbond, vdw, 3.08Å<br>Glu154, hbond, vdw, 3.15Å	Val137, vdw, 3.35Å<br>Asp216, vdw, 3.14Å

**6d**	Met156, hbond, vdw, 3.07Å<br>Glu154, hbond, vdw, 3Å	Asp216, vdw, 3.23Å<br>Met153, vdw, 3.42Å<br>Leu205, vdw, 3.37Å<br>Ala103, vdw, 3.34Å
**6e**	Met156, hbond, vdw, 3.15Å<br>Ile82, vdw, 3.11Å	Val90, vdw, 3.39Å<br>Ala103, vdw, 3.38Å

*'hbond' – hydrogen bond, 'vdw' – van der Waals, 'weak hbond' – weak hydrogen bond.

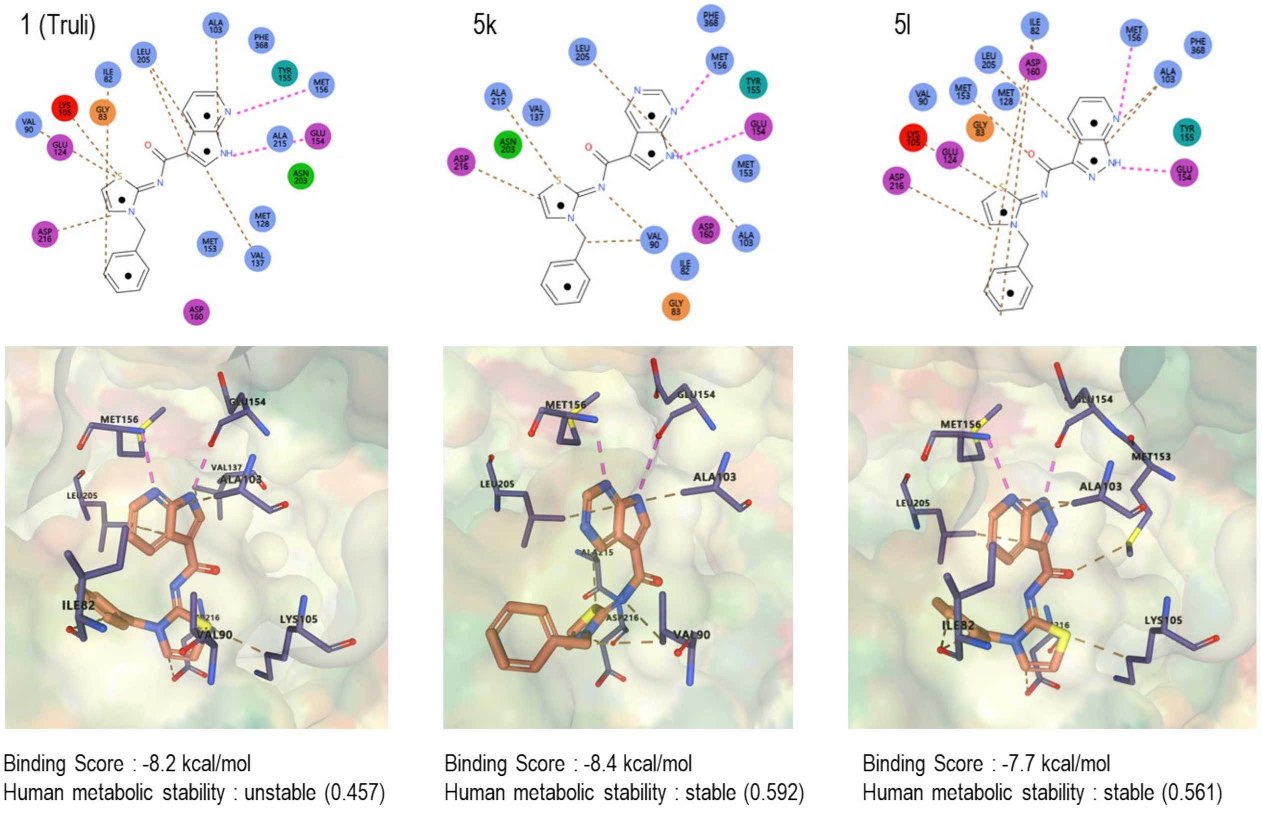

Figure S45. Molecular docking simulation of **1** (Truli), **5k** and **5l**.

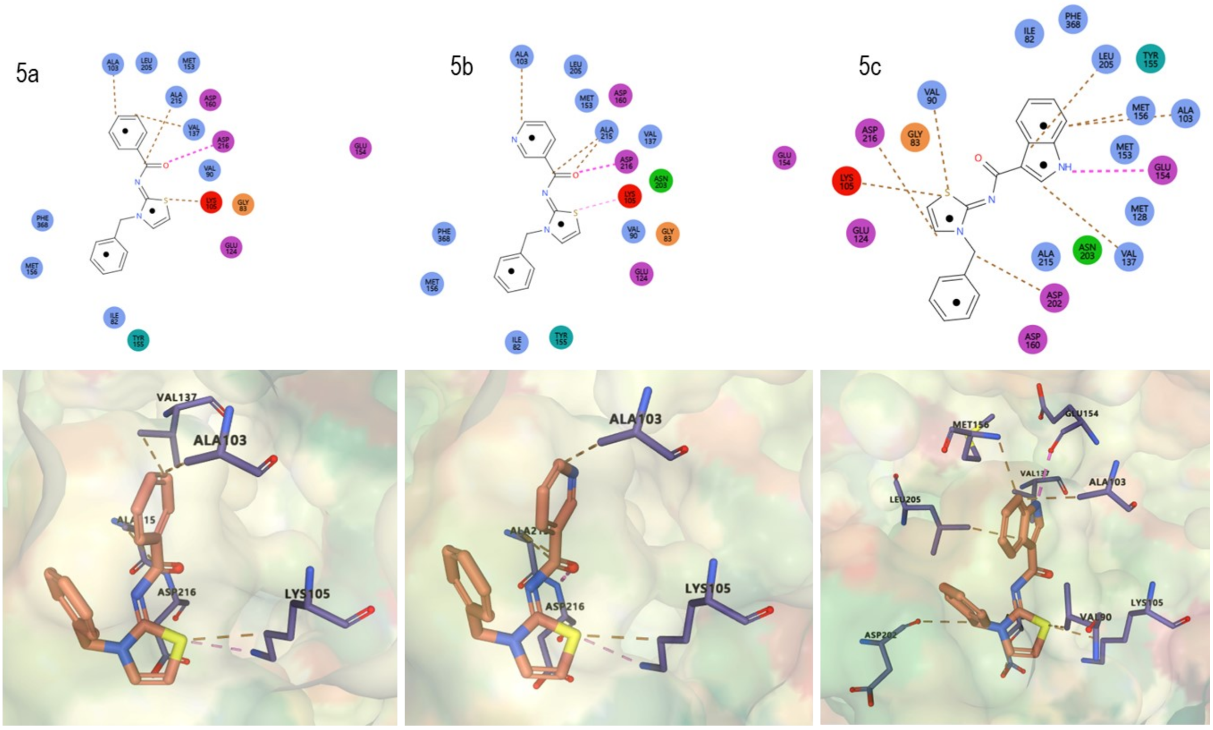

Binding Score : -4.0 kcal/mol

Binding Score : -5.5 kcal/mol

Binding Score : -5.9 kcal/mol

Figure S46. Molecular docking simulation of **5a**, **5b** and **5c**.

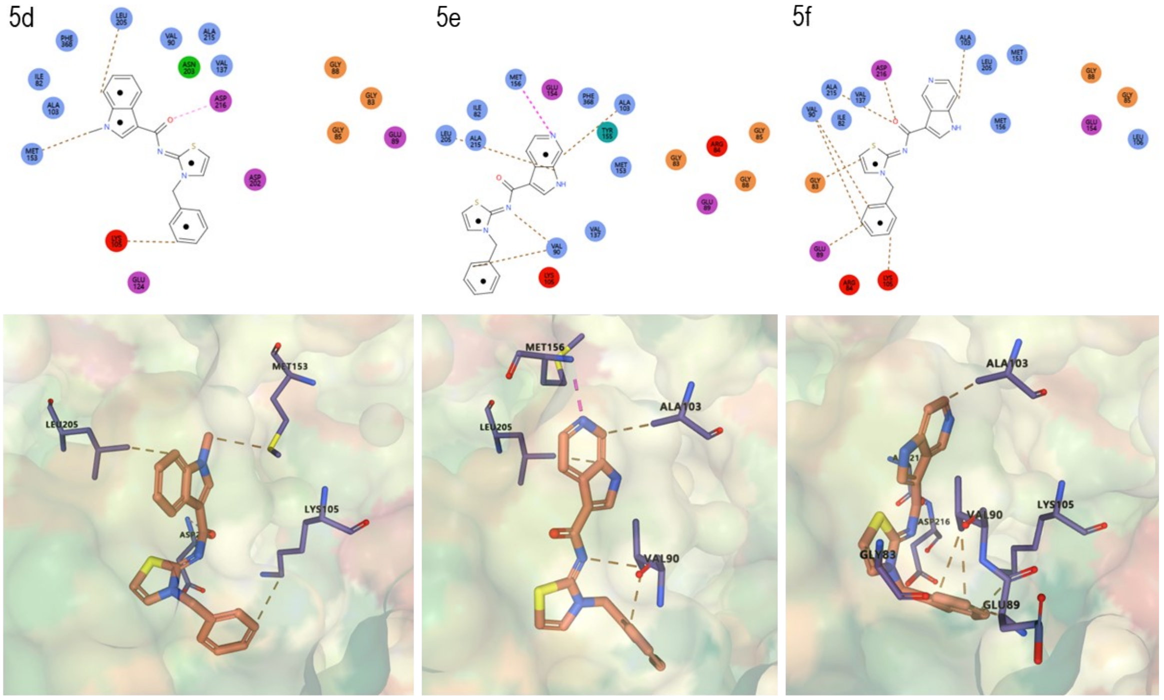

Binding Score : -4.5 kcal/mol

Binding Score : -7.3 kcal/mol

Binding Score : -6.5 kcal/mol

Figure S47. Molecular docking simulation of **5d**, **5e** and **5f**.

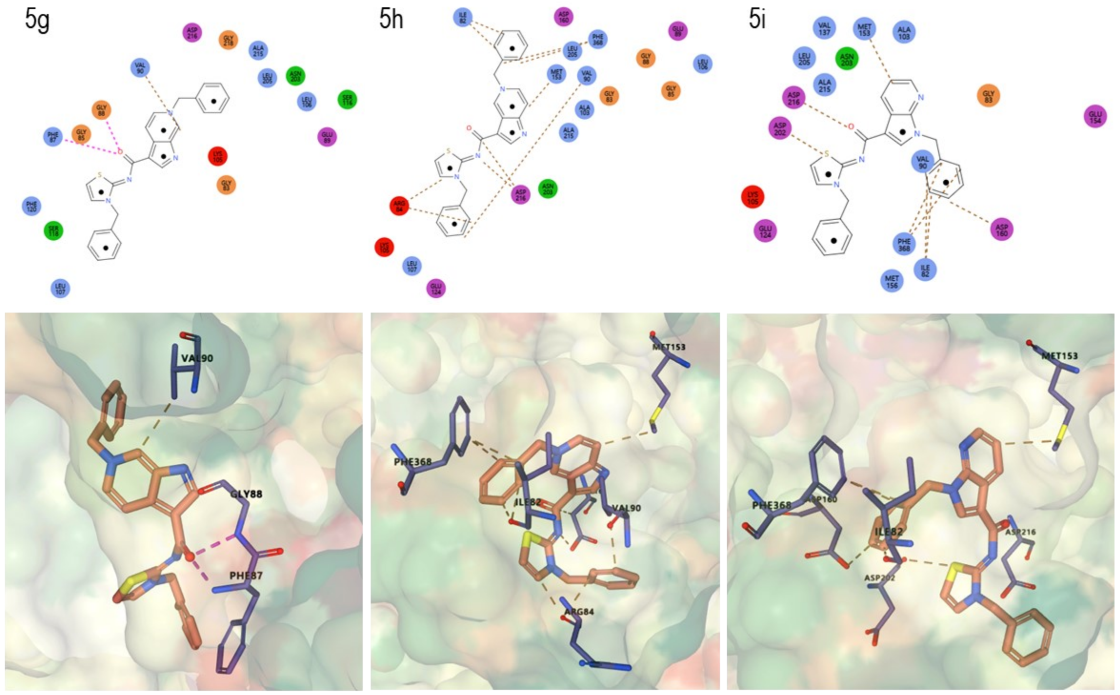

Binding Score : -6.3 kcal/mol Binding Score : -5.8 kcal/mol Binding Score : -5.7 kcal/mol

Figure S48. Molecular docking simulation of **5g**, **5h** and **5i**.

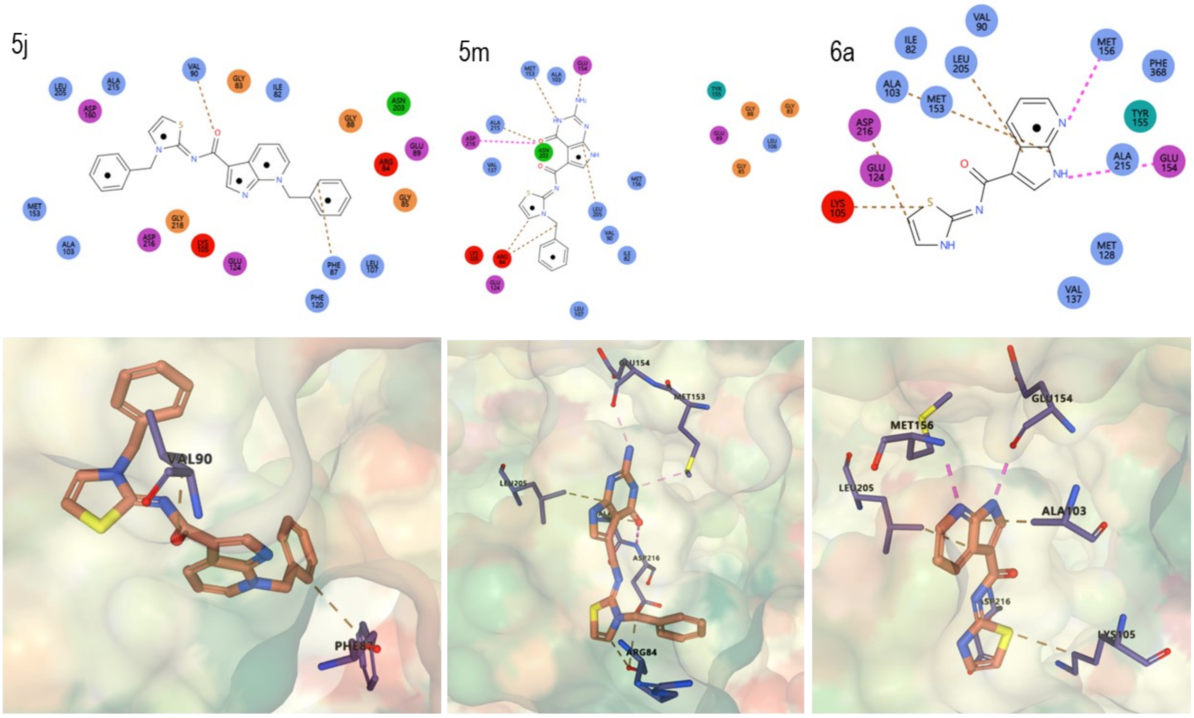

Binding Score : -4.8 kcal/mol Binding Score : -5.7 kcal/mol Binding Score : -7.4 kcal/mol

Figure S49. Molecular docking simulation of **5j**, **5m** and **6a**.

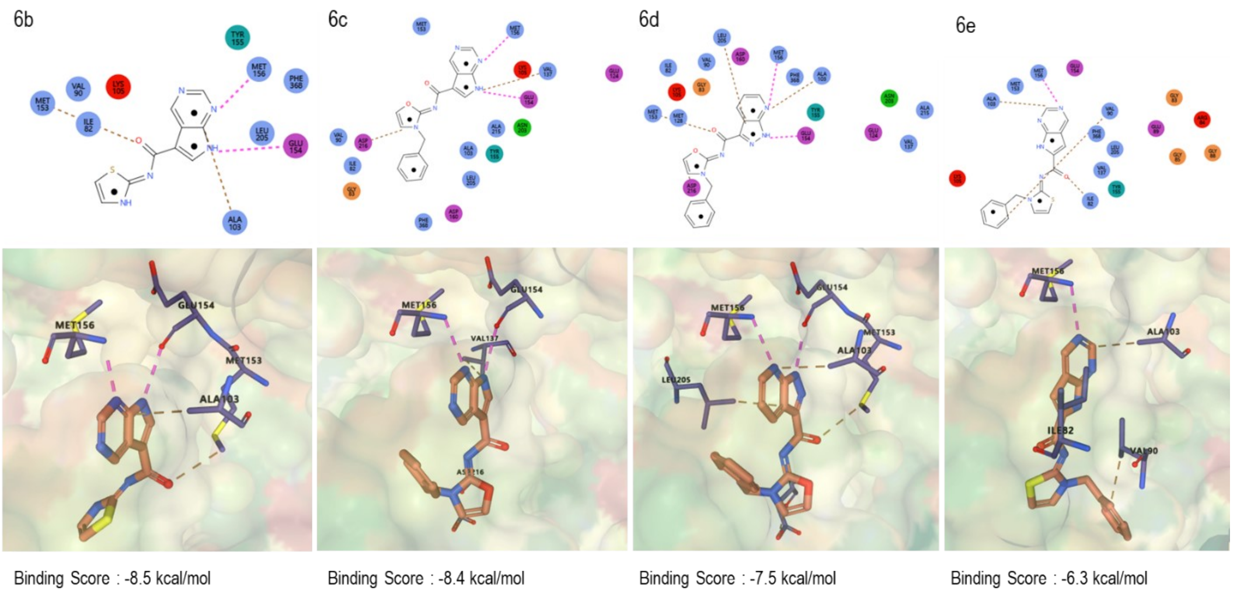

Figure S50. Molecular docking simulation of **6b**, **6c**, **6d** and **6e**.

10. References

1. Gnedeva K, Hudspeth AJ, Kastan N, Liang R, Meinke PT, Huggins DJ, et al. Pyrrolo [2,3-b]pyridine-3-carboxamide compositions and methods for ameliorating hearing loss. WO2021/158936A1 (Patent) 2021.
2. Dalziel ME, Patel JJ, Kaye MK, Cosman JL, Kitching MO, Snieckus V. Regioselective Functionalization of 7-Azaindole by Controlled Annular Isomerism: The Directed Metalation-Group Dance. *Angewandte Chemie International Edition*. 2019;58(22):7313-7317. doi:10.1002/anie.201901724.
3. Lin M, Tesconi M, Tischler M. Use of (1)H NMR to facilitate solubility measurement for drug discovery compounds. *International Journal of Pharmaceutics*. 2009;369(1-2):47-52. doi: 10.1016/j.ijpharm.2008.10.038.
4. Obach RS. Prediction of human clearance of twenty-nine drugs from hepatic microsomal intrinsic clearance data: An examination of in vitro half-life approach and nonspecific binding to microsomes. *Drug Metabolism and Disposition*. 1999;27(11):1350-1359.
5. Di L, Kerns EH, Hong Y, Kleintop TA, McConnell OJ, Huryn DM. Optimization of a higher throughput microsomal stability screening assay for profiling drug discovery

candidates. *Journal of Biomolecular Screening*. 2003;8(4):453-462. doi: 10.1177/1087057103255988.
6. Di L, Kerns EH. Drug-like properties: concepts, structure design and methods from ADME to toxicity optimization. Academic press; 2015.
7. Ryu JY, Lee JH, Lee BH, Song JS, Ahn S, Oh KS. PredMS: a random forest model for predicting metabolic stability of drug candidates in human liver microsomes. *Bioinformatics*. 2022;38(2):364-368. doi: 10.1093/bioinformatics/btab547.
8. Lim J, Ryu S, Park K, Choe YJ, Ham J, Kim WY. Predicting Drug-Target Interaction Using a Novel Graph Neural Network with 3D Structure-Embedded Graph Representation. *Journal of Chemical Information and Modeling*. 2019;59(9):3981-3988. doi: 10.1021/acs.jcim.9b00387.
9. Moon S, Zhung W, Yang S, Lim J, Kim WY. PIGNet: a physics-informed deep learning model toward generalized drug-target interaction predictions. *Chemical Science*. 2022;13(13):3661-3673. doi: 10.1039/d1sc06946b.
10. Bandarage UK, Cao J, Come JH, Court JJ, Gao H, Jacobs MD, et al. ROCK inhibitors 3: Design, synthesis and structure-activity relationships of 7-azaindole-based Rho kinase (ROCK) inhibitors. *Bioorganic and Medicinal Chemistry Letters*. 2018;28(15):2622-2626. doi: 10.1016/j.bmcl.2018.06.040.
